# HSPA12A maintains aerobic glycolytic homeostasis and Histone3 lactylation in cardiomyocytes to attenuate myocardial ischemia/reperfusion injury

**DOI:** 10.1172/jci.insight.169125

**Published:** 2024-02-29

**Authors:** Wansu Yu, Qiuyue Kong, Surong Jiang, Yunfan Li, Zhaohe Wang, Qian Mao, Xiaojin Zhang, Qianhui Liu, Pengjun Zhang, Yuehua Li, Chuanfu Li, Zhengnian Ding, Li Liu

**Affiliations:** 1 Department of Geriatrics, Jiangsu Provincial Key Laboratory of Geriatrics, and; 2 Department of Anesthesiology, First Affiliated Hospital of Nanjing Medical University, Nanjing, China.; 3 Department of Nuclear Medicine, Nanjing First Hospital of Nanjing Medical University, Nanjing, China.; 4 Key Laboratory of Targeted Intervention of Cardiovascular Disease, Collaborative Innovation Center for Cardiovascular Disease Translational Medicine, Nanjing Medical University, China.; 5 Departments of Surgery, East Tennessee State University, Johnson City, Tennessee, USA.

**Keywords:** Cardiology, Cell biology, Cardiovascular disease, Glucose metabolism, Hypoxia

## Abstract

Myocardial ischemia/reperfusion (MI/R) injury is a major cause of adverse outcomes of revascularization following myocardial infarction. Anaerobic glycolysis during myocardial ischemia is well studied, but the role of aerobic glycolysis during the early phase of reperfusion is incompletely understood. Lactylation of Histone H3 (H3) is an epigenetic indicator of the glycolytic switch. Heat shock protein A12A (HSPA12A) is an atypic member of the HSP70 family. In the present study, we report that, during reperfusion following myocardial ischemia, HSPA12A was downregulated and aerobic glycolytic flux was decreased in cardiomyocytes. Notably, HSPA12A KO in mice exacerbated MI/R-induced aerobic glycolysis decrease, cardiomyocyte death, and cardiac dysfunction. Gain- and loss-of-function studies demonstrated that HSPA12A was required to support cardiomyocyte survival upon hypoxia/reoxygenation (H/R) challenge and that its protective effects were mediated by maintaining aerobic glycolytic homeostasis for H3 lactylation. Further analyses revealed that HSPA12A increased Smurf1-mediated Hif1α protein stability, thus increasing glycolytic gene expression to maintain appropriate aerobic glycolytic activity to sustain H3 lactylation during reperfusion and, ultimately, improving cardiomyocyte survival to attenuate MI/R injury.

## Abstract 2

HSPA12A protects the heart against myocardial ischemia/reperfusion injury by maintaining aerobic glycolytic homeostasis and H3 lactylation in cardiomyocytes by increasing Smurf1-mediated Hif1&alpha; protein stabilization.

## Graphical abstract

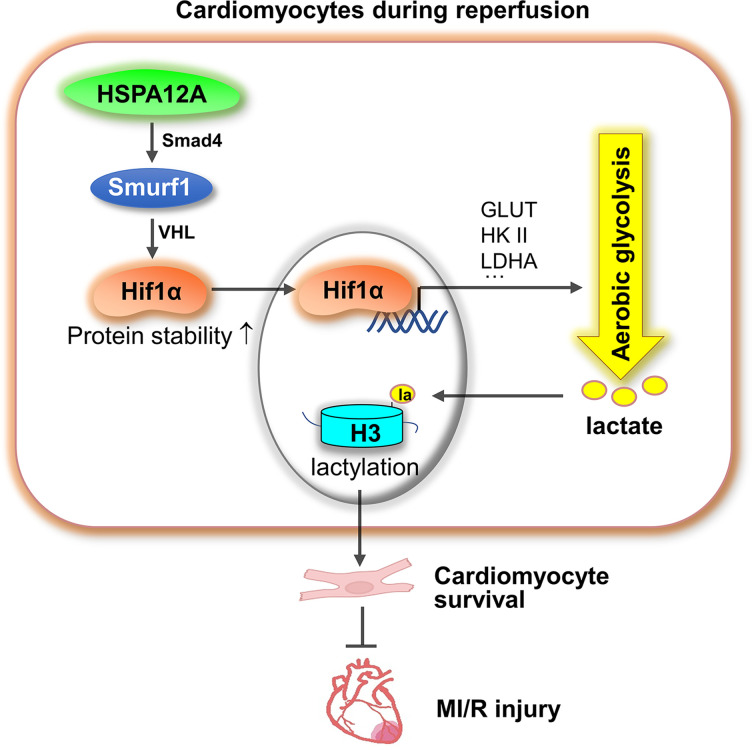

## Introduction

Acute myocardial infarction (MI) is the leading cause of morbidity and mortality worldwide, and timely reperfusion to restore coronary blood flow is the standard treatment for MI (1, 2). However, reperfusion can cause myocardial ischemia/reperfusion (MI/R) injury, a major cause of adverse outcomes of revascularization after MI. Although significant advances have improved MI/R injury outcomes, novel therapeutic interventions are an unmet clinical need.

Glycolysis is the metabolic process converting glucose into pyruvate that can subsequently be converted to lactate (3, 4). Glycolysis can be classified as aerobic or anaerobic, depending on the oxygen availability of the environment in which it occurs. In the normal adult heart, 5% of ATP is synthesized by aerobic glycolysis, and the remaining 95% is provided by mitochondrial oxidative phosphorylation (5, 6). However, under MI conditions, anaerobic glycolysis is induced as a compensatory response to oxygen deprivation, and increased glycolytic substrate availability is cardioprotective in the ischemic myocardium (7, 8). Intriguingly, aerobic glycolysis is activated in cardiac hypertrophy, heart failure, and diabetic cardiomyopathy (9–12). Specifically, reprogramming metabolism from fatty acid oxidation to glycolysis confers protection against MI/R (11). The transcription factor hypoxia-inducible factor 1α (Hif1α) induces glycolysis by transcriptionally activating expression of its target glycolytic genes (13, 14). However, the regulatory mechanisms for Hif1α and aerobic glycolysis during the reperfusion following myocardial ischemia is incompletely understood.

Although glycolysis plays a critical role in diverse physiological and pathological events, the underlying mechanisms by which glycolytic flux affects disease pathology remain elusive. Interestingly, lactylation of some proteins could represent an operative mechanism by which glycolysis modulates some pathophysiological events, as demonstrated in recent studies (15–17). Lactylation is a posttranslational modification in which p300 catalyzes binding of glycolysis-derived lactyl groups to the lysine residues of target proteins (15). Histone lactylation is considered to be an epigenetic marker of the glycolytic switch. Interestingly, Histone lactylation modulates macrophage activation and promotes oncogenesis in some contexts (15–17). Specifically, lactylation of H3 and α-myosin heavy chain (α-MHC) is cardioprotective in the contexts of MI injury and heart failure (18, 19). However, the role of H3 lactylation in MI/R injury and the regulatory mechanisms of H3 lactylation following MI/R are incompletely understood.

Heat shock protein A12A (HSPA12A) was identified in 2003 and classified as an atypical member of the HSP70 family (20). Subsequent studies demonstrated potential involvement of HSPA12A in schizophrenia (21). We recently reported that HSPA12A plays critical roles in metabolic regulation in the contexts of obesity and nonalcoholic fatty liver diseases (22, 23). Specifically, we demonstrated that HSPA12A activates aerobic glycolysis in liver cancer cells but suppresses aerobic glycolysis in renal cancer cells (4, 24), suggesting that HSPA12A glycolytic regulation is tissue specific. However, whether HSPA12A modulates aerobic glycolysis in cardiomyocytes to regulate MI/R injury remains unknown.

In the present study, we demonstrated that HSPA12A was downregulated and aerobic glycolysis was inhibited in MI/R hearts during reperfusion, while HSPA12A ablation in mice exacerbated MI/R injury. Further gain- and loss-of-function experiments demonstrated that HSPA12A supported cardiomyocyte survival upon H/R challenge via maintaining aerobic glycolytic homeostasis to sustain H3 lactylation by increasing Smurf1-mediated Hif1α protein stability. Thus, cardiomyocyte HSPA12A has therapeutic potential for management of MI/R injury.

## Results

### MI/R decreased HSPA12A expression levels and aerobic glycolytic flux in cardiomyocytes

**Figure 1:**
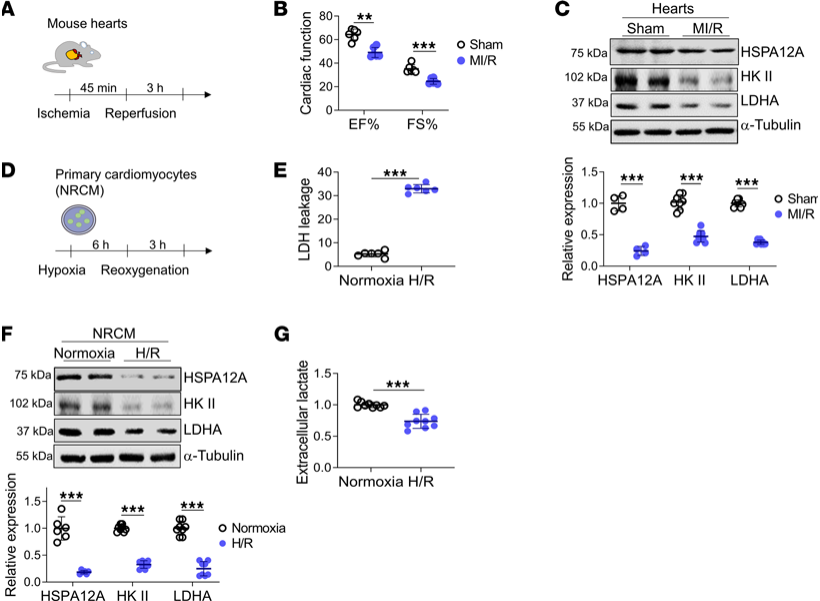
Downregulation of HSPA12A was accompanied by aerobic glycolysis decrease in cardiomyocytes during reperfusion period after ischemia. (**A**–**C**) Mouse experiments. MI/R was induced in adult mice (**A**). After MI/R, cardiac function in mice was examined by echocardiography (**B**), and the indicated gene expression in myocardial infarcts was examined by immunoblotting (**C**). *n* = 6/group (**B**), *n* = 4/group for HSPA12A analysis and *n* = 8/group for HK-II and LDHA analyses (**C**). (**D**–**G**) Cardiomyocyte experiments. Primary cardiomyocytes (NRCM) were subjected to H/R (**D**). After H/R, expression of the indicated genes in NRCM was examined by immunoblotting, and LDH activities and lactate levels were measured in culture medium (**E**–**G**). *n* = 6/group (**E**), *n* = 6/group for HSPA12A analysis and *n* = 8/group for HK-II and LDHA analyses (**F**), and *n* = 9/group (**G**). Data are shown as mean ± SD. ***P < 0.001 and **P < 0.01 by Mann-Whitney *U* test (**B**) or Student’s 2-tailed unpaired *t* test (**C**–**G**).

To evaluate the potential involvement of HSPA12A in MI/R injury, we examined HSPA12A protein expression in hearts of mice with MI/R (Figure 1A)**.** When MI/R caused cardiac injury, as indicated by decreased percentage of ejection fraction (EF%) and fraction shortening (FS%), HSPA12A expression levels were decreased in the infarcted myocardium during the reperfusion period following ischemia (Figure 1, B and C). Meanwhile, expression levels of glycolysis-related genes, including HK-II and LDHA, were also downregulated (Figure 1C).

To determine if downregulation of HSPA12A and glycolysis-related genes in MI/R hearts was attributable to cardiomyocytes, an in vitro MI/R model was simulated in primary cardiomyocytes (neonatal rat cardiomyocytes [NRCM]) by hypoxia/reoxygenation (H/R) (Figure 1D). Consistent with the findings in MI/R hearts, when H/R caused NRCM injury, as indicated by an elevated release of lactate dehydrogenase (LDH), expression levels of HSPA12A and glycolysis-related genes (HK-II and LDHA) were also downregulated during reoxygenation (Figure 1, E and F). Moreover, extracellular lactate, a glycolysis readout, was decreased in H/R cultures relative to normoxic controls (Figure 1G). H/R did not decrease expression levels of other heat shock proteins, including HSP25, HSP32, and HSP90, in NRCM H/R injury (Supplemental Figure 1A; supplemental material available online with this article; https://doi.org/10.1172/jci.insight.169125DS1). Also, in cardiac fibroblasts, HSPA12A expression was not affected by H/R (Supplemental Figure 1B).

### HSPA12A downregulation contributed to MI/R injury

**Figure 2:**
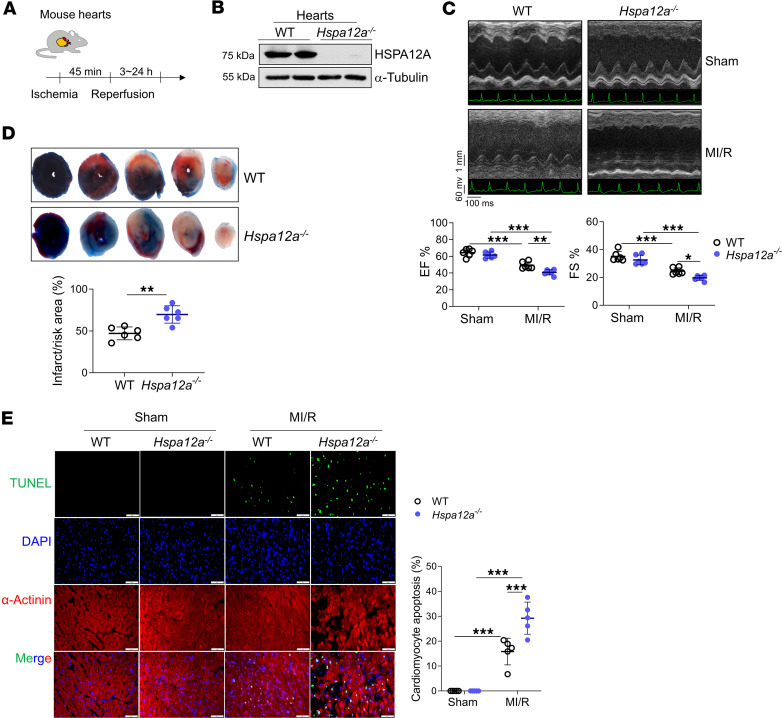
Downregulation of HSPA12A contributed to MI/R injury. (**A**) Mouse experimental protocol. (**B**) The absence of HSPA12A expression in hearts of *Hspa12a^–/–^* mice was demonstrated by immunoblotting. *n* = 10 mice/group. (**C**) Cardiac function was evaluated 3 hours after MI/R by echocardiography. *n* = 6/group. (**D**) Infarct size was examined 24 hours after MI/R. The infarct regions were shown as pale white with TTC staining. The ischemia risk area was illustrated by Evan’s blue nonstained areas. *n* = 6 mice/group. (**E**) Apoptosis in cardiomyocytes was examined 3 hours after MI/R by TUNEL assay on the paraffin-embedded sections that prepared from cardiac tissues at papillary muscles. α-Actinin was used to stain cardiomyocytes, and DAPI was used to counterstain the nuclei. Scale bars: 50 μm. *n* = 5/group. Data are shown as mean ± SD. ***P < 0.001, **P < 0.01, and *P<0.05 by 2-way ANOVA followed by post hoc test (**C** and **E**) or Student’s 2-tailed unpaired *t* test (**D**).

To investigate whether downregulation of HSPA12A contributed to MI/R injury, we subjected HSPA12A-KO mice (*Hspa12a^–/–^*) and WT littermates to MI/R surgery (Figure 2A). Ablation of HSPA12A in hearts of *Hspa12a^–/–^* mice was shown in Figure 2B. At basal (sham) levels, cardiac systolic function (EF% and FS%), left ventricle chamber size (left ventricular internal diameter at diastolic phase [LVIDd], left ventricular internal diameter at systolic phase [LVIDs], left ventricular end-diastolic volume [LVVd], and left ventricular end-systolic volume [LVVs]), and left ventricle wall thickness (interventricular septum thickness at diastolic phase [IVSd], interventricular septum thickness at systolic phase [IVSs], left ventricular posterior wall thickness at diastolic phase [LVPWd], and left ventricular posterior wall thickness at systolic phase [LVPWs]) were similar in *Hspa12a^–/–^* and WT mice (Figure 2C and Supplemental Figures 2 and 3). However, cardiac function measured 3 hours after reperfusion demonstrated that the MI/R-induced decreases of EF% and FS% were further exacerbated in *Hspa12a^–/–^* mice relative to WT mice (Figure 2C). Furthermore, exacerbation of cardiac systolic dysfunction in *Hspa12a^–/–^* mice persisted for at least 7 days following reperfusion (Supplemental Figure 3).

Consistent with compounded cardiac dysfunction in *Hspa12a^–/–^* MI/R mice, infarct size was larger in *Hspa12a^–/–^* MI/R mice relative to WT controls (Figure 2D). Moreover, MI/R-induced cardiomyocyte apoptosis (TUENL^+^/α-actinin^+^) was increased in *Hspa12a^–/–^* mice relative to WT mice (Figure 2E). Collectively, these findings demonstrate that downregulation of HSPA12A contributed to MI/R injury.

To further determine the role of cardiomyocyte HSPA12A in MI/R injury, we generated knockin mice with cardiac-specific overexpression of HSPA12A (*Hspa12a^ki^*) by cross-breeding conditional *Hspa12a*-knockin mice (*Hspa12a^fl/fl^*) with *Myh6-Cre* transgenic mice (*Myh6-Cre^Tg/+^*) (Supplemental Figure 4A). The strategy for creating conditional *Hspa12a*-knockin mice was shown in our recent study (25). Compared with WT controls, HSPA12A was overexpressed in the heart, but not in liver, lung, kidney, or brain tissues of *Hspa12a^ki^* mice (Supplemental Figure 4B). Echocardiographic examination demonstrated that MI/R-induced decrease of cardiac systolic function (EF% and FS%) was attenuated in *Hspa12a^ki^* mice compared with WT mice (Supplemental Figure 5A). Also, MI/R-induced increase of left ventricular volume at end systolic phase (LVVs) was attenuated in *Hspa12a^ki^* mice relative to WT. Moreover, MI/R increased cardiomyocyte apoptosis in both genotypes, but apoptosis was alleviated in *Hspa12a^ki^* mice relative to WT (Supplemental Figure 5B).

### Essential role of HSPA12A in supporting cardiomyocyte survival upon H/R challenge

**Figure 3:**
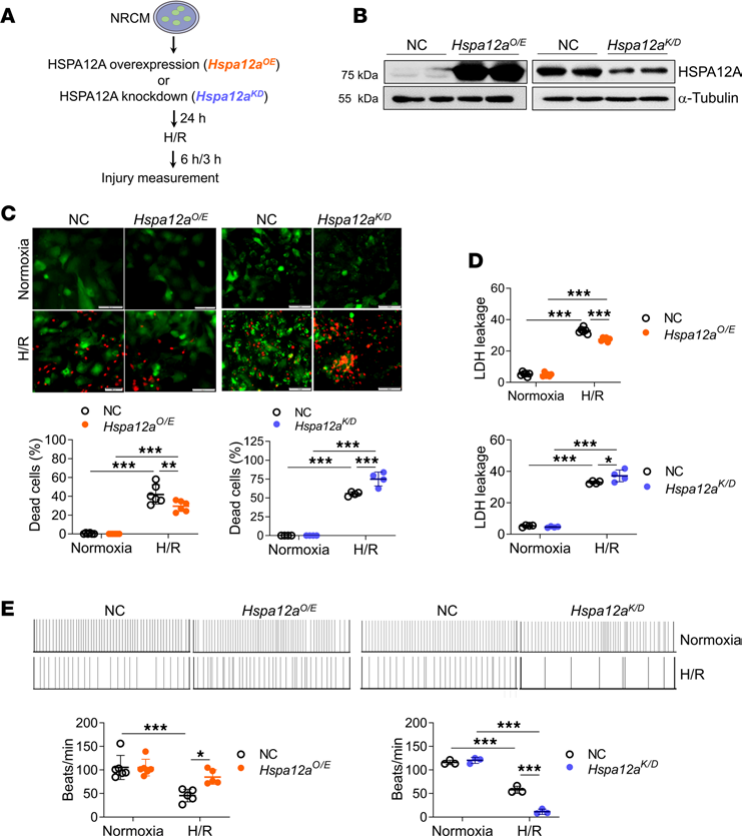
HSPA12A was required for supporting cardiomyocyte survival against H/R challenge. (**A**) Cardiomyocyte experimental protocol. (**B**) Overexpression or knockdown of HSPA12A in NRCM was confirmed using immunoblotting. (**C**) NRCM death was examined by live/dead assay as reflected by 2-color fluorescence based on living (green) and dead (red) cells. NRCM death was expressed as a percentage of dead cells to total cells. Scale bars: 50 μm. *n* = 6/group for left panel, and *n* = 4/group for right panel. (**D**) LDH leakage. *n* = 6/group for up panel, and *n* = 4/group for down panel. (**E**) The beating frequency of NRCM was recorded for 30 seconds. *n* = 5 for normoxia groups, *n* = 6 for H/R groups in left panel, and *n* = 3/group for right panel. Data are shown as mean ± SD. ***P < 0.001, **P < 0.01, and *P < 0.05 by 2-way ANOVA followed by post hoc test.

Cardiomyocyte death is a critical determinant of MI/R injury (26). To determine if HSPA12A protects cardiomyocytes against MI/R, an in vitro MI/R model was simulated in NRCM (Figure 3A). HSPA12A was overexpressed (*Hspa12a^O/E^*) or knocked down (*Hspa12a^K/D^*) in NRCM (Figure 3B). H/R evoked NRCM injury in all groups, as illustrated by increased cell death and LDH release as well as morphological abnormalities (Figure 3, C and D, and Supplemental Figure 6, A and B). However, the H/R-evoked NRCM injury was attenuated by HSPA12A overexpression but further exacerbated by HSPA12A knockdown, relative to their negative controls (NC). Also, a H/R-induced decrease in NRCM beating frequency was improved by HSPA12A overexpression but exacerbated by HSPA12A knockdown (Figure 3E). Additionally, HSPA12A overexpression attenuated H/R-induced Bcl-2 downregulation, while HSPA12A knockdown showed the opposite effects (Supplemental Figure 6C). Taken together, these findings demonstrate that HSPA12A protected cardiomyocytes against H/R challenge.

### HSPA12A maintained aerobic glycolytic homeostasis to protect cardiomyocytes against H/R challenge

Metabolic reprogramming to aerobic glycolysis confers cardioprotection against heart failure (9, 11). We, thus, examined whether glycolysis is involved in HSPA12A-mediated cardioprotection against MI/R injury (Supplemental Figure 7A). Cardiac expression of HSPA12A and glycolysis-related genes (GLUT4 and LDHA) decreased 1 hour after reperfusion, and these decreases persisted for at least 24 hours following reperfusion (Supplemental Figure 7B). Notably, the MI/R-induced downregulation of glucose uptake genes (GLUT1 and GLUT4) and glycolytic enzymes (HK-II and LDHA) was further exaggerated in *Hspa12a^–/–^* hearts, but downregulation of this gene set was alleviated in *Hspa12a^ki^* hearts relative to the respective WT controls (Supplemental Figure 7C and Supplemental Figure 8). To further investigate the effect of HSPA12A on glucose metabolism in intact MI/R-injured hearts, we utilized the glucose analog ^18^F-FDG in conjunction with positron emission tomography/computed tomography (PET/CT) according to previous studies (27). ^18^F-FDG images at short and horizontal long axis showed that, following MI/R, the uptake was absent in lateral and anteroapical regions of hearts; however, the MI/R-induced uptake-absent areas were further enlarged in *Hspa12a^–/–^* hearts compared with WT mice. Moreover, ^18^F-FDG images at the vertical long axis showed an uptake-absent area in *Hspa12a^–/–^* hearts but not in WT mice following MI/R. Total ^18^F-FDG uptake activity, which was measured as the percent injected dose per gram of tissue (%ID/g), was decreased in MI/R-*Hspa12a^–/–^* hearts relative to MI/R-WT hearts (Supplemental Figure 9, A–C).

**Figure 4:**
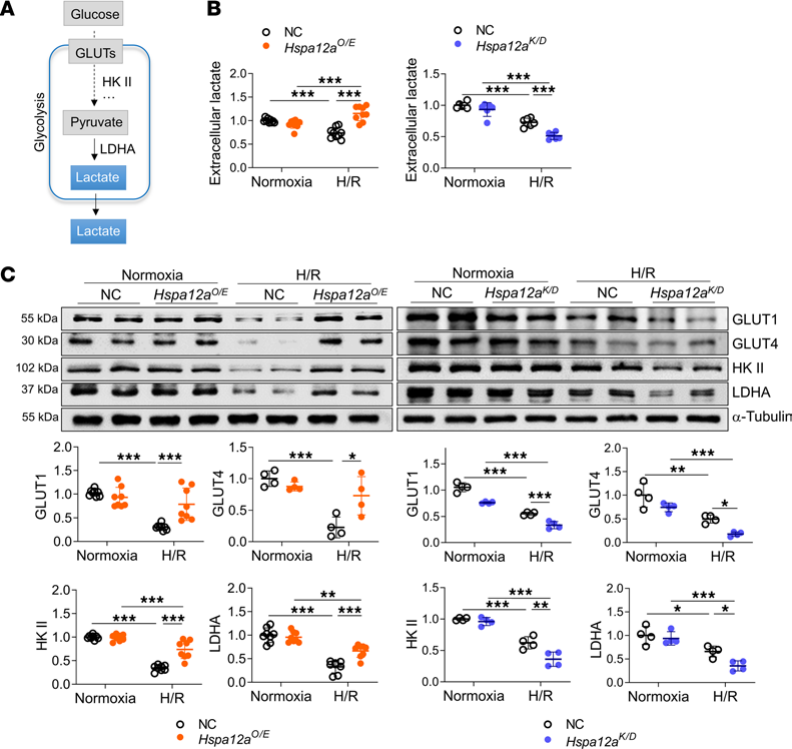
HSPA12A maintained aerobic glycolytic homeostasis to protect cardiomyocytes against H/R challenge. (**A**) Brief scheme of glycolytic process. (**B**) Extracellular lactate was examined in culture medium. The lactate values were expressed as relative contents to the normoxia NC controls. *n* = 6–9/group. (**C**) Expression of the indicted genes in NRCM was examined using immunoblotting. *n* = 4–8/group. Data are shown as mean ± SD. ***P < 0.001, **P < 0.01, and *P < 0.05 by 2-way ANOVA followed by post hoc test.

Glycolysis is the metabolic process that converts glucose into pyruvate, which can be subsequently converted to lactate (3). Because lactate is exported into the extracellular space, extracellular lactate is considered a readout of glycolytic flux (3, 4). To examine whether aerobic glycolysis during reoxygenation is related to the cardioprotective effects of HSPA12A, we measured extracellular lactate levels in the primary cardiomyocyte (NRCM) culture model (Figure 4A). Notably, H/R decreased extracellular lactate in the NC group, a reduction that was prevented by HSPA12A overexpression (Figure 4B). H/R decreased cardiomyocyte glucose uptake, as reflected by 2-NBDG labeling, whereas this reduction was attenuated in *Hspa12a^O/E^* NRCM relative to NC NRCM (Supplemental Figure 10, A and B). Similarly, extracellular flux analysis revealed that H/R decreased extracellular acidification rates (ECAR) and glycolytic capacity in NC NRCM; however, the decreases were prevented in *Hspa12a^O/E^* NRCM (Supplemental Figure 10, C and D). In support of this, HSPA12A overexpression in NRCM attenuated H/R-induced downregulation of GLUT1, GLUT4, HK-II, and LDHA relative to NC NRCM (Figure 4C). Contrastingly, HSPA12A knockdown further exacerbated H/R-induced decreases of extracellular lactate levels (Figure 4B) and glycolysis-related gene expression (Figure 4C). Though H/R decreased oxygen consumption rate (OCR) in NRCM, but the decrease did not differ between *Hspa12a^O/E^* NRCM and NC NRCM (Supplemental Figure 10, E and F). Collectively, these findings demonstrate that HSPA12A maintained aerobic glycolytic homeostasis in cardiomyocytes during reoxygenation following hypoxia.

**Figure 5:**
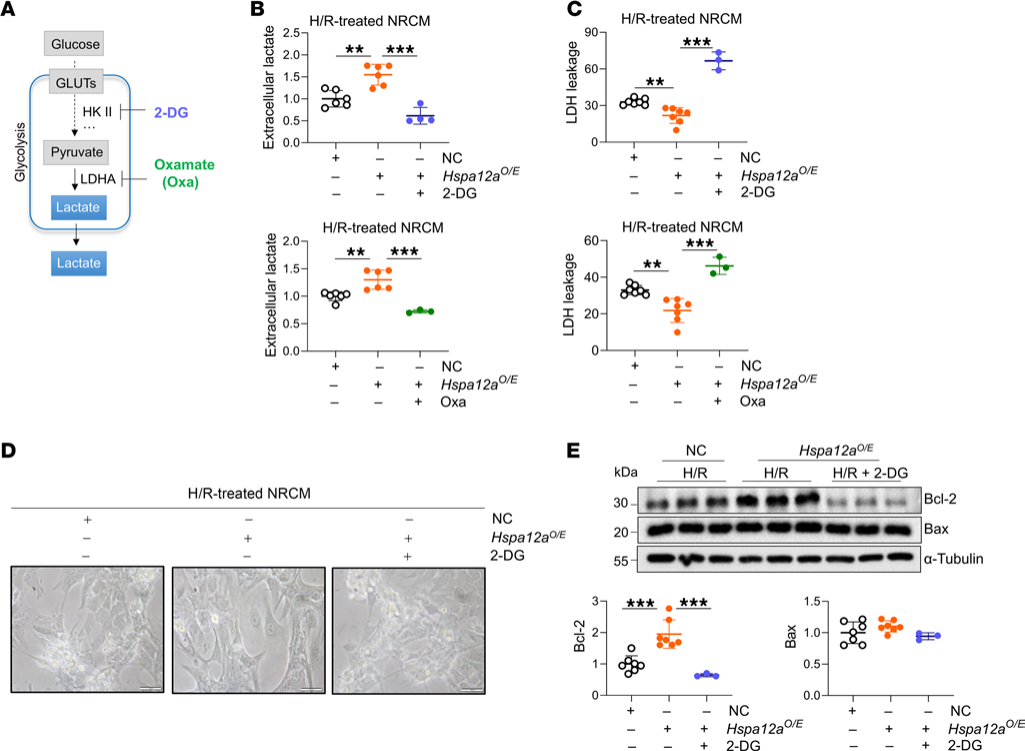
Glycolysis inhibition abolished the cardioprotection of HSPA12A against H/R challenge. (**A**) Brief scheme of glycolytic inhibitor treatment. 2-Deoxy-D-glucose (2-DG) and oxamate (Oxa) were introduced to NRCM culture during reperfusion. (**B**) Extracellular lactate levels were examined in culture medium. *n* = 3 – 6/group. (**C**) LDH leakage was examined in culture medium. *n* = 7 for NC group and for *Hspa12a^O/E^* group, *n* = 3 for “*Hspa12a^O/E^* + 2-DG” group and for “*Hspa12a^O/E^* + Oxa” group. (**D**) Morphological alterations were examined using a phase-contrast microscope; total original magnification, ×200 . Scale bars: 100 μm. *n* = 5/group. (**E**) The indicated gene expression was examined by immunoblotting analysis. *n* = 7 for NC group and for *Hspa12a^O/E^* group, and *n* = 3 for “*Hspa12a^O/E^* + 2-DG” group. Data are mean ± SD. ***P < 0.001 and **P < 0.01 using ordinary 1-way ANOVA followed by post hoc test.

To determine if maintenance of aerobic glycolytic homeostasis mediates the cardioprotective effect of HSPA12A, glycolysis was blocked with 2-Deoxy-D-glucose (2-DG) or oxamate (Oxa) in NRCM during reoxygenation as described previously (15, 28) (Figure 5A). Remarkably, when 2-DG removed the HSPA12A-induced increase of extracellular lactate (Figure 5B), the protective effects of HSPA12A against H/R injury, as indicated by attenuated LDH release and morphological abnormalities, were also reversed by 2-DG (Figure 5, C and D). Moreover, 2-DG abolished a HSPA12A-mediated increase of Bcl-2 protein levels in H/R NRCM (Figure 5E). Similarly, treatment with Oxa ablated a HSPA12A-mediated increase of extracellular lactate and decrease of LDH release (Figure 5, B and C) in H/R NRCM. Taken together, these findings demonstrate that HSPA12A protected cardiomyocytes against H/R challenge by maintaining aerobic glycolysis during the reoxygenation period following hypoxia.

Considering that myocardial glycogen storage is cardioprotective in the context of MI/R injury (29, 30), we examined effects of HSPA12A on glycogen content of the MI/R heart by periodic acid–Schiff (PAS) staining and assay kit. Although MI/R decreased heart glycogen content, glycogen levels did not differ between *Hspa12a^–/–^* and WT hearts following MI/R (Supplemental Figure 11, A–C).

### HSPA12A maintained H3 lactylation at lysine 56 (H3K56la) in H/R cardiomyocytes

**Figure 6:**
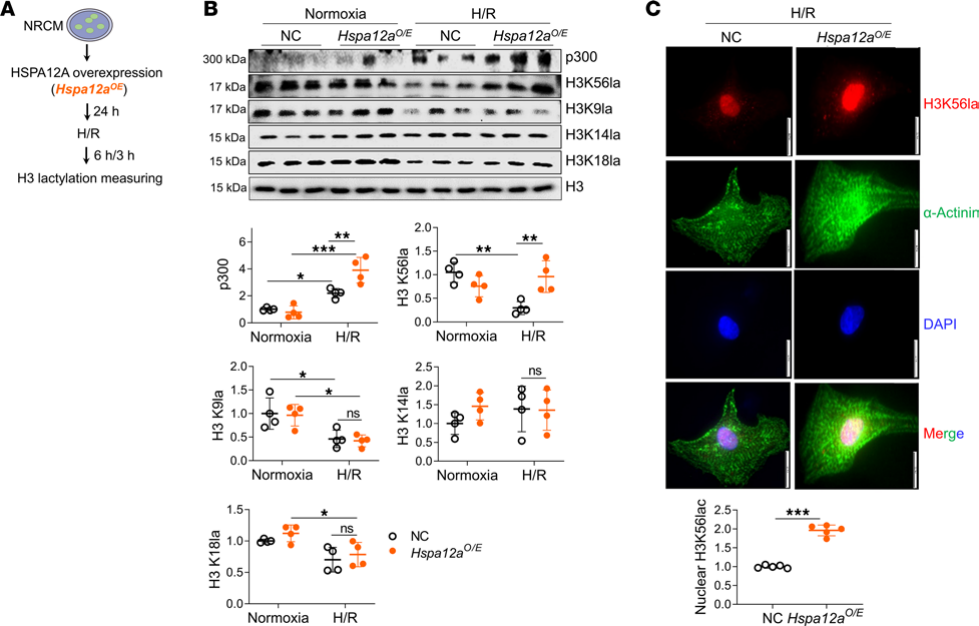
HSPA12A maintained H3 lactylation levels in H/R cardiomyocytes. (**A**) Experimental protocol. (**B**) H3 lactylation and p300 expression were examined in whole-cell lysates using immunoblotting. Data are shown as mean ± SD. ***P < 0.001, **P < 0.01, and *P < 0.05 by 2-way ANOVA followed by post hoc test. *n* = 4/group. (**C**) H3 lactylation at lysine 56 (H3K56la) was examined by immunofluorescence staining. α-Actinin was used to stain cardiomyocytes, and DAPI was used to counterstain the nuclei. Scale bars: 20 μm. Data are shown as mean ± SD. ***P < 0.001 by unpaired *t* test with Welch’s correction (*t* = 14.48, *df* = 4.631). *n* = 5/group.

Lactylation is a recently identified posttranslational modification in which p300 catalyzes binding of glycolysis-derived lactyl groups to protein lysine residues (15). H3 lactylation modulates macrophage activation and drives oncogenesis (16, 31). However, the role of H3 lactylation in cardiomyocytes is unknown. To determine the potential involvement of H3 lactylation in HSPA12A-mediated cardioprotection, NRCM H3 lactylation following H/R was examined (Figure 6A). Consistent with decreased lactate production following H/R (Figure 4B), H3K56la and H3K9la were decreased in the NC group following H/R (Figure 6B). However, H/R-induced decrease of H3K56la, but not H3K9la, was attenuated in *Hspa12a^O/E^* NRCM relative to NC NRCM (Figure 6B). This finding was further confirmed by immunofluorescence staining, which revealed increased nuclear H3K56la levels in H/R-treated *Hspa12a^O/E^* NRCM relative to H/R-treated NC NRCM (Figure 6C). H3K14la and H3K18la was similar in H/R-treated *Hspa12a^O/E^* and H/R-treated NC NRCM (Figure 6B). H/R increased p300 expression, which was enhanced in the *Hspa12a^O/E^* group relative to NC (Figure 6B). Taken together, these findings demonstrate that HSPA12A increase H3K56la in NRCM following H/R.

### Inhibition of H3 lactylation abolished cardioprotective effects of HSPA12A

**Figure 7:**
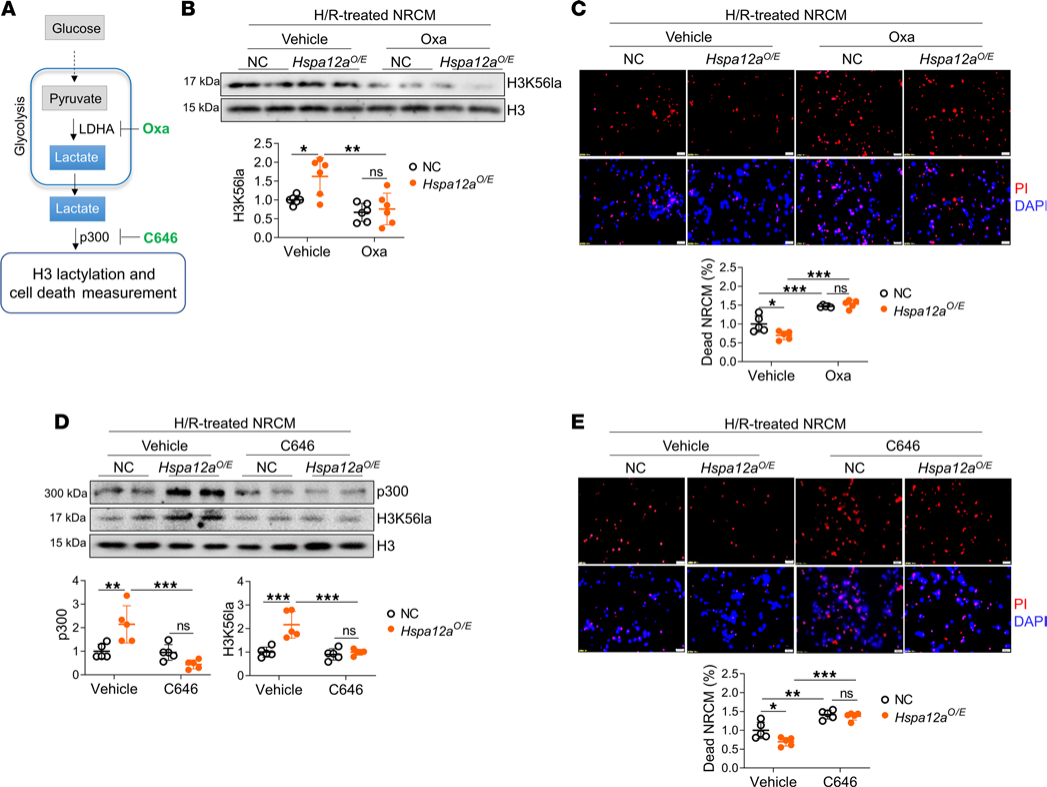
Inhibition of H3 lactylation abolished the cardioprotective role of HSPA12A against H/R challenge. (**A**) Experimental protocol. Two inhibitors, oxamate (Oxa) and C646, were introduced to NRCM culture during reoxygenation. (**B**) Lactylation of H3 at lysine 56 (H3K56la) in whole-cell lysates was examined using immunoblotting. The blots for H3 served as loading controls. *n* = 6/group. (**C**) NRCM death was examined using PI staining. DAPI was used to counter stain nuclei. *n* = 5/group. (**D**) Lactylation of H3 at lysine 56 (H3K56la) and expression of p300 expression in whole-cell lysates were examined using immunoblotting. The blots for H3 served as loading controls. *n* = 5/group. (**E**) NRCM death was examined using PI staining. DAPI was used to counter stain nuclei. *n* = 5/group. Data are shown as mean ± SD. ***P < 0.001, **P < 0.01, and *P < 0.05 by 2-way ANOVA followed by post hoc test.

To determine the role of H3 lactylation in HSPA12A-mediated cardioprotection, we blocked H3 lactylation using either Oxa to inhibit lactate production or C646 to inhibit p300, which catalyzes binding of lactyl groups to H3 lysine residues (Figure 7A). When Oxa was used to prevent the increase of lactate production in H/R-treated *Hspa12a^O/E^* NRCM (Figure 5B), the increase of H3K56la level was also abolished (Figure 7B). Importantly, Oxa treatment also ablated the prosurvival effects of HSP12A in H/R-treated NRCM (Figure 7C). Treatment with C646 to inhibit p300 had similar effects. C646 prevented p300 upregulation in H/R-treated NRCM (Figure 7D), which was consistent with the previous reports (32). Remarkably, when C646 prevented H3K56la upregulation in H/R-treated *Hspa12a^O/E^* NRCM (Figure 7D), the protective effects of HSP12A on NRCM survival were also abolished (Figure 7E). Together, these findings indicate that the cardioprotective effects of HSP12A against H/R challenge were dependent on H3 lactylation.

### HSPA12A maintained aerobic glycolysis by increasing Hif1α protein levels in H/R-treated cardiomyocytes

#### HSPA12A increased Hif1α protein levels and nuclear localization in H/R-treated cardiomyocytes.

**Figure 8:**
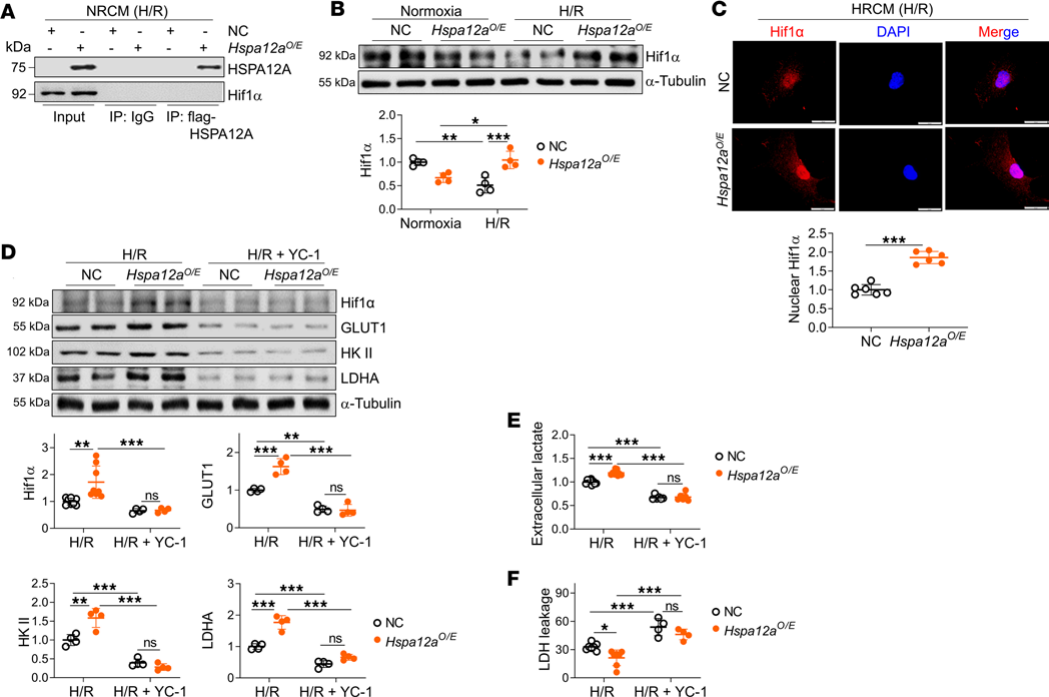
Hif1α meditated the HSPA12A-maintained aerobic glycolysis of NRCM upon H/R challenge. (**A**) IP immunoblotting was performed to examined interaction between HSPA12A and Hif1α in H/R NRCM. HSPA12A immunoprecipitates were prepared from H/R-treated NRCM and subsequently subjected to immunoblotting against HSPA12A and Hif1α. Input served as positive controls, and IgG immunoprecipitates served as negative controls. (**B**) Total Hif1α protein expression in whole-cell lysates was examined by immunoblotting. *n* = 4/group. (**C**) Nuclear contents of Hif1α were examined by immunostaining in H/R-treated NRCM. DAPI was used to counterstain nuclei. Data were expressed as averaged Hif1α fluorescence intensity in nucleus. *n* = 6/group. Scale bars: 10 μm. (**D**) Expression of the indicated genes were examined by immunoblotting. *n* = 4/group, except for *n* = 8 for Hif1α examination in H/R groups. (**E**) Extracellular lactates were examined in culture medium of NRCM. *n* = 9 for H/R groups, and *n* = 6 for “H/R + YC-1” groups. (**F**) LDH leakage was examined. *n* = 7 for H/R groups, and *n* = 4 for “H/R + YC-1” groups. Data are shown as mean ± SD. ***P < 0.001, **P < 0.01, and *P < 0.05 by 2-way ANOVA followed by post hoc test (**B** and **D**–**F**) or Student’s 2-tailed unpaired *t* test (**C**).

Glycolysis is catalyzed by a gene set that is transcriptionally regulated by multiple transcription factors, including Hif1α (33, 34). To determine whether Hif1α contributed to HSPA12A-regulated aerobic glycolysis, we first performed an IP immunoblotting assay and did not detect direct interaction between HSPA12A and Hif1α in H/R-treated NRCM (Figure 8A). However, HSPA12A overexpression in NRCM completely prevented the decrease of total Hif1α protein levels during reoxygenation (Figure 8B). Moreover, HSPA12A overexpression increased Hif1α nuclear localization in NRCM upon H/R challenge (Figure 8C). Similar results were observed in murine hearts subjected to MI/R; the MI/R-induced decrease of Hif1α protein levels in nuclear fractions was further exacerbated in *Hspa12a^–/–^* mice relative to WT controls (Supplemental Figure 12, A and B).

#### Hif1α mediated the HSPA12A-maintenaned aerobic glycolysis.

To determine if Hif1α mediates the maintenance of aerobic glycolysis by HSPA12A, we treated NRCM with the Hif1α inhibitor YC-1 during reoxygenation as described previously (35, 36). YC-1 reversed the HSPA12A-induced increase of total Hif1α protein levels in H/R NRCM (Figure 8D). Notably, the HSPA12A-induced increase of extracellular lactate of H/R NRCM was also reversed by YC-1 (Figure 8E)*,* suggesting that inhibition of Hif1α disrupted the HSPA12A-maintained glycolytic flux during reoxygenation. In supporting this, Hif1α inhibition with YC-1 abolished HSPA12A-induced upregulation of glycolysis-related genes (GLUT1, HK-II, and LDHA) in H/R NRCM (Figure 8D). Furthermore, YC-1 abolished HSPA12A-induced attenuation of LDH release in H/R NRCM (Figure 8F). Collectively, these findings demonstrate that HSPA12A maintained aerobic glycolytic homeostasis to protect cardiomyocytes against H/R challenge in a Hif1α-dependent manner.

### HSPA12A increased Hif1α protein levels by promoting Smurf1-mediated Hif1α stabilization in H/R cardiomyocytes

#### HSPA12A increased Hif1α protein stabilization in H/R-treated cardiomyocytes.

**Figure 9:**
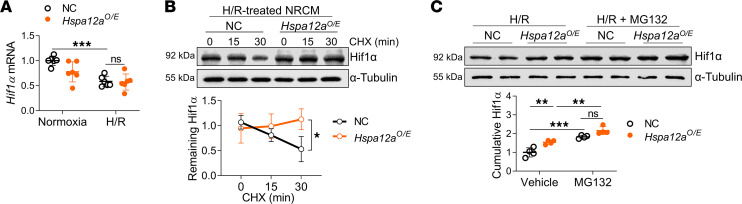
HSPA12A increased Hif1α protein stability in H/R cardiomyocytes. (**A**) *Hif1α* mRNA levels were examined in NRCM. *n* = 6/group. (**B**) After CHX treatment during reoxygenation, the remaining Hif1α protein levels in NRCM were examined by immunoblotting. *n* = 4/group. (**C**) After MG132 treatment during reoxygenation, the cumulative Hif1α protein levels in H/R NRCM were examined by immunoblotting. *n* = 4/group. Data are shown as mean ± SD. ***P < 0.001, **P < 0.01, and *P < 0.05 by 2-way ANOVA followed by post hoc test.

Finally, we determined how HSPA12A increases Hif1α protein levels in H/R-treated NRCM. PCR analysis revealed no difference of *Hif1α* mRNA levels between the 2 H/R groups (Figure 9A), suggesting that HSPA12A could increase Hif1α protein levels by controlling its protein stability. To test this hypothesis, NRCM were treated with cycloheximide (CHX) during the reoxygenation period to block de novo protein synthesis. Notably, CHX treatment for 30 minutes during reoxygenation significantly decreased Hif1α protein levels in the NC group but not the *Hspa12a^O/E^* group (Figure 9B), suggesting that HSPA12A increased Hif1α protein stability in cardiomyocytes during reoxygenation.

#### Smurf1 regulated HSPA12A-induced increase of Hif1α protein stabilization.

The proteasome is an important system for controlling protein stability via protein degradation (4, 37). To determine if HSPA12A stabilizes Hif1α protein by modulating proteasomal degradation, H/R NRCM were treated with the proteasome inhibitor MG132 for 3 hours during the reoxygenation period. MG132 increased cumulative Hif1α protein levels in both the NC and *Hspa12a^O/E^* groups (Figure 9C). However, the Hif1α protein levels did not differ between the NC and *Hspa12a^O/E^* groups in the presence of MG132 (Figure 9C). These findings suggest that HSPA12A increased Hif1α protein stability by inhibiting proteasomal degradation.

**Figure 10:**
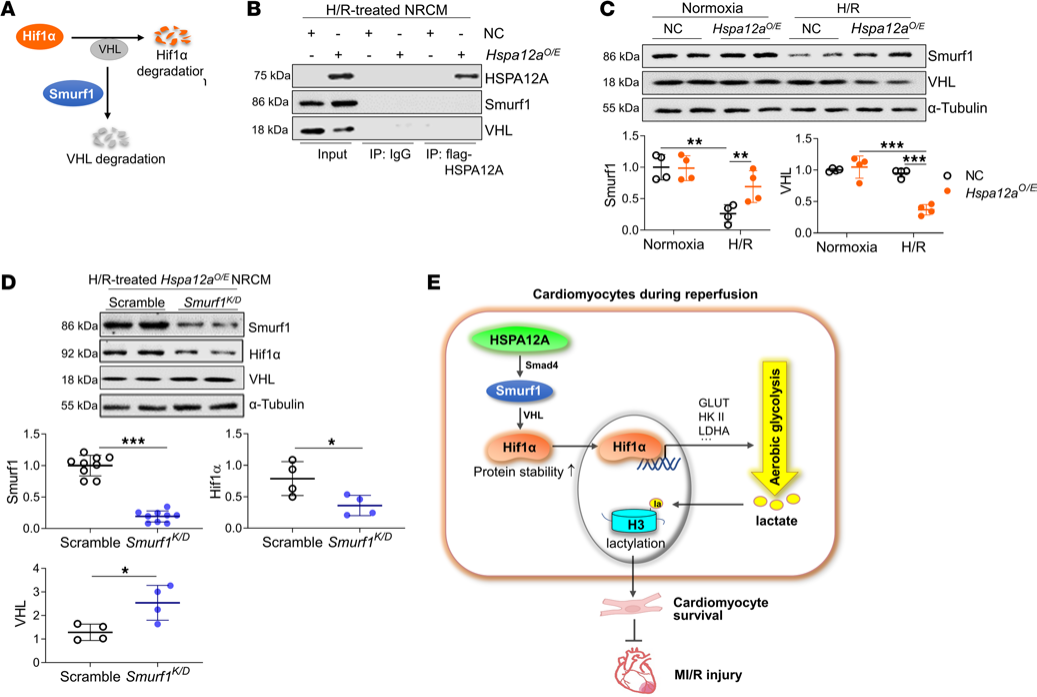
HSPA12A increased Hif1α protein stability of H/R cardiomyocytes in a Smurf1-dependent manner. (**A**) Scheme illustrated that Smurf1 can indirectly increase Hif1α protein stability. (**B**) IP immunoblotting analysis. Flag-tagged HSPA12A immunoprecipitates were prepared from H/R NRCM and subsequently subjected to immunoblotting against HSPA12A, Smurf1, and VHL. Input served as positive controls, and IgG immunoprecipitates served as negative controls. (**C**) Protein expression of the indicated genes was examined by immunoblotting in NRCM. *n* = 4/group. (**D**) Effects of Smurf1 knockdown on Hif1α and VHL expression in H/R-treated *Hspa12a^O/E^* NRCM was examined using immunoblotting. *n* = 9/group for Smurf1 analysis, and *n* = 4/group for analysis of Hif1α and VHL. (**E**) Mechanistic scheme. HSPA12A protects against MI/R injury through maintaining aerobic glycolytic homeostasis and Histone3 (H3) lactylation in cardiomyocytes by increasing Smurf1-mediated Hif1α protein stabilization. Data are shown as mean ± SD. ***P < 0.001, **P < 0.01, and *P<0.05 by 2-way ANOVA followed by post hoc test (**C**) or Student’s 2-tailed unpaired *t* test (**D**).

Hif1α is classically targeted for proteasomal degradation by Von Hippel-Lindau ligase (VHL) (38). Bioinformatics analysis (http://ubibrowser.ncpsb.org/) revealed that VHL was not among the top 19 of ubiquitin ligases/deubiquitinases with potential HSPA12A binding, while Smurf1 was the most likely binding partner (Supplemental Table 1). Considering that Smurf1 can promote VHL proteasomal degradation (Figure 10A) (39), we performed IP immunoblotting and found that neither Smurf nor VHL were present in anti–flag-tagged HSPA12A immunoprecipitates prepared from H/R NRCM (Figure 10B). However, HSPA12A overexpression attenuated H/R-induced Smurf1 downregulation in NRCM (Figure 10C). Moreover, *Hspa12a^O/E^* NRCM exhibited decreased VHL levels relative to WT NRCM after H/R (Figure 10C). Similarly, in mouse hearts, MI/R-induced downregulation of Smurf1 was further exacerbated in *Hspa12a^–/–^* hearts relative to WT controls (Supplemental Figure 12C).

To further determine if Smurf1 mediates the HSPA12A-induced increase of Hif1α protein stability, we knocked down Smurf1 in *Hspa12a^O/E^* NRCM (Figure 10D). Under normoxic conditions, Smurf1 knockdown did not change Hif1α protein levels in *Hspa12a^O/E^* NRCM (Supplemental Figure 13A). However, Smurf1 knockdown not only increased VHL levels but also decreased Hif1α protein levels in *Hspa12a^O/E^* NRCM following H/R challenge (Figure 10D). We also noticed that Smurf1 knockdown abolished the increase of Bcl-2 protein expression in H/R-treated NRCMs (Supplemental Figure 13B). These findings suggest that Smurf1 mediated the HSPA12A-induced increase of Hif1α protein stability.

#### HSPA12A upregulated Smurf1 expression in a Smad4-dependent manner.

To elucidate the mechanism by which HSPA12A regulates Smurf1 expression, *Smurf1* mRNA levels were measured using real-time PCR analysis, revealing that H/R-induced downregulation of *Smurf1* mRNA levels were attenuated in *Hspa12a^O/E^* NRCM relative to NC (Supplemental Figure 14A). This suggested that HSPA12A upregulated Smurf1 expression at the transcriptional level in cardiomyocytes following H/R challenge.

To identify the mechanism by which HSPA12A upregulates Smurf1 transcription, we performed bioinformatic analyses to predict the putative transcription factors for *Smurf1* expression using the PROMO database (https://alggen.lsi.upc.es/cgi-bin/promo_v3/promo/promoinit.cgi?dirDB=TF_8.3), Animal TFDB (http://bioinfo.life.hust.edu.cn/AnimalTFDB4/#/), and JASPAR database (https://jaspar.elixir.no/). Seven transcription factors were predicted to bind the *Smurf1* promoter, with the highest score predicted for Smad4 (Supplemental Figure 14B). To determine if Smad4 mediates HSPA12A-induced Smurf1 upregulation, we first examined the effects of HSPA12A on Smad4 expression. H/R decreased Smad4 protein levels in NRCM, a decrease that was attenuated in *Hspa12a^O/E^* NRCM relative to NC (Supplemental Figure 14C). Furthermore, nuclear localization of Smad4 was increased in *Hspa12a^O/E^* NRCM relative to NC following H/R challenge (Supplemental Figure 14D). Notably, when knockdown of Smad4 decreased nuclear Smad4 levels in H/R-treated *Hspa12a^O/E^* cardiomyocytes (Supplemental Figure 15A), Smad4 knockdown also abolished the increases of Smurf1 and Hif1α protein levels in H/R-treated *Hspa12a^O/E^* cardiomyocytes relative to NC (Supplemental Figure 15B). Furthermore, Smad4 knockdown abolished increased Bcl-2 levels in H/R-treated *Hspa12a^O/E^* cardiomyocytes relative to H/R-treated NC cardiomyocytes (Supplemental Figure 15C). Collectively, the findings suggest that HSPA12A activated Smad4 to increase Smurf1 expression in cardiomyocytes upon H/R challenge.

## Discussion

The central findings of the present study are that aerobic glycolysis and H3 lactylation were inhibited and HSPA12A expression was downregulated in cardiomyocytes during reperfusion following ischemia, and that HSPA12A ablation exacerbated MI/R-induced cardiac dysfunction and remodeling. Moreover, HSPA12A supported cardiomyocyte survival upon H/R challenge by maintaining aerobic glycolysis and subsequent H3 lactylation by increasing Smurf1-mediated Hif1α protein stability (Figure 10E).

Myocardial I/R injury, a common clinical phenomenon that occurs during reperfusion therapy for acute MI, is characterized by additional injury to the ischemic heart, including expansion of the infarct size and arrhythmia. Appropriate clinical management of MI/R injury is, thus, crucial to improve clinical outcomes of MI/R. Previous studies have demonstrated that some heat shock proteins (HSPs) confer cardioprotection against MI/R injury (26, 40–42). HSPs are an evolutionarily conserved superfamily comprising a group of structurally unrelated subfamilies, including HSPA/HSP70, HSPB/HSP27, HSPC/HSP90, HSPH/HSP110, and NDAJ/HSP40 (43). HSP70, HSP27, HSP20, and HSPA12B have been reported to attenuate MI/R injury in experimental studies (26, 40–42). However, clinical targeting of these HSPs has been unsuccessful, warranting further studies to identify novel HSPs involved in MI/R pathogenesis. In the present study, we identified that HSPA12A, a distant and atypical member of the HSP70 family, was downregulated in cardiomyocytes in response to MI/R, suggesting its possible involvement in MI/R injury. Indeed, we identified that MI/R-induced infarct size was increased and cardiac dysfunction was exacerbated in HSPA12A-KO mice. Moreover, loss- and gain-of-function in vitro studies demonstrated an essential role for HSPA12A in cardiomyocyte survival following H/R challenge. Taken together, these findings strongly suggest that HSPA12A has cardioprotective effects in the context of MI/R injury.

The heart is energetically demanding, requiring abundant ATP to support continual systolic and diastolic function, and its utilization of energy substrates is highly flexible. Under physiological conditions, the majority of ATP is produced by fatty acid β-oxidation, and the remainder is produced by glucose oxidation and glycolysis (7, 9, 44). However, under pathological conditions such as MI/R, the substrates for ATP generation change dramatically. Due primarily to mitochondrial dysfunction and other energetic impairments, the heart is forced to reprogram its fatty acid–based metabolism to aerobic glycolysis, which can protect the heart by providing continued ATP production, preventing opening of the mitochondrial permeability transition pore, and decreasing ROS production (9–12). We found that, when HSPA12A was downregulated in cardiomyocytes during the reoxygenation period following hypoxia, lactate generation and glycolysis-related gene expression were also decreased. Interestingly, HSPA12A overexpression maintained cardiomyocyte aerobic glycolytic homeostasis during the reoxygenation period following hypoxia. Remarkably, inhibiting aerobic glycolysis with 2-DG or Oxa during reperfusion abolished the cardioprotective effects of HSPA12A against H/R challenge. Collectively, these findings demonstrate that HSPA12A confers cardioprotection against MI/R injury by maintaining aerobic glycolytic homeostasis.

Glycolysis plays crucial roles in myriad physical and pathological processes, but the underlying mechanisms by which glycolysis regulates these processes, in addition to its role in energy metabolism, remain incompletely understood. Intriguingly, recent studies demonstrate that glycolysis could regulate some biological processes via protein lactylation. Lactylation, a posttranslational modification identified in 2019, is the process by which p300 catalyzes binding of lactyl groups derived from glycolysis to the lysine residues of target proteins. Lactylation can occur in both Histones and non-Histone proteins (15). Histone is the first identified protein that can be lactylated in cancer cells, and later studies demonstrate that Histone lactylation plays critical roles in cancer progression and macrophage inflammation response (15, 31). Histone lactylation is an epigenetic marker of the glycolytic switch (17). Moreover, lactylation of Snail1 mediates the endothelial-to-esenchymal transition after MI (45), while α-MHC lactylation maintains the α-MHC–Titin interaction to alleviate the development of heart failure (18). We also recently demonstrated that HMGB1 lactylation mediates macrophage activation to exacerbate liver I/R injury (25). However, the role of Histone lactylation in MI/R injury is poorly known. In the present study, we report that H3K56la was decreased in cardiomyocytes following H/R and that the decrease was prevented by HSPA12A overexpression. Furthermore, inhibition of H3K56 lactylation either using Oxa to block glycolytic lactate generation or using a p300 inhibitor (C646) to inhibit lactyl group transfer abolished the protective effects of HSPA12A against H/R-triggered cardiomyocyte death. Collectively, our findings indicate that maintenance of H3K56 lactylation is essential for cardiomyocyte survival upon H/R challenge and that HSPA12A maintains H3K56 lactylation in this context.

Glycolysis, the metabolic process that converts glucose to pyruvate and subsequently lactate, is perpetuated by a set of related genes for glucose uptake (GLUT1 and GLUT4), glycolytic catalysis (HK-II, PKM2, and LDHA), and lactate export (MCT4 and CD147). Hif1α is an important transcription factor that regulates expression of these genes (3, 46–48). Although prior studies demonstrate that Hif1α mediates cardioprotective effects of SUMO-specific protease 1 against MI/R injury (49), it is unclear whether its cardioprotective effects are due to support of aerobic glycolysis. In the present study, Hif1α protein levels were decreased in MI/R hearts and H/R cardiomyocytes. This decrease was attenuated by HSPA12A overexpression and further exacerbated by HSPA12A knockdown or genetic deletion. Importantly, pharmacological inhibition of Hif1α reversed the HSPA12A improvement of both aerobic glycolysis and cardiomyocyte survival following H/R challenge. These findings suggest that HSPA12A protects cardiomyocytes against H/R by maintaining Hif1α-mediated aerobic glycolysis.

Although we demonstrate that HSPA12A increased Hif1α protein levels in cardiomyocytes following H/R challenge, HSPA12A did not affect *Hif1α* mRNA levels. This suggests that HSPA12A could regulate Hif1α protein levels at the posttranslational level. Indeed, HSPA12A increased Hif1α protein stability, as indicated by blocking de novo protein synthesis studies with CHX and proteasomal inhibition with MG132. Hif1α is well known to be rapidly degraded via the proteasomal pathway after targeting by VHL, which functions as the recognition component of an E3-ubiquitin ligase. Downregulation of VHL results in Hif1α stabilization and increased Hif1α transcriptional activity (38). The E3 ligase Smurf1 can bind VHL and promote its ubiquitination and degradation, thus serving as a ubiquitin ligase link between VHL and Hif1α (39). Though bioinformatics predicted that Smurf1 was the most likely ubiquitin ligase binding partner of HSPA12A binding, HSPA12A did not directly interact with Smurf1 or VHL. However, HSPA12A attenuated H/R-induced Smurf1 downregulation in cardiomyocytes, while Smurf1 knockdown decreased Hif1α protein expression and increased VHL protein expression in *Hspa12a^O/E^* NRCM subjected to H/R. Also, HSPA12A upregulated Bcl-2 expression in a Smurf1-dependent manner. Further experiments demonstrated that HSPA12A upregulated Smurf1 expression at the transcriptional level in a Smad4-depended manner. Taken together, our findings suggest that HSPA12A increases Hif1α protein stability in a Smurf1-dependent manner.

In conclusion, we demonstrate that HSPA12A supports cardiomyocyte survival to attenuate MI/R injury. This cardioprotective effect of HSPA12A is mediated, at least in part, by maintaining aerobic glycolytic homeostasis and subsequent H3 lactylation via increasing Smurf1-mediated Hif1α protein stability. This suggests that targeting cardiomyocyte HSPA12A is a potential therapeutic approach for myocardial I/R injury.

## Methods

### Sex as a biological variable

Male mice were used in the experiment because premenopausal females have a reduced risk of coronary artery disease and experience decreased long-term morbidity after percutaneous coronary intervention (PCI) (50–52). Mice (C57BL/6 background, male) were randomly assigned to all analyses.

### Reagents

Dulbecco’s modified eagle’s medium (DMEM) and FBS were from Thermo Fisher Scientific. High-sensitivity enhanced chemiluminescence (high-sig ECL) Western blotting substrate was from Tanon. DAPI reagent was from Cell Signaling Technology. Lactate assay kit and LDH assay kit were from Jiancheng Biotech. PAS staining kit was from Beyotime. Glycogen assay kit was from Solarbio. Seahorse XF Glycolysis Stress Test Kit and Mitochondrial Stress Test Kit were from Aglient. The TUNEL assay kit was from Promega. 2-DG, YC-1, CHX, MG132, C646, and propidium iodide (PI) reagents were from MedChemExpress. Sodium Oxa was from Selleckchem. Live/dead viability/cytotoxicity kit was from Thermo Fisher Scientific. Collagenase Type II was from Worthington Biochemical Corporation. Triphenyltetrazolium chloride (TTC) and pancreatin were from Sigma-Aldrich. The antibodies used in this study are listed in Supplemental Table 2.

### Animals

#### Generating Hspa12a^–/–^ mice.

*Hspa12a^–/–^* mice with C57BL/6 genetic background were generated using the *lox*P/*Cre* recombinant system as we described previously (22, 23). In brief, the region of the *Hspa12a* gene containing exons 2–4 was retrieved from a 129/sv BAC clone (BAC/PAC Resources Center) using a retrieval vector containing 2 homologous arms. Exons 2 and 3 were replaced by loxP sites flanking a PGK-neo cassette as a positive selection marker. Embryonic stem cells were electroporated with the linearized targeting vector, selected, and then expanded for Southern blot analysis. Chimeric mice were generated by injecting embryonic stem cells into C57BL/6 blastocysts, followed by transfer into pseudopregnant mice. To remove the *Hspa12a* gene, the conditional *Hspa12a* mice were crossed with EIIa-Cre transgenic mice. The deletion of HSPA12A was confirmed by immunoblotting.

#### Generating cardiomyocyte-specific Hspa12a^ki^ mice.

The conditional *Hspa12a*-knockin mice were recently created by our team using CRISPR/Cas9 system (25). Briefly, 1 sgRNA targeting the near sequence of inserted site constructed and transcribed in vitro, and the donor vector with the inserted fragment was designed and constructed in vitro. Then, Cas9 mRNA, sgRNA, and the donor were coinjected into zygotes followed by transplanting of these zygotes into the oviduct of pseudopregnant ICR females at 0.5 days postcoitum (dpc). After 19–21 days of transplantation, F0 mice were born, identified by PCR and tail DNA sequencing, and crossed with WT mice to build up heterozygous conditional *Hspa12a*-knockin mice. To overexpress *Hspa12a* gene in cardiomyocytes, the conditional *Hspa12a*-knockin mice were crossed with *Myh6-Cre* transgenic mice. The breading strategy was shown in Supplemental Figure 4A. The cardiomyocyte-specific HSPA12A overexpression was confirmed by immunoblotting.

Mice were bred at the Model Animal Research Center of Nanjing University and maintained on a 12-hour light/dark cycle at 23°C ± 1°C with access to food and water ad libitum. Investigators were blinded to the histological and echocardiographic analysis. Investigators involved in animal handling and sampling were not blinded.

### MI/R

MI/R was induced in male, 8- to 12–week-old *Hspa12a^–/–^* mice and WT littermates according to our previous methods (23, 53). Briefly, mice were anesthetized with inhalation of 1.5%–2% isoflurane, and the adequacy of anesthesia was evaluated by the disappearance of righting reflex and pedal withdrawal reflex. The left anterior descending (LAD) coronary artery was ligated to induce ischemia for 45 minutes, and then the coronary artery was reperfused by releasing the ligation. In sham-operated animals, the same surgical procedure was performed except the LAD ligation. Analgesia was conducted according to our previous studies (53). For tissue collection, mice were sacrificed by overdose anesthesia (pentobarbital sodium 150 mg/kg i.p. injection) and cervical dislocation.

### Infarct size

At the end of reperfusion, the LAD was reoccluded, and the heart was perfused with 2% Evans blue (26, 54). The hearts were then rapidly excised, sectioned into 1 mm slices after being flash frozen in liquid nitrogen, and were stained with TTC. Blue zones stained with Evans blue were nonischemic areas, whereas Evans blue nonstained zones were ischemic areas at risk, including the red and white zones stained with TTC. The white zones with TTC staining were infarct areas. The MI size was calculated as the ratio of infarct area to the ischemic area at risk.

### Cardiac function

Cardiac function was measured by echocardiography in mice at the end of reperfusion for 3 hours and 7 days using the Vevo770 system equipped with a 35 MHz transducer (Visualsonics) according to our previous methods (26, 53). The measurements were performed by an observer blinded to the treatment. Cardiac functional parameters were obtained from M-mode tracings and averaged using 3–5 cardiac cycles.

### Isolation and culture of primary NRCM

NRCM were isolated from heart of neonatal Sprague-Dawley rat (1–3 days old) according to our previous methods (53). Briefly, after being anesthetized with 2% isoflurane and decapitated, ventricular tissues were collected, minced, and digested with type II collagenase (0.4 mg/mL) and pancreatin (0.6 mg/mL). The released cells were preplated for 1 hour, the attached cells were considered cardiac fibroblasts, and the unattached cells (cardiomyocytes) were collected for plating on other dishes. After preplating, cardiomyocytes were resuspended in DMEM with 10% FBS and 0.1% cytosine arabinoside (Ara-c) and plated in 6-well plates (5 × 10^5^/well) or on coverslips in 24-well plates (1 × 10^5^/well). All the experiments were grouped randomly.

### Overexpression and knockdown of interest genes in cardiomyocytes

To overexpress of HSPA12A, NRCM were infected with flag-tagged HSPA12A-expressing adenovirus (*Hspa12a^O/E^*), and the NRCM infected with empty adenovirus served as NC. To knock down HSPA12A, Smurf1, or Smad4, NRCM were transfected with the siRNA targeting HSPA12A, Smurf1, or Smad4 using siRNA-mate (Genepharma), and NRCM transfected with scramble RNA served as NC. The siRNA sequences are listed in Supplemental Table 3.

### H/R

To simulate MI/R in vitro, NRCM were subjected to H/R according to our previous methods with modification (53). Briefly, NRCM were exposed to hypoxia (94% N_2_, 5% CO_2_, and 1% O_2_, 37°C) in the medium derived with serum and glucose. After hypoxia for 6 hours, NRCM were changed to reoxygenation condition (95% air and 5% CO_2_, 37°C) in normal growth medium.

In glycolysis inhibition experiments, NRCM were treated with 2-DG (5 mM) or Oxa (5 mM) during reoxygenation after hypoxia. For Hif1α inhibition experiments, YC-1 (10 μM) was introduced to NRCM culture during reoxygenation. For p300 inhibition, C646 (15 μM) was introduced to NRCM culture during reoxygenation period.

### NRCM injury measurement

#### Morphology.

NRCM morphology was examined under a phase-contrast light microscope (Zeiss Ltd.). Three fields on each well were randomly examined.

#### Live/dead assay.

Death of NRCM was indicated by a live/dead viability/cytotoxicity analysis according to our previous methods (55), which provides 2-color fluorescence based on living (green) and dead (red) cells. The staining was observed under a fluorescence microscope (Zeiss). More than 3 images per culture well were taken at random. Cell death was expressed as a percentage of dead cells to total cells.

#### PI uptake.

PI dye uptake assay is an indicator of dying or dead cells (56). After stained with PI (5 μM) for 20 minutes, DAPI was counterstained to indicate nuclei. The staining was observed under a fluorescence microscope. More than 3 images per culture well were taken at random. Cell death was expressed as a percentage of dead cells to total cells.

#### LDH release.

The leaked LDH was expressed as the percentage of activity in medium divided by total activity in cells according to our previous methods (57). Total LDH activity = (LDH activity in the medium) + (LDH activity in the lysate of cells treated with 0.2% Tween 20).

#### Beating frequency.

The beating of NRCM was recorded for 30 seconds by a camera and counted by a stopwatch online clock according to previous study (53). Three areas were randomly selected in each sample. Image taking and beat counting were conducted by an investigator blinded to the treatment of experiments.

### Cardiomyocyte apoptosis in hearts of mice

Heart tissues at the papillary muscle level were harvested for paraffin sectioning after I/R. Apoptosis was evaluated using the TUNEL assay. Cardiomyocytes were indicated by α-actinin immunostaining. Nuclei were counterstained by DAPI. The staining was observed and quantified using Cellsens Dimention 1.15 software (Olympus). More than 4 fields in the infarct areas on each slide were randomly examined.

### Cardiac glycogen content in hearts of mice

#### PAS staining.

Heart tissues at the papillary muscle level were harvested for paraffin sectioning according to previous studies (58). PAS staining was performed on the sections using the PAS staining kit according to the manufacturer’s instructions. The staining was observed and quantified using Cellsens Dimention 1.15 software (Olympus). More than 4 fields in the infarct areas on each slide were randomly examined.

#### Glycogen content.

Cardiac glycogen content was quantified utilizing a glycogen content assay kit following the manufacturer’s instructions. The glycogen content was normalized in micrograms of glycogen per gram of tissue.

### Extracellular lactate content

Following H/R, medium of NRCM culture was harvested for lactate content analysis using the assay kit according to the manufacture’s instruction. The lactate values were expressed as relative contents to the respective NC controls.

### Cardiomyocyte glucose uptake

Glucose uptake was examined using glucose analog 2-NBDG according to previous study (53, 59). Following H/R, NRCM culture was incubated with 2- NBDG (200 μM) at 37°C for 30 minutes and then washed 3 times with ice-cold PBS. The content of 2-NBDG was measured using fluorescence plate reader (BioTek Synergy) at an excitation wave length of 485 nm and emission wave length of 528nm.

### 18F-FDG PET Imaging

The PET/CT was used to evaluate heart glucose uptake after I/R according to previous studies (27, 60). PET/CT scanning was performed 3 hours after I/R surgery on a small-animal PET/CT scanner (Inveon MM-PET/CT, Siemens Medical Solutions). Mice were injected i.v. with a dose of approximately 7.4 MBq (200 μCi) ^18^F-FDG via tail vein. After an uptake period of 60 minutes, mice were anesthetized with 2% isoflurane and positioned prone on the imaging bed, with the heart centered in the field of view. Temperature was maintained at 37°C by circulating airflow within the imaging bed chamber. Subsequently, mice were subjected to PET scanning for a total duration of 10 minutes. Whole-body CT scans were also performed for attenuation correction and anatomical reference. The matrix, thickness, and energy window of PET scanning were 128 × 128 × 159, 0.796 mm, and 350–650 keV, respectively. The matrix, voltage, current, and spatial resolution of CT scanning were 512 × 512 × 1008, 80 kV, 500 μA, and 110.3 μm, respectively. Image analyses were performed using an Inveon Research Workplace software (Siemens) according to previous study (60, 61). Images were processed using an OSEM3D algorithm (2 OSEM iterations, 18 MAP iterations, and 1.5 target resolution). Regions of interest (ROI) were drawn manually at the center of the I/R area in the apical anterolateral wall. The %ID/g was analyzed and quantitated to evaluate the ^18^F-FDG uptake activity.

### Seahorse glycolytic flux and glucose oxidation

Glycolytic flux and glucose oxidation were completed on isolated NRCM by measuring ECAR and mitochondrial OCR using a Seahorse XF24 flux analyzer (Seahorse Bioscience). Experiments were performed following manufacturer protocols. In brief, 5 × 10^4^ cells per well were seeded into a Seahorse XFe24 cell culture microplate with fresh growth medium after HSPA12A over expression for 24 hours following H/R. Subsequently, the growth medium was changed to the assay medium. For OCR, the assay medium was prepared with DMEM base medium supplemented with 1 mM pyruvate, 2 mM glutamine, and 10 mM glucose (Agilent). For ECAR, the assay medium consisted of DMEM base medium with 2 mM glutamine (Agilent). After being incubated for 1 hour in a CO_2_ free 37°C incubator, the basal respiration of the NRCM was measured. For the glycolysis stress test, glucose (10 mM), oligomycin (1 μM), and 2-DG (50 mM) were injected (Agilent). For the mitochondrial stress test, oligomycin (1.5 μM), FCCP (2.5 μM), rotenone (0.5 μM), and antimycin A (0.5 μM) were injected (Agilent) (62). Each experimental treatment was performed on 2 wells of each plate as technical replicates, and each experiment included 3 biological replicates. The ECAR and OCR were normalization by cell number in each well.

### Immunoblotting and IP immunoblotting

For immunoblotting, myocardial infarcts or NRCM were harvested for protein preparation. Equal amounts (20 μg) of proteins were used for immunoblotting according to our previous methods (22, 23). To control for lane loading, the membranes were probed with anti–α-tubulin antibody (cytosolic fractions) or anti-Histone3 (nuclear fractions) (Supplemental Table 2). The developed bands were normalized to controls and expressed as the relative levels.

For analyzing interactions between HSPA12A and Hif1α, Smurf1, or VHL by IP immunoblotting, NRCM were overexpressed with flag-tagged HSPA12A. Equal amounts (0.7 mg) of proteins, which were prepared from H/R NRCM, were precipitated with anti-flag antibodies, followed by immunoblotting for HSPA12A, Hif1α, Smurf1, and VHL, as described previously (23).

### Immunofluorescence staining

Immunofluorescence staining was performed on 4% PFA-fixed NRCM according to our previous method (24, 53). Briefly, after incubation with the primary antibodies (1:100) overnight at 4°C, Cy3-conjugated secondary antibody was applied to the samples to visualize the staining. DAPI was used to counterstain the nuclei. The staining was observed using a fluorescence microscope and quantified using Cellsens Dimention 1.15 software (Olympus).

### Quantitative PCR

Quantitative PCR (qPCR) was performed to evaluate *Hif1α* mRNA levels as described previously (24). Briefly, total RNA was extracted from NRCM and 2 μg of total RNA was used for cDNA synthesis using the oligo (dT) primer. After cDNA synthesis, *Hif1*α and *Smurf1* mRNA levels were estimated by real-time PCR using the SYBR Green Master (Roche). The PCR results of *36b4* served as internal controls. The primers used for PCR were listed in Supplemental Table 4**.**

### Protein stability analysis

To investigate whether HSPA12A regulates Hif1α stability by modulating its proteasomal degradation, the de novo protein synthesis inhibitor CHX (20 μM) and proteasome inhibitor MG132 (15 μM) was added to the NRCM culture for the indicated durations. The remaining Hif1α protein was expressed as the percentage to the levels of control cells.

### Statistics

Data are represented as mean ± SD. All data from different groups were verified for normality and homogeneity of variance. For data that are consistent with normality and homogeneity, groups were compared using Student’s 2 tailed unpaired *t* test, 1-way ANOVA, or 2-way ANOVA, followed by the Tukey post hoc test. For the data that are not consistent with normality or homogeneity of variance, Mann-Whitney *U* test and Kruskal-Wallis were used for analyses. *P <* 0.05 was considered statistically significant. Statistical analysis was performed using GraphPad Prism 7.0 (GraphPad Software) and SPSS 23.0 (SPSS Inc.) software.

### Study approval

All animal studies were performed in accordance with European ethical regulation (Directive 2010/63/EU) and approved by the Committee on Animal Care at Nanjing University (no. XG55). All the experiments were conformed to the international guidelines on the ethical use of animals. All experiments conformed to the *Guide for the Care and Use of Laboratory Animals* (National Academies Press, 2011).

### Data availability

Values for all data points in graphs are reported in the Supporting Data Values file.

## Author contributions

LL, ZD, and CL developed the study concept and experimental design; WY, QK, SJ, YL, ZW, QM, XZ, PZ, and QL performed all animal studies and cell experiments; ZD, LL, and YL interpreted the data and wrote the manuscript; and LL involved in study supervision.

## Supplementary Material

Supplemental data

Unedited blot and gel images

Supporting data values

## Version 1 02/29/2024

In-Press Preview

## Version 2 04/08/2024

Electronic publication
