# Supplementary material for: HSPA12A maintains aerobic glycolytic homeostasis and Histone3 lactylation in cardiomyocytes to attenuate myocardial ischemia/reperfusion injury
Source: JCI Insight. 2024 Feb 29;9(7):e169125. doi: 10.1172/jci.insight.169125 (PMC11128201; doi:10.1172/jci.insight.169125)
Supplement: Supplemental data [file jciinsight-9-169125-s251.pdf]

Supplemental Figures

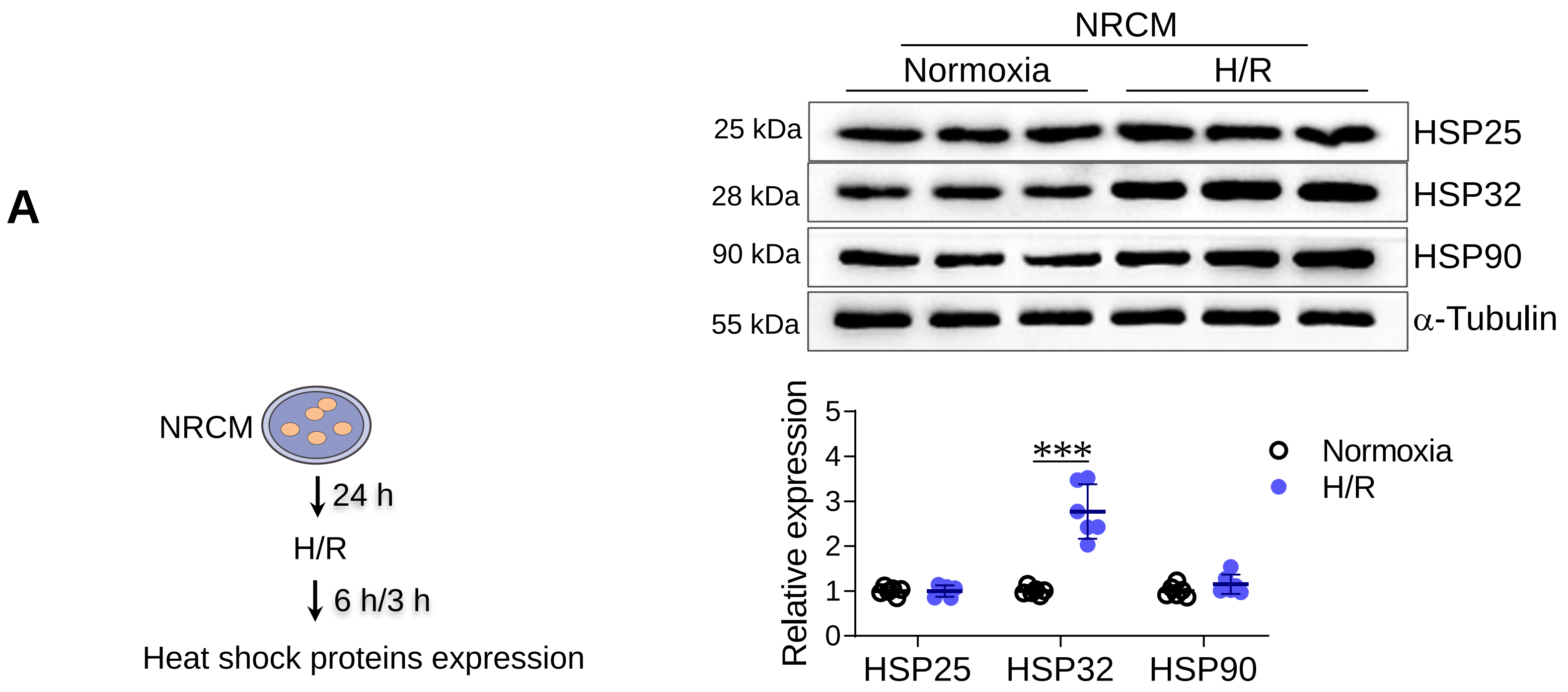

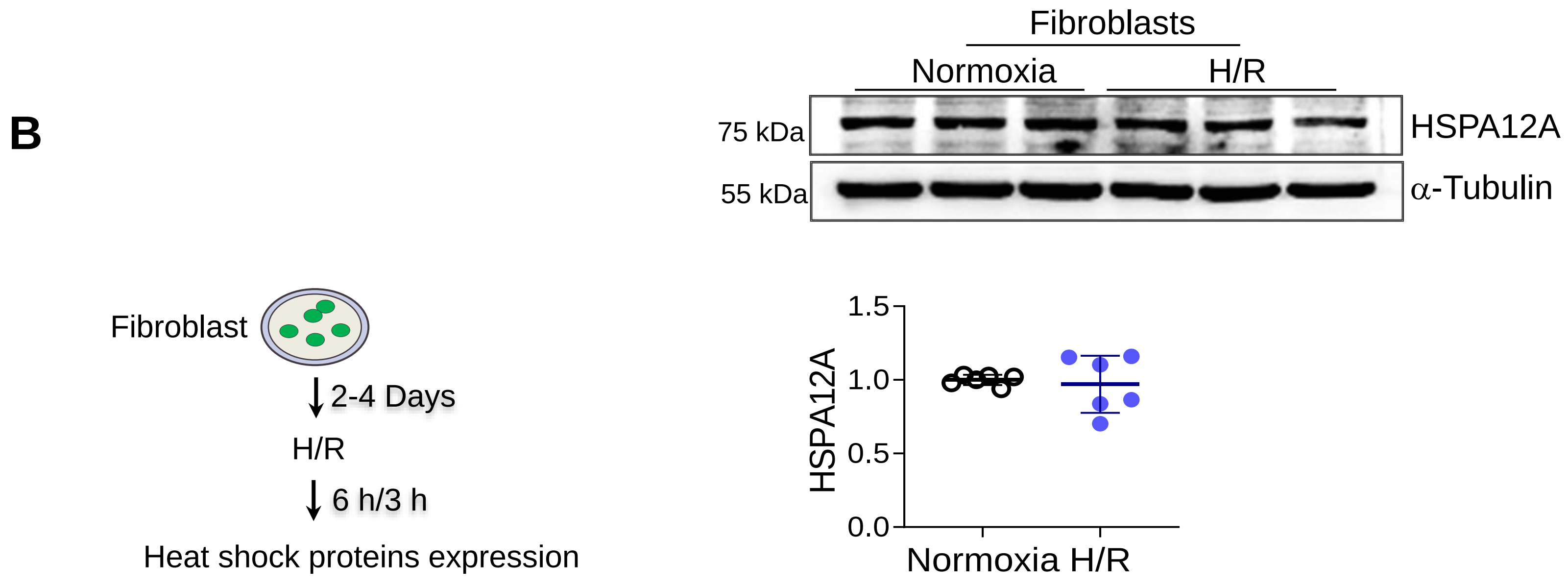

Figure S1

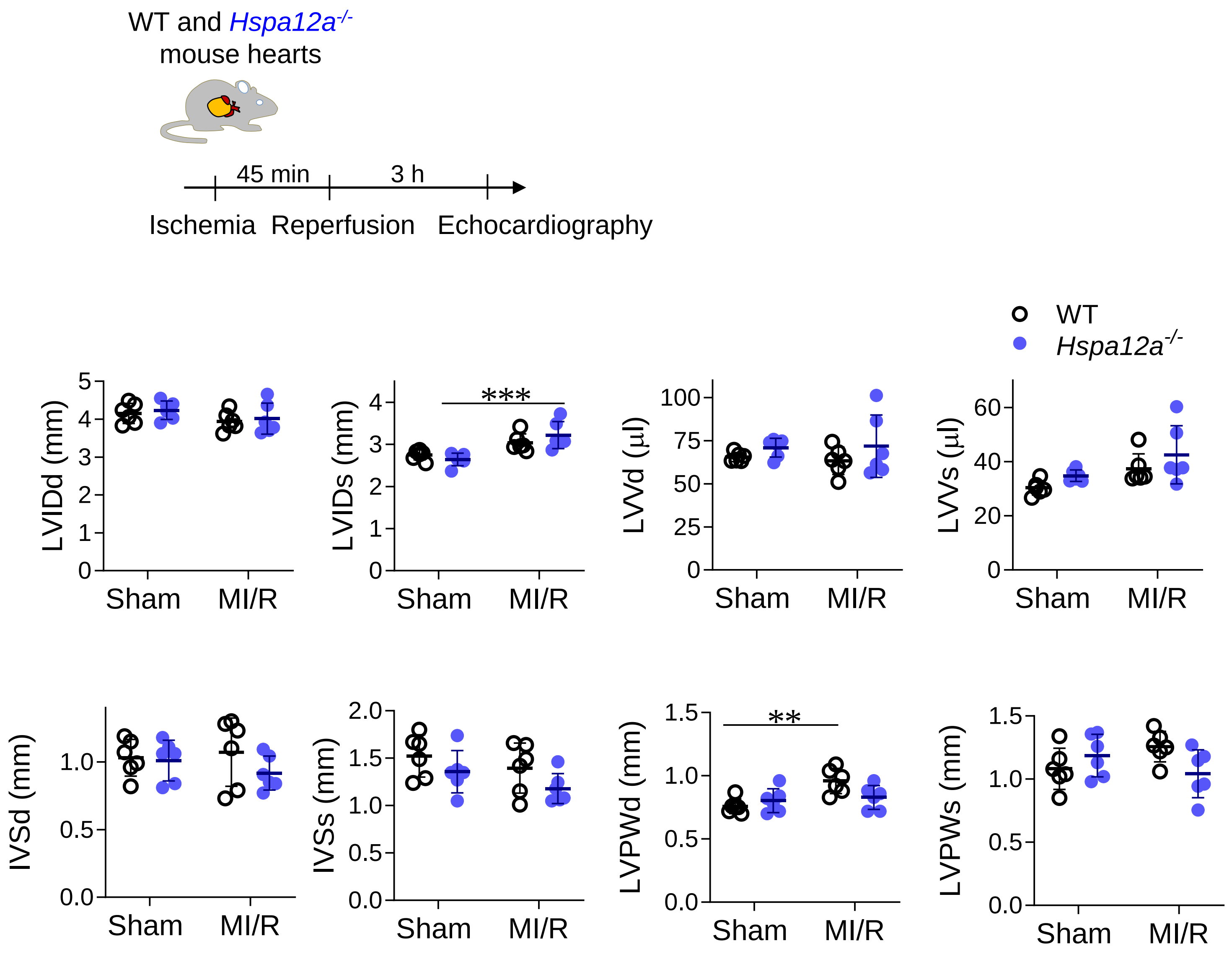
WT and Hspa12a-/-
mouse hearts
45 min
3 h
Ischemia
Reperfusion
Echocardiography
WT
Hspa12a-/-
LVIDd (mm)
LVIDs (mm)
***
LVVd (µl)
LVVs (µl)
IVSd (mm)
IVSs (mm)
LVPWd (mm)
**
LVPWs (mm)
Sham
MI/R

Figure S2

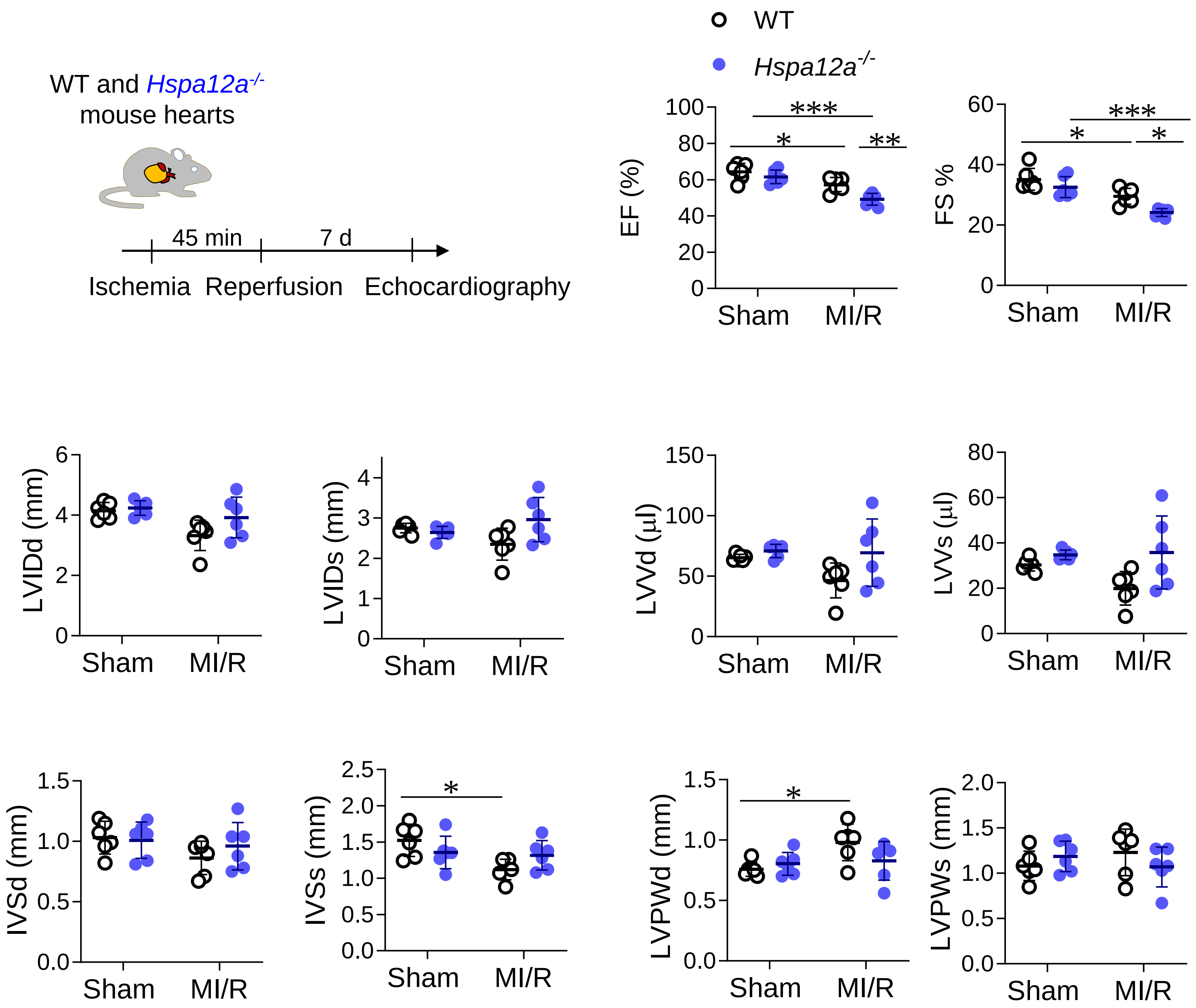
WT and Hspa12a-/-
mouse hearts
45 min
7 d
Ischemia
Reperfusion
Echocardiography
WT
Hspa12a-/-
EF (%)
FS %
LVIDd (mm)
LVIDs (mm)
LVVd (µl)
LVVs (µl)
IVSd (mm)
IVSs (mm)
LVPWd (mm)
LVPWs (mm)
Sham
MI/R

Figure S3

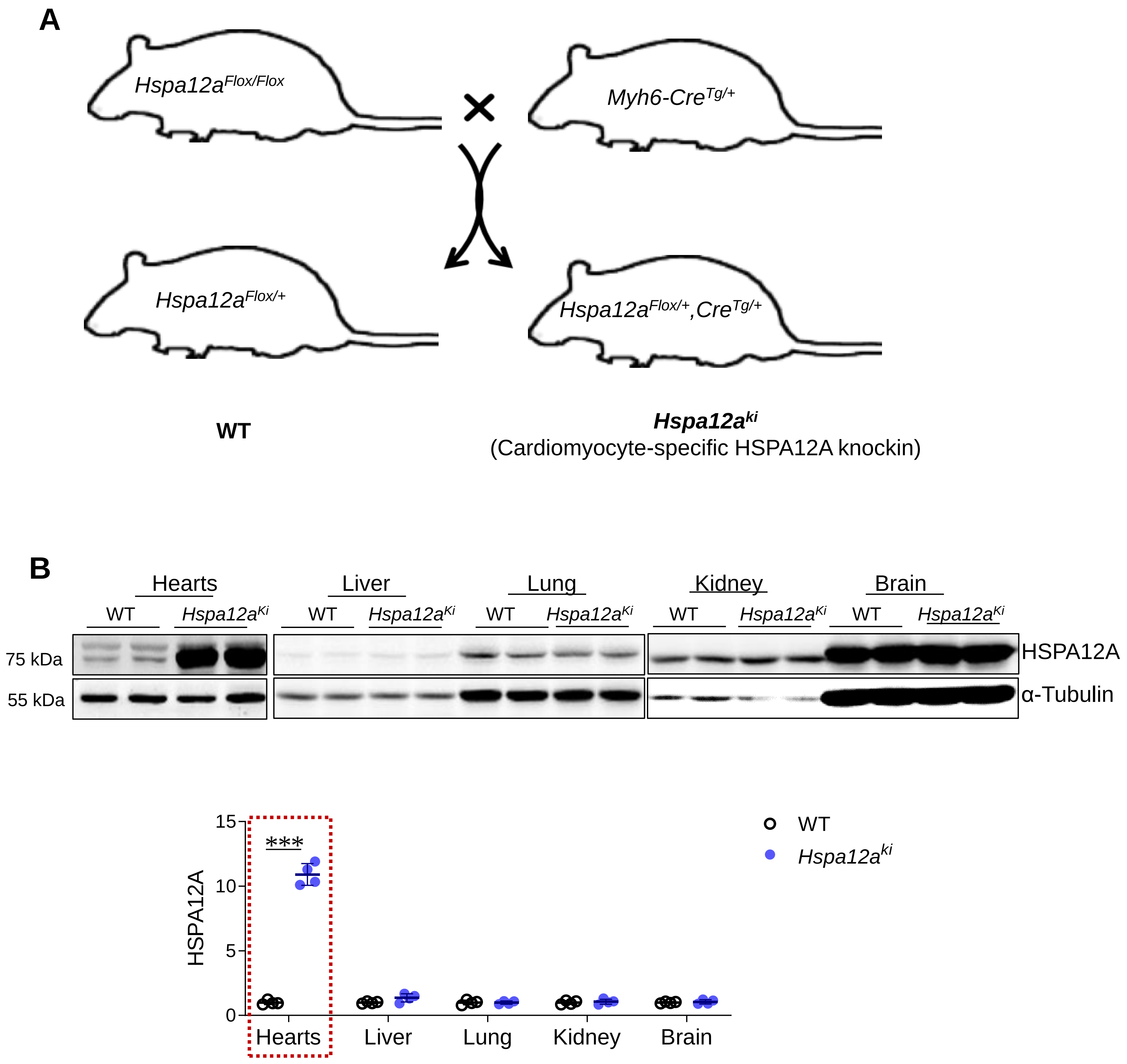
A
Hspa12aFlox/Flox
Myh6-CreTg/+
Hspa12aFlox/+
Hspa12aFlox/+,CreTg/+
WT
Hspa12aki
(Cardiomyocyte-specific HSPA12A knockin)
B
Hearts
Liver
Lung
Kidney
Brain
WT
Hspa12aKi
75 kDa
55 kDa
HSPA12A
α-Tubulin
HSPA12A
15
10
5
0
***
Hearts
Liver
Lung
Kidney
Brain
WT
Hspa12aki

Figure S4

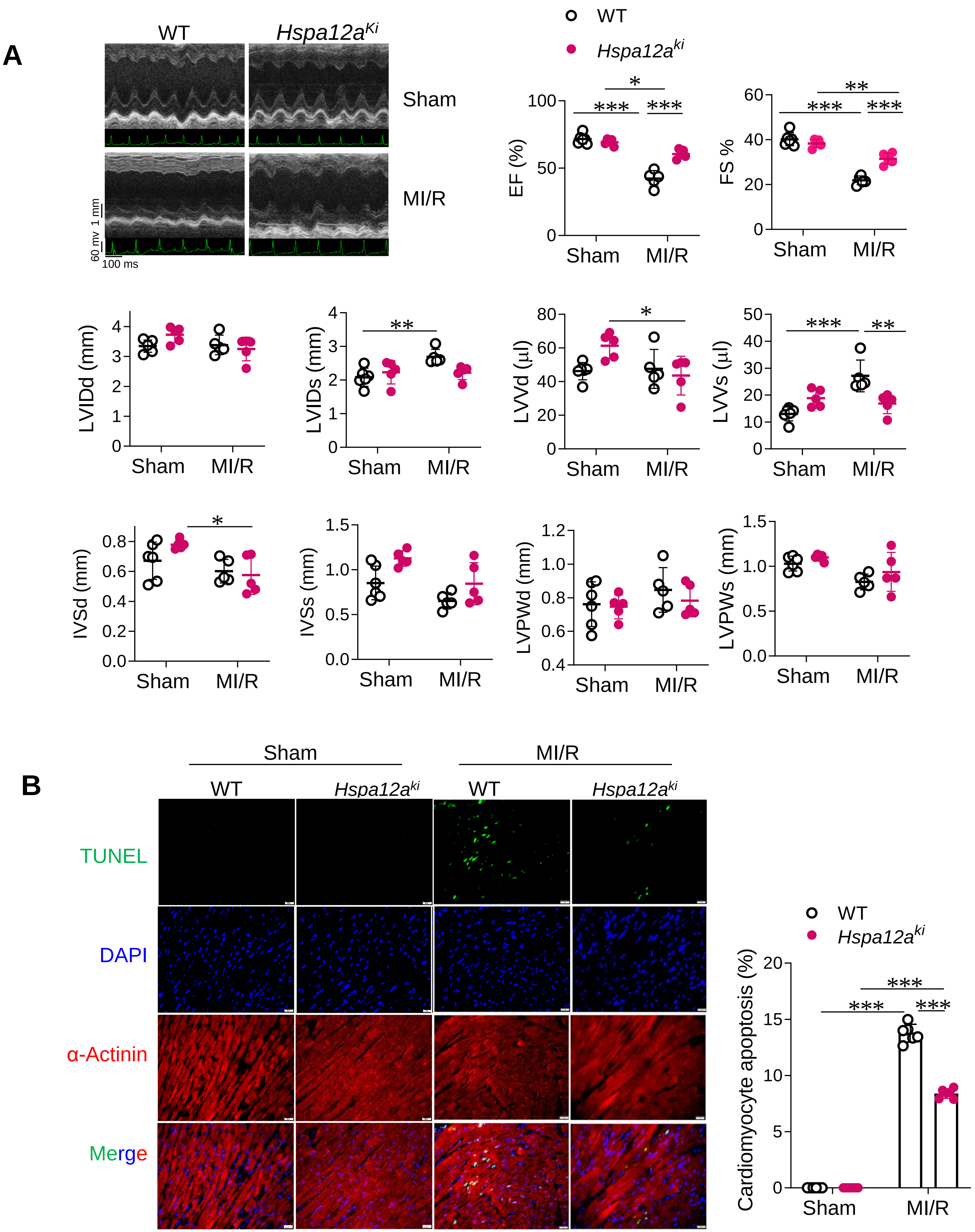
A
WT
Hspa12aKi
Sham
MI/R
60 mv
1 mm
100 ms
WT
Hspa12aki
EF (%)
FS %
LVIDd (mm)
LVIDs (mm)
LVVd (µl)
LVVs (µl)
IVSd (mm)
IVSs (mm)
LVPWd (mm)
LVPWs (mm)
B
TUNEL
DAPI
α-Actinin
Merge
Cardiomyocyte apoptosis (%)

Figure S5

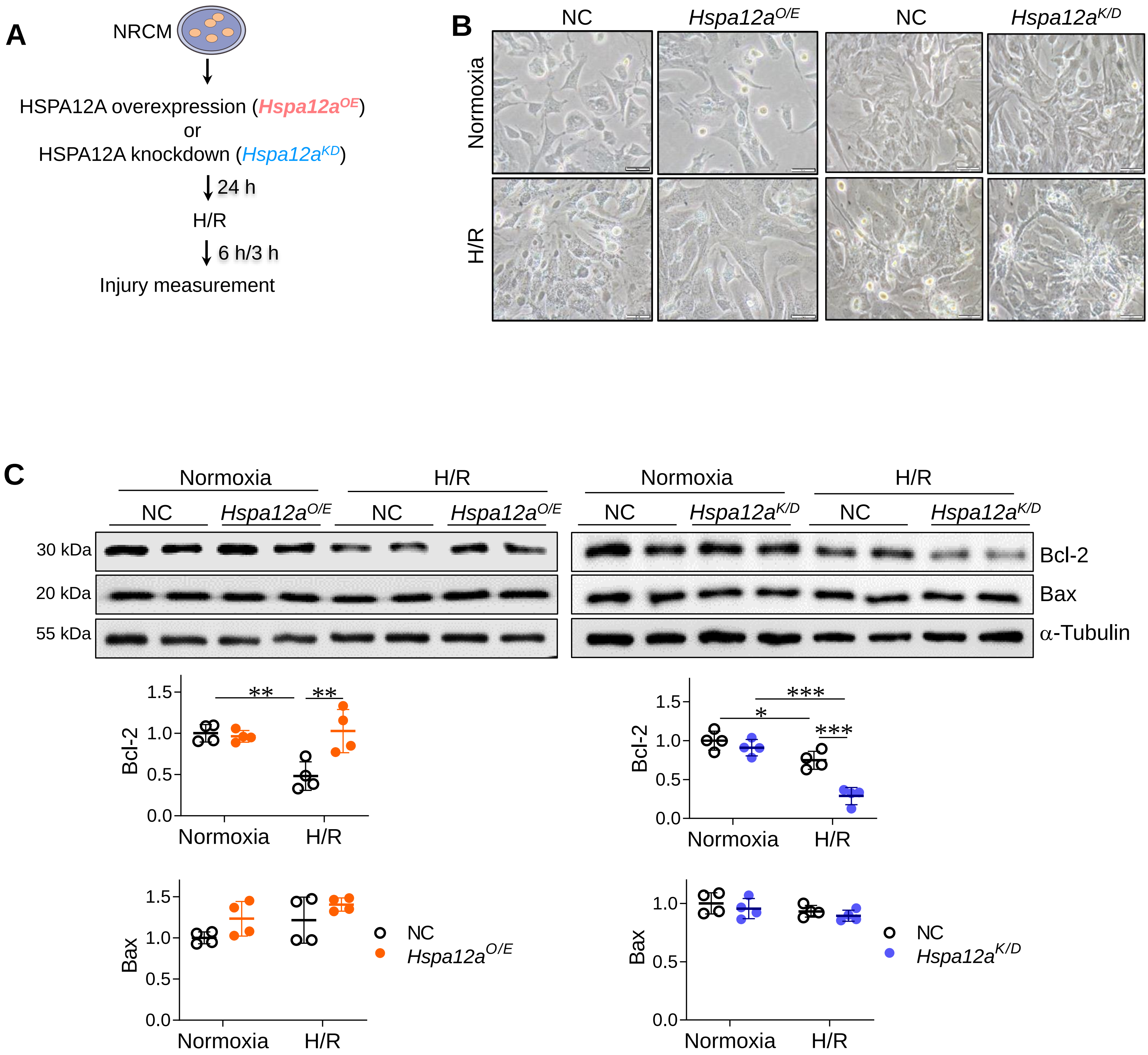
A
NRCM
HSPA12A overexpression (Hspa12a^OE)
or
HSPA12A knockdown (Hspa12a^KD)
24 h
H/R
6 h/3 h
Injury measurement
B
NC
Hspa12a^O/E
NC
Hspa12a^K/D
Normoxia
H/R
C
Normoxia
H/R
NC
Hspa12a^O/E
Hspa12a^K/D
30 kDa
20 kDa
55 kDa
Bcl-2
Bax
α-Tubulin
1.5
1.0
0.5
0.0
**
***
*
Normoxia
H/R
NC
Hspa12a^O/E
Hspa12a^K/D

Figure S6

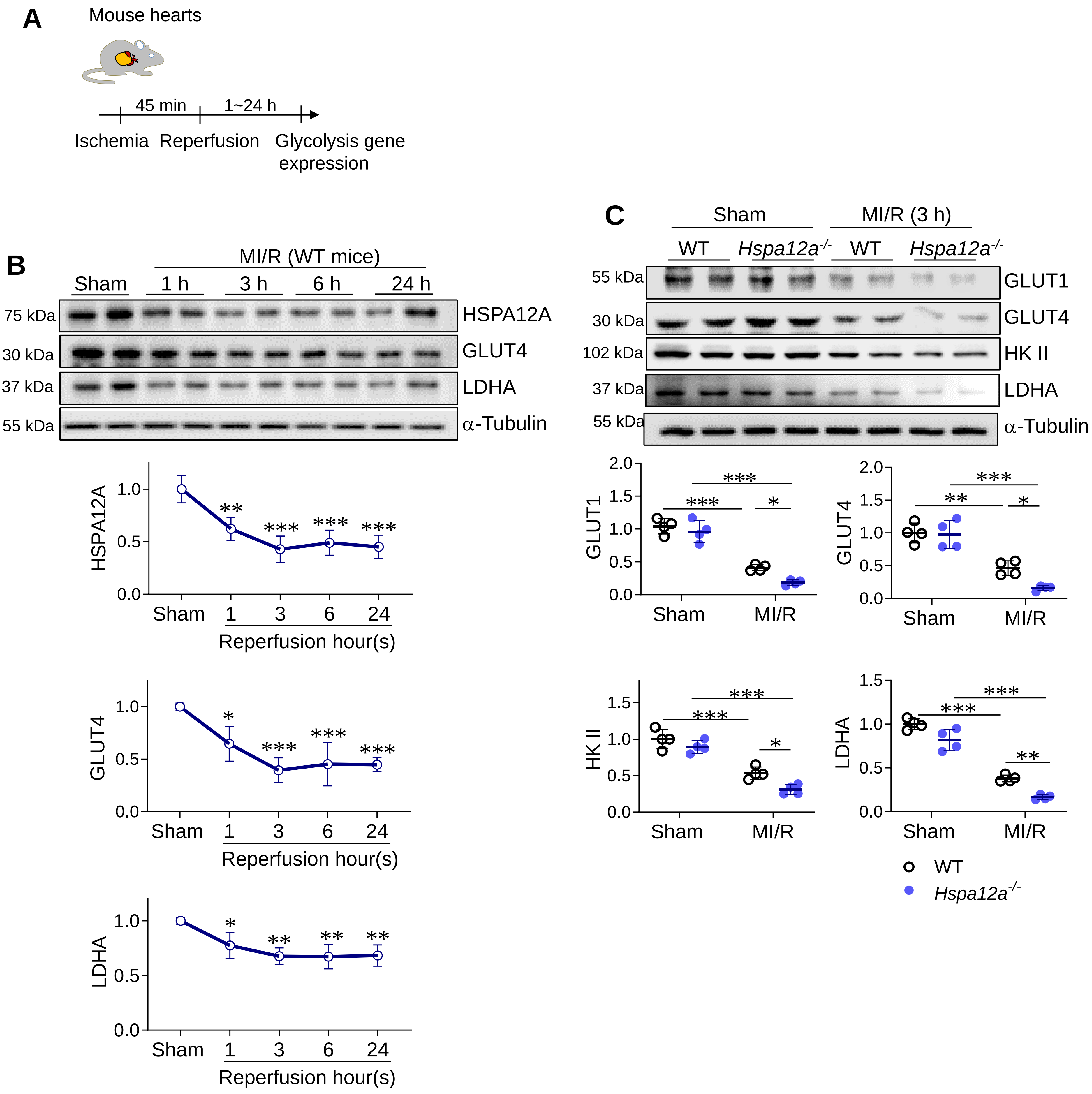
A
Mouse hearts
45 min
1~24 h
Ischemia
Reperfusion
Glycolysis gene expression
B
MI/R (WT mice)
Sham
1 h
3 h
6 h
24 h
75 kDa
30 kDa
37 kDa
55 kDa
HSPA12A
GLUT4
LDHA
α-Tubulin
Reperfusion hour(s)
C
Sham
MI/R (3 h)
WT
Hspa12a-/-
102 kDa
GLUT1
HK II
MI/R
WT
Hspa12a-/-

Figure S7

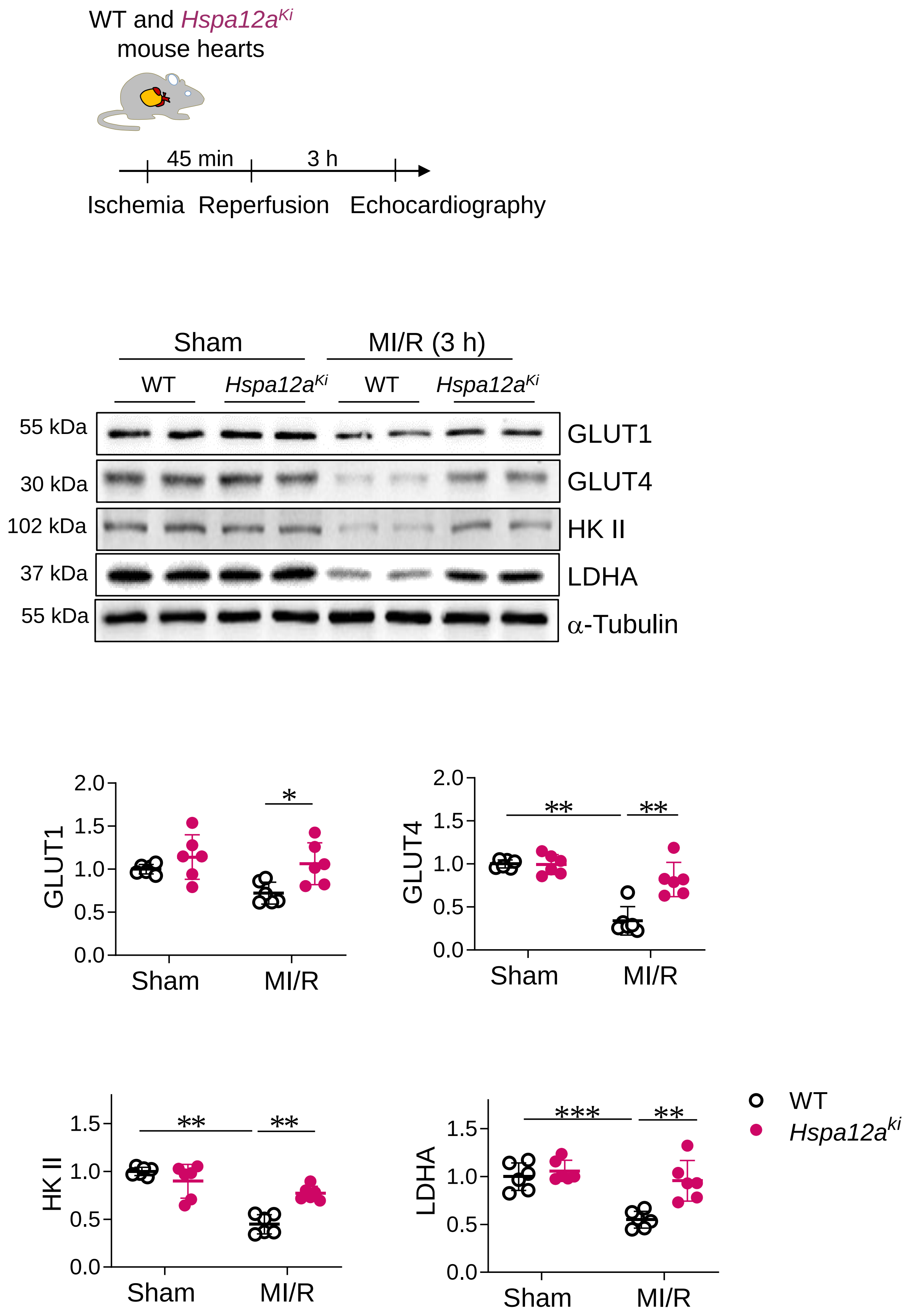

WT and Hspa12aKi
mouse hearts
45 min
3 h
Ischemia
Reperfusion
Echocardiography
Sham
MI/R (3 h)
WT
Hspa12aKi
WT
Hspa12aKi
55 kDa
30 kDa
102 kDa
37 kDa
55 kDa
GLUT1
GLUT4
HK II
LDHA
α-Tubulin
GLUT1
GLUT4
HK II
LDHA
Sham
MI/R
WT
Hspa12aki

Figure S8

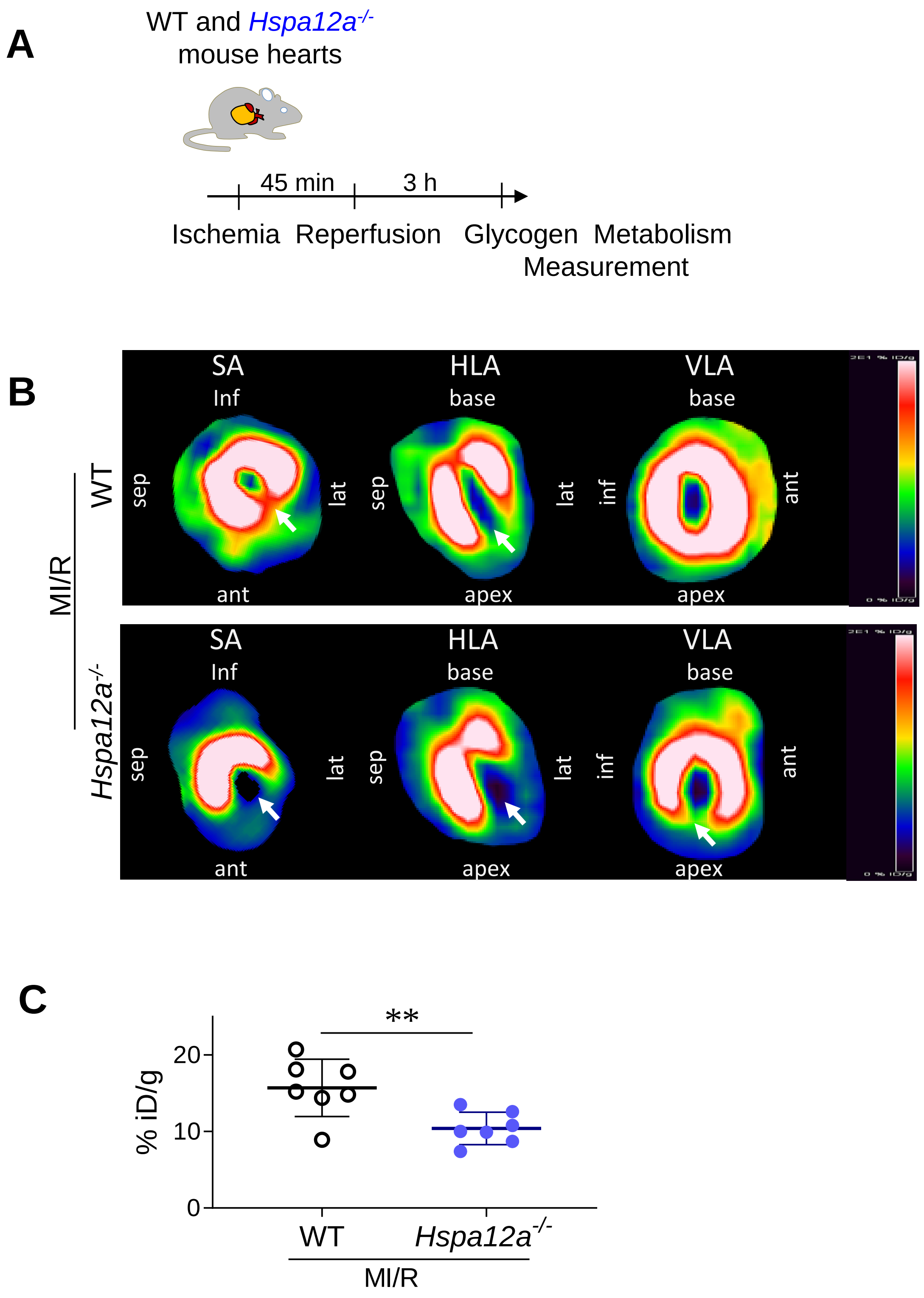
A
WT and Hspa12a-/-
mouse hearts
45 min
3 h
Ischemia
Reperfusion
Glycogen Metabolism
Measurement
B
MI/R
WT
Hspa12a-/-
SA
Inf
sep
lat
ant
HLA
base
sep
lat
apex
VLA
base
inf
ant
apex
C
% iD/g
20
10
0
**
WT
Hspa12a-/-
MI/R

Figure S9

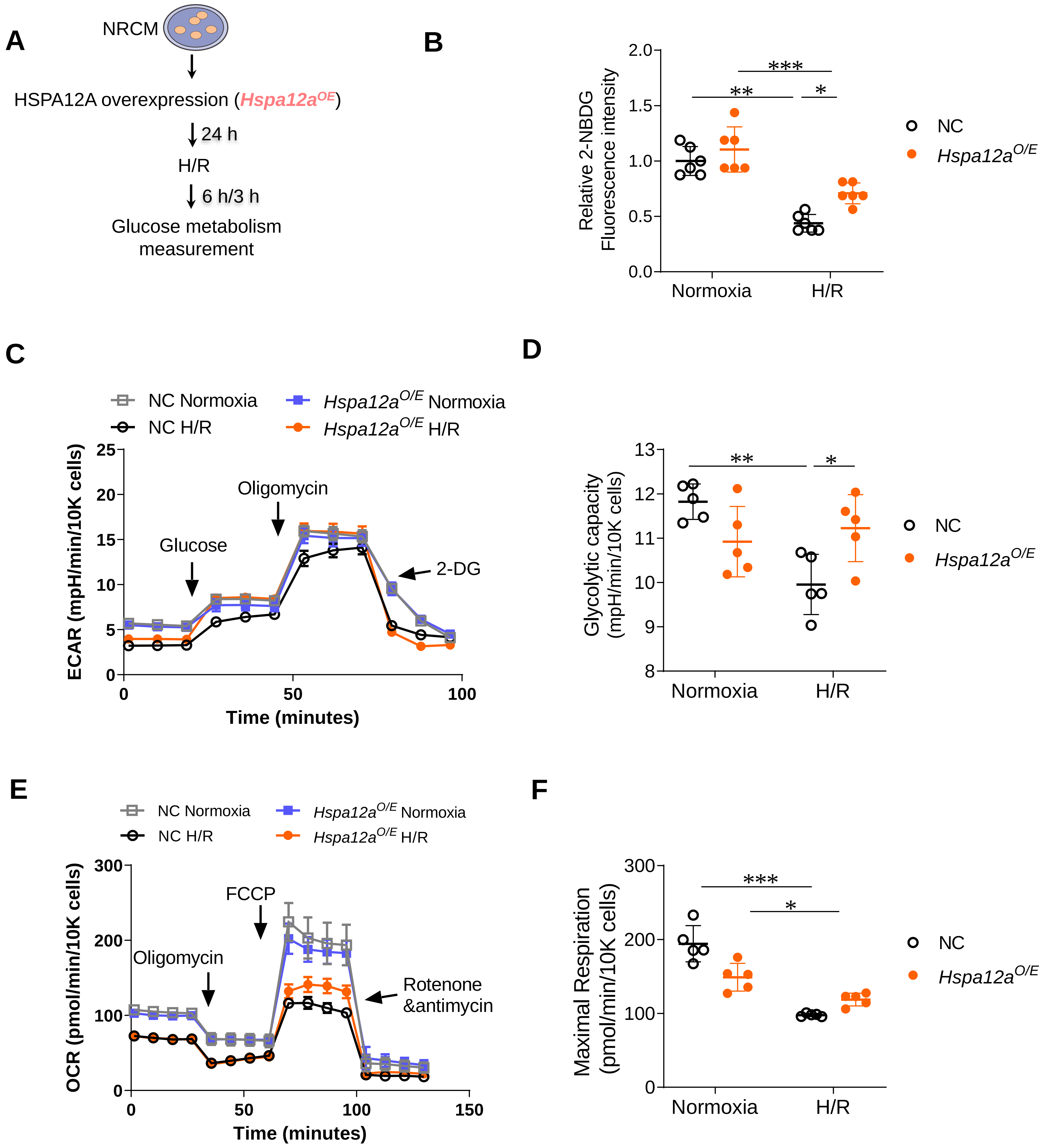
A
NRCM
HSPA12A overexpression (Hspa12a^OE)
24 h
H/R
6 h/3 h
Glucose metabolism measurement
B
Relative 2-NBDG Fluorescence intensity
2.0
1.5
1.0
0.5
0.0
Normoxia
H/R
***
**
*
NC
Hspa12a^O/E
C
NC Normoxia
NC H/R
Hspa12a^O/E Normoxia
Hspa12a^O/E H/R
ECAR (mpH/min/10K cells)
25
20
15
10
5
0
Glucose
Oligomycin
2-DG
50
100
Time (minutes)
D
Glycolytic capacity (mpH/min/10K cells)
13
12
11
10
9
8
Normoxia
H/R
**
*
NC
Hspa12a^O/E
E
NC Normoxia
NC H/R
Hspa12a^O/E Normoxia
Hspa12a^O/E H/R
OCR (pmol/min/10K cells)
300
200
100
0
Oligomycin
FCCP
Rotenone &antimycin
50
100
150
Time (minutes)
F
Maximal Respiration (pmol/min/10K cells)
300
200
100
0
Normoxia
H/R
***
*
NC
Hspa12a^O/E

Figure S10

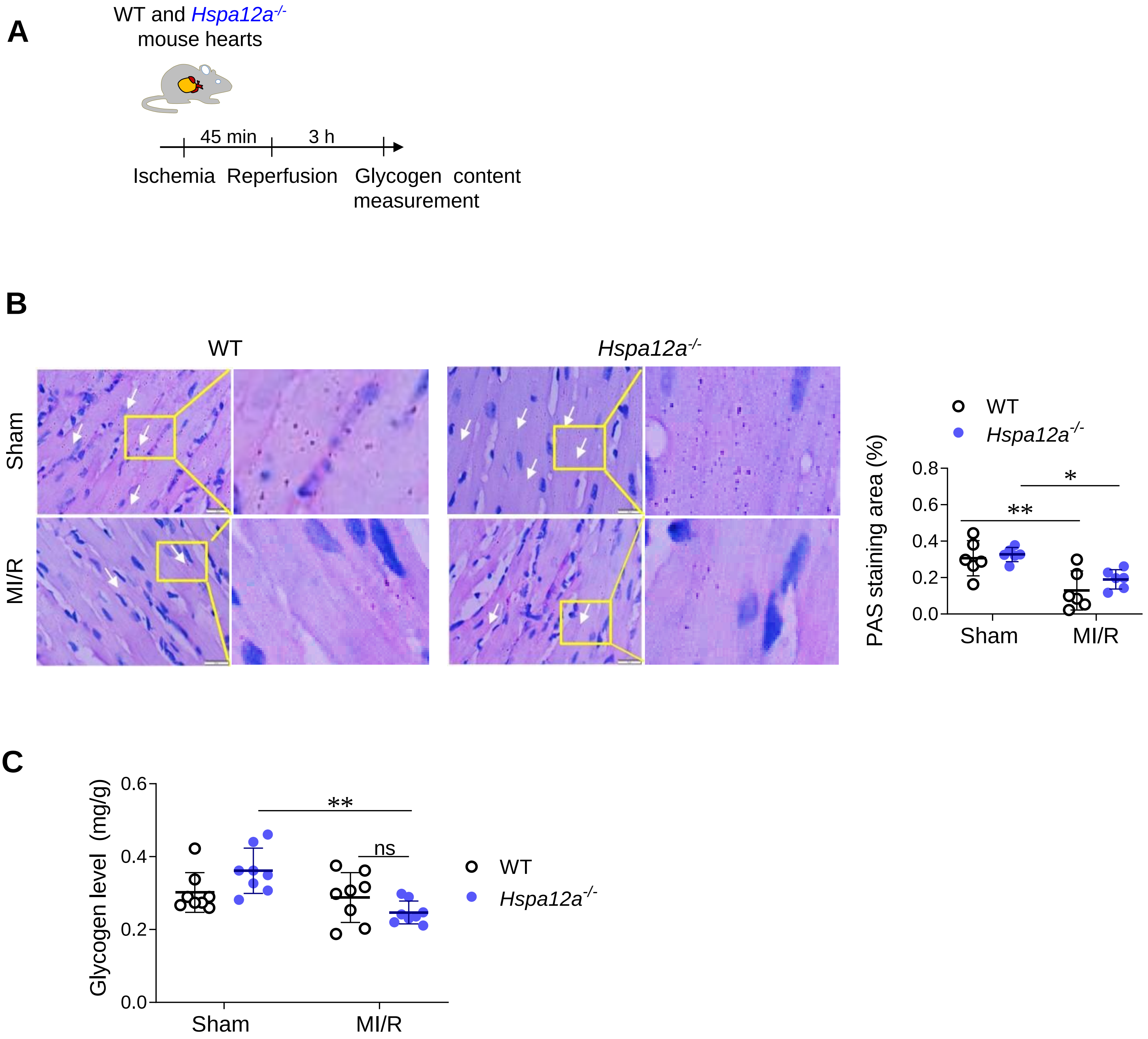
A
WT and Hspa12a-/-
mouse hearts
45 min
3 h
Ischemia
Reperfusion
Glycogen content
measurement
B
WT
Hspa12a-/-
Sham
MI/R
WT
Hspa12a-/-
PAS staining area (%)
0.8
0.6
0.4
0.2
0.0
**
*
Sham
MI/R
C
Glycogen level (mg/g)
0.6
0.4
0.2
0.0
**
ns
WT
Hspa12a-/-
Sham
MI/R

Figure S11

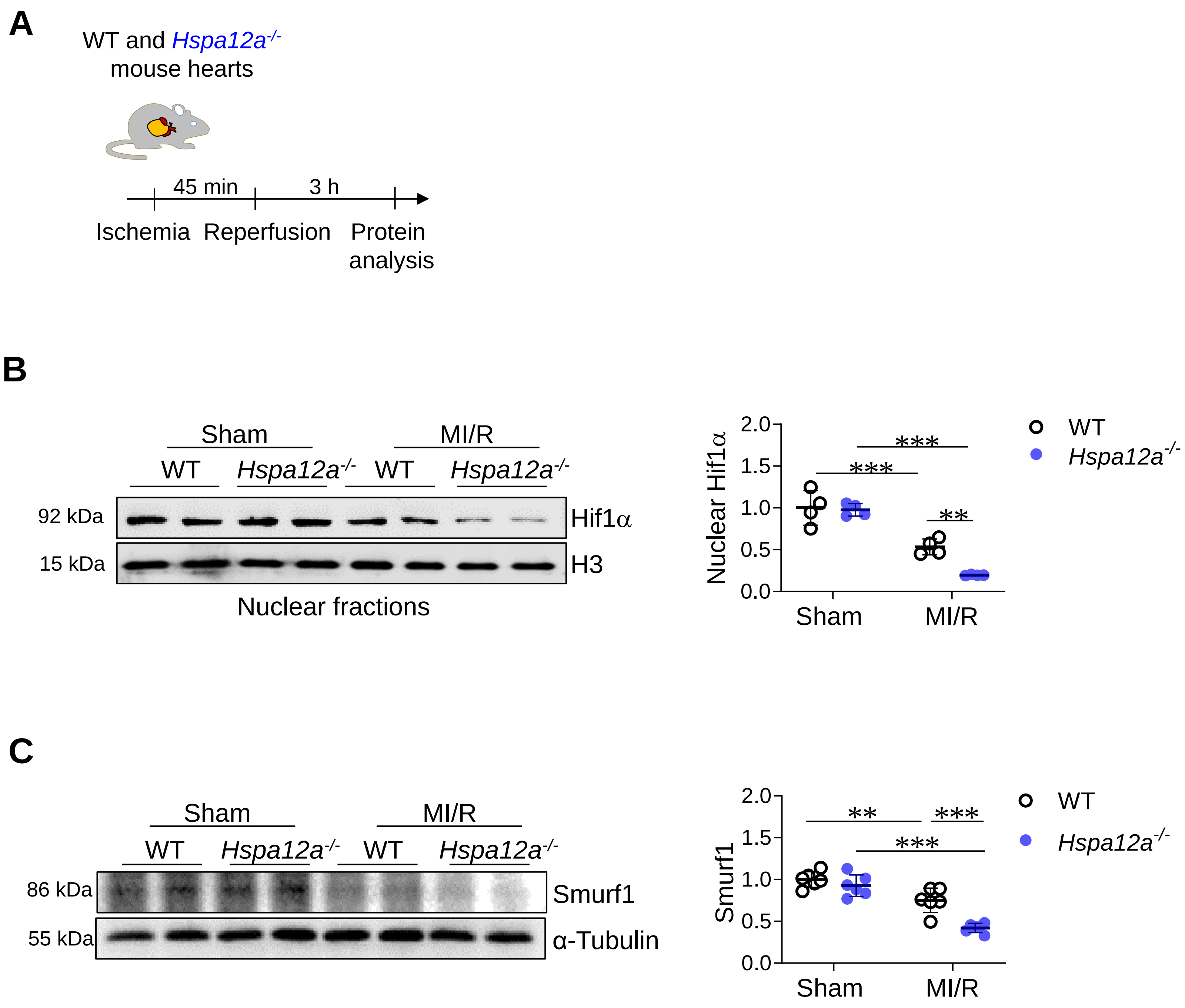
A
WT and Hspa12a-/-
mouse hearts
45 min
3 h
Ischemia
Reperfusion
Protein
analysis
B
Sham
MI/R
WT
Hspa12a-/-
WT
Hspa12a-/-
92 kDa
Hif1α
15 kDa
H3
Nuclear fractions
Nuclear Hif1α
2.0
1.5
1.0
0.5
0.0
***
***
**
Sham
MI/R
WT
Hspa12a-/-
C
Sham
MI/R
WT
Hspa12a-/-
WT
Hspa12a-/-
86 kDa
Smurf1
55 kDa
α-Tubulin
Smurf1
2.0
1.5
1.0
0.5
0.0
**
***
***
Sham
MI/R
WT
Hspa12a-/-

Figure S12

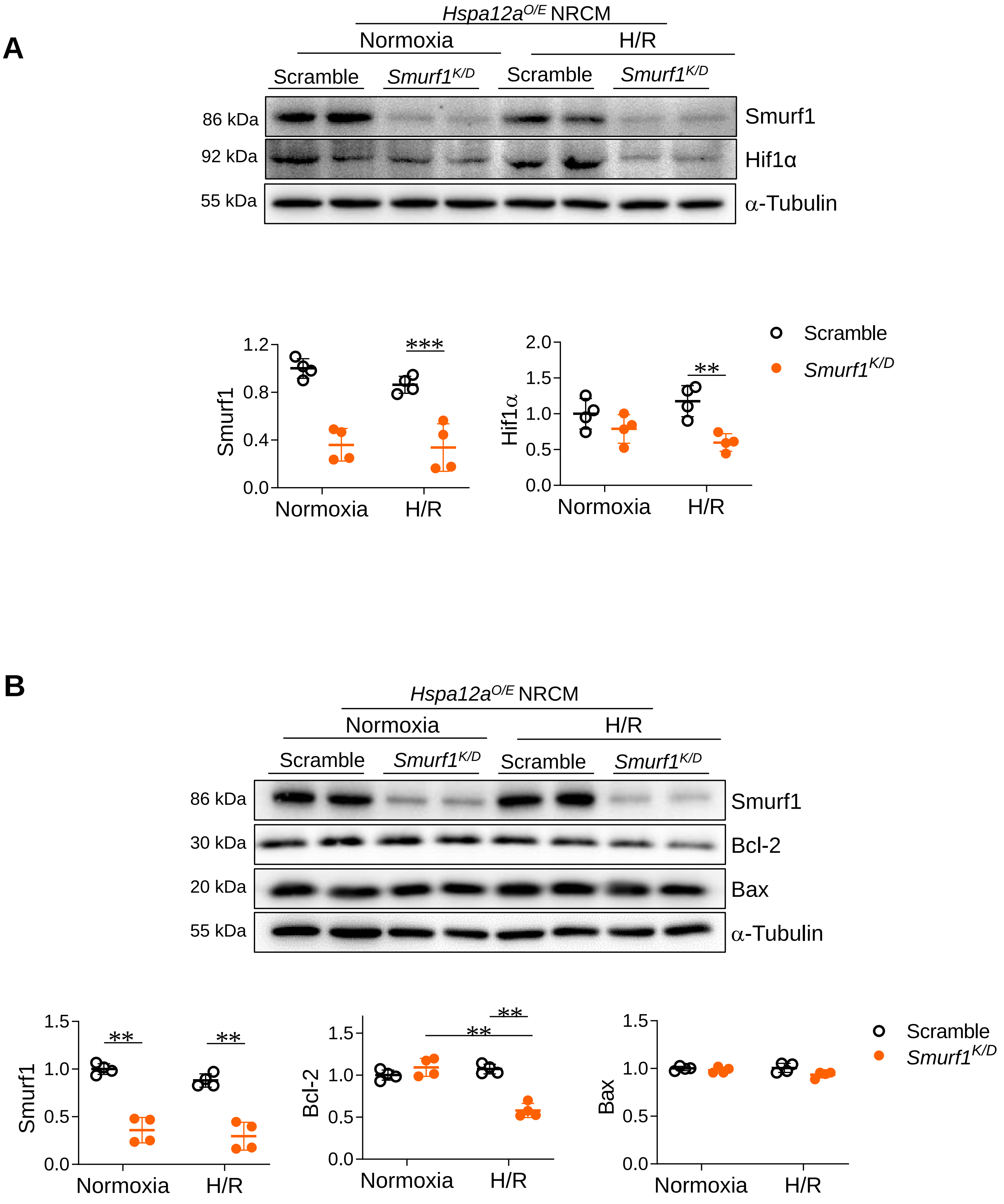
A
Hspa12aO/E NRCM
Normoxia
H/R
Scramble
Smurf1K/D
86 kDa
92 kDa
55 kDa
Smurf1
Hif1α
α-Tubulin
Smurf1
Hif1α
Normoxia
H/R
***
**
Scramble
Smurf1K/D
B
Hspa12aO/E NRCM
86 kDa
30 kDa
20 kDa
55 kDa
Smurf1
Bcl-2
Bax
α-Tubulin
Smurf1
Bcl-2
Bax
**

Figure S13

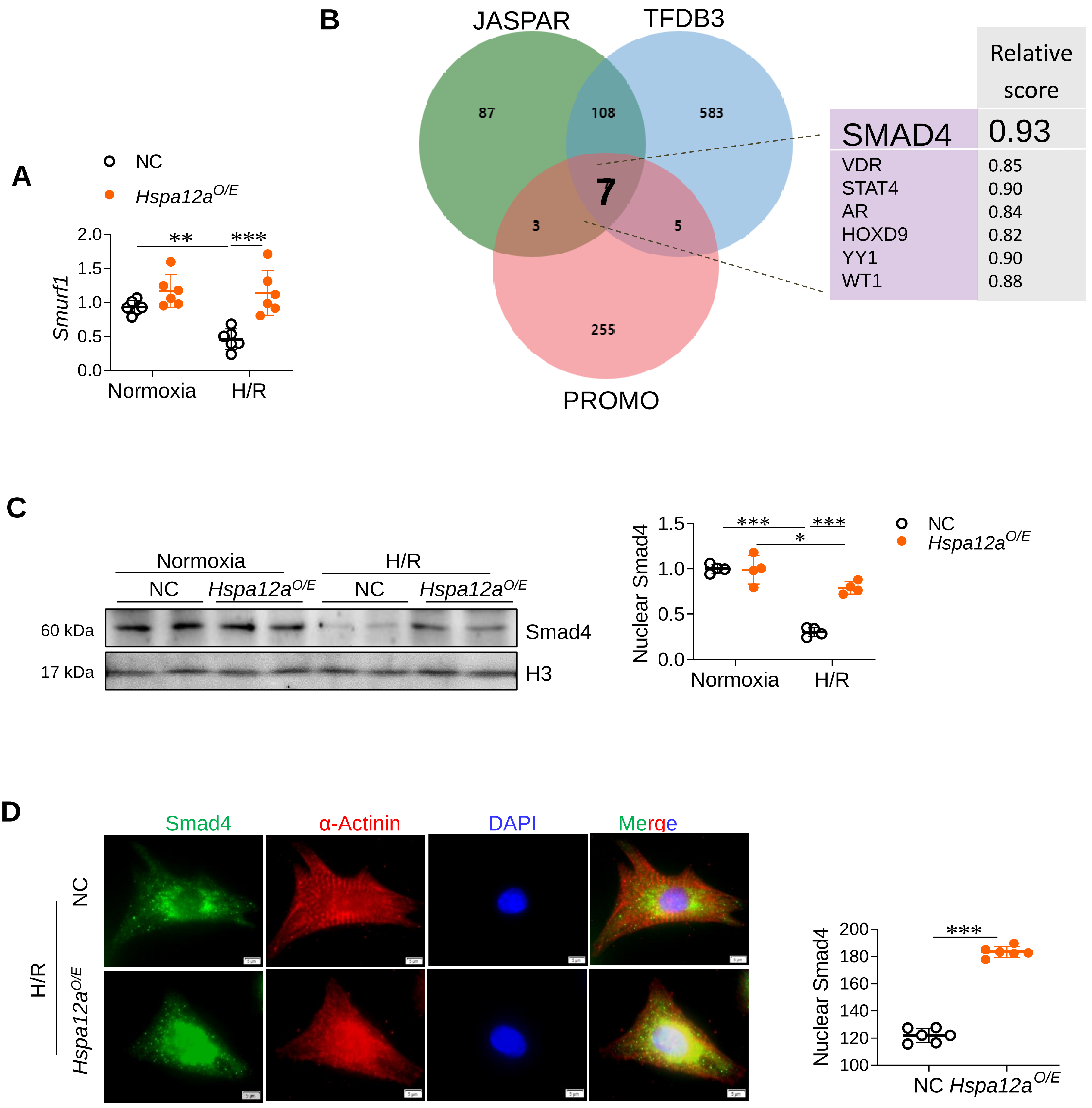
A
NC
Hspa12aO/E
Smurf1
2.0
1.5
1.0
0.5
0.0
**
***
Normoxia
H/R
B
JASPAR
TFDB3
PROMO
87
108
583
7
3
5
255
Relative score
SMAD4 0.93
VDR 0.85
STAT4 0.90
AR 0.84
HOXD9 0.82
YY1 0.90
WT1 0.88
C
Normoxia
H/R
NC
Hspa12aO/E
60 kDa
17 kDa
Smad4
H3
Nuclear Smad4
*
D
Smad4
α-Actinin
DAPI
Merge
200
180
160
140
120
100

Figure S14

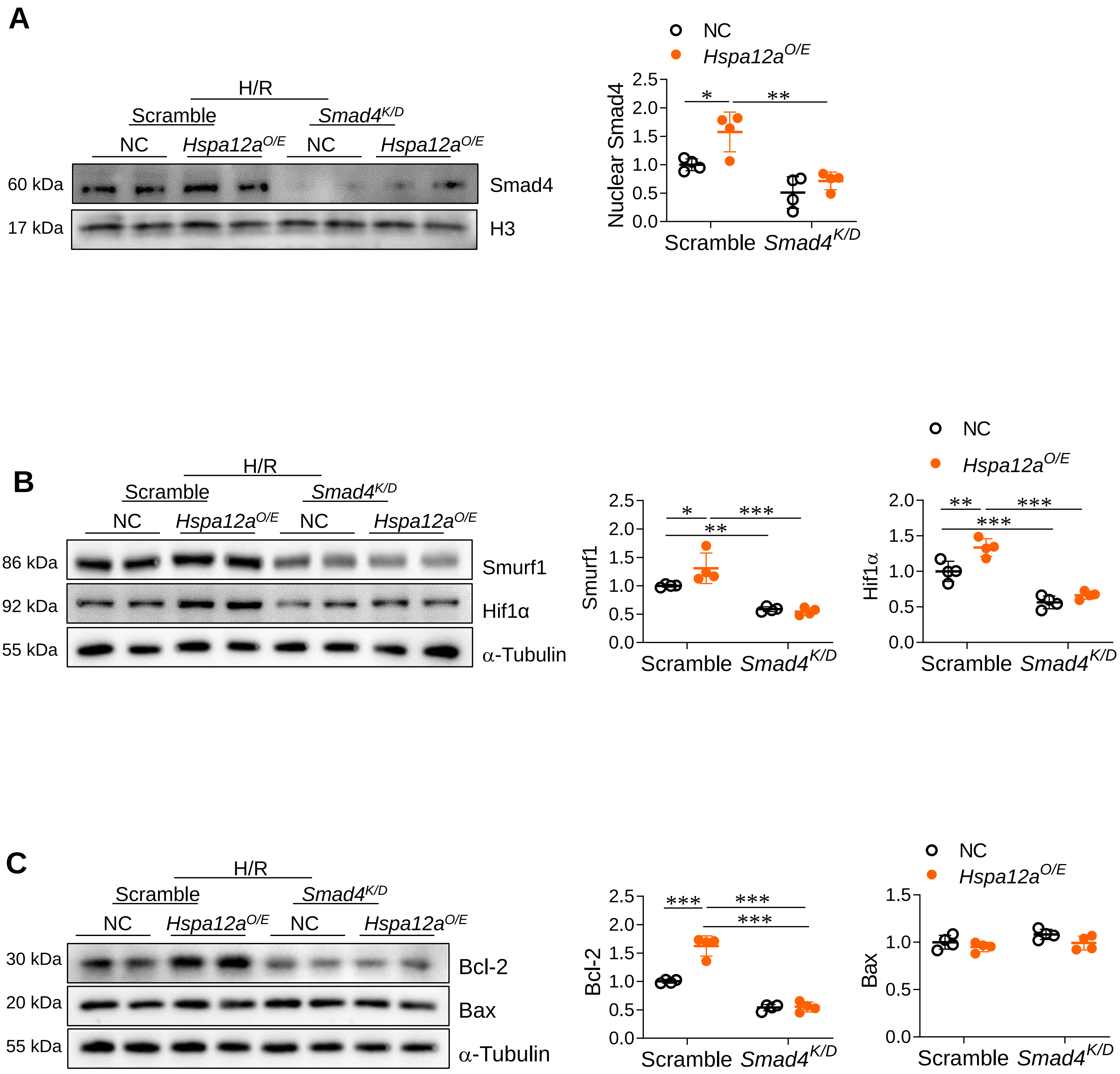
A
NC
Hspa12a O/E
H/R
Scramble
Smad4 K/D
NC
Hspa12a O/E
NC
Hspa12a O/E
60 kDa
Smad4
17 kDa
H3
Nuclear Smad4
2.5
2.0
1.5
1.0
0.5
0.0
*
**
Scramble
Smad4 K/D
B
H/R
Scramble
Smad4 K/D
NC
Hspa12a O/E
NC
Hspa12a O/E
86 kDa
Smurf1
92 kDa
Hif1α
55 kDa
α-Tubulin
Smurf1
*
***
**
Hif1α
**
***
***
NC
Hspa12a O/E
C
H/R
Scramble
Smad4 K/D
NC
Hspa12a O/E
NC
Hspa12a O/E
30 kDa
Bcl-2
20 kDa
Bax
55 kDa
α-Tubulin
Bcl-2
***
***
***
Bax
NC
Hspa12a O/E

Figure S15

Supplemental materials

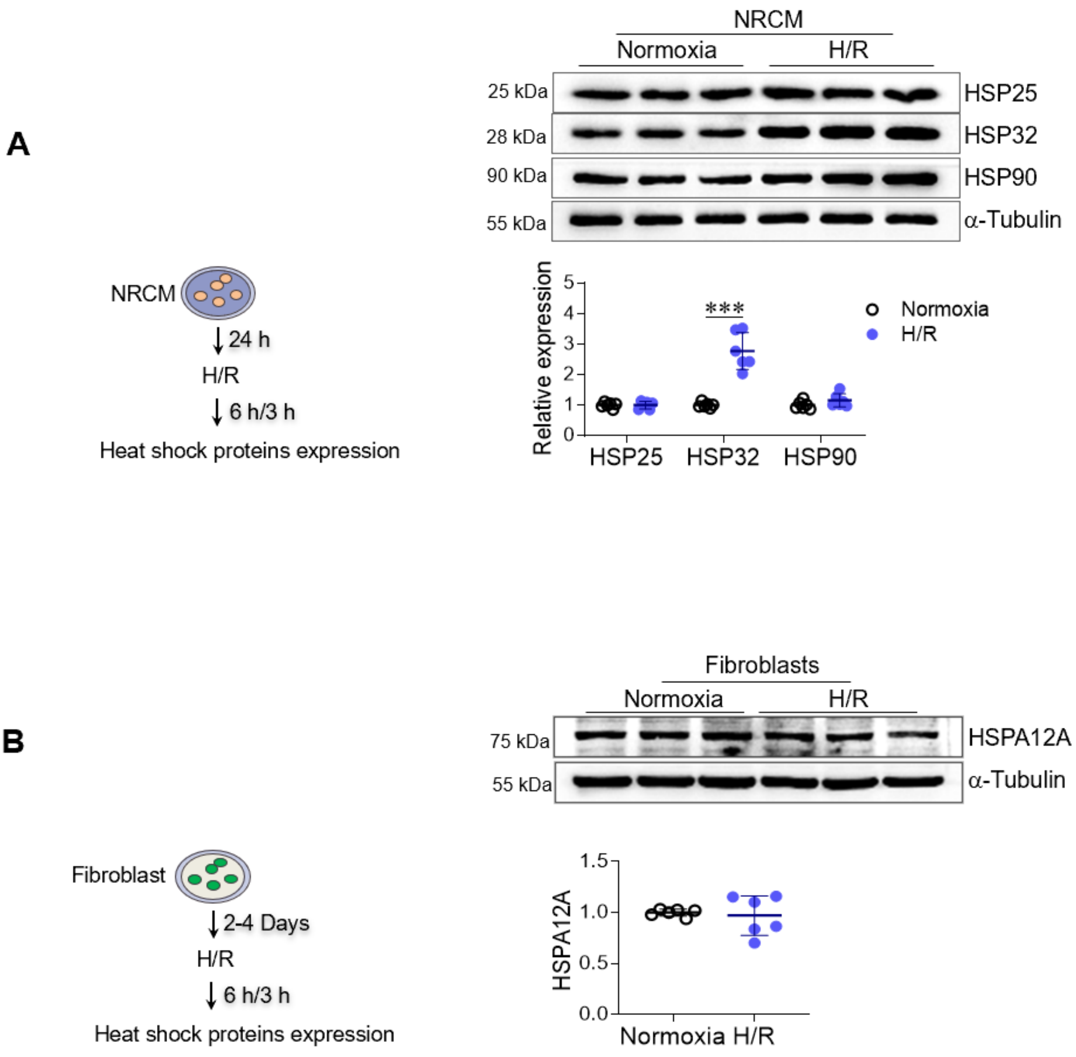

Figure 1. Heat shock protein expression in cardiomyocytes and fibroblasts following H/R.

A. Primary cardiomyocytes (NRCM) were subjected to H/R (6 h/3 h). After H/R, the indicated gene expression was examined using immunoblotting analysis. The blots against α-Tubulin served as loading controls.

B. Primary cardiac fibroblasts were isolated from hearts of neonatal rats (1–3 day-old). After H/R (6 h/3 h), HSPA12A expression was examined using immunoblotting. The blots against α-Tubulin served as loading controls.

Data are means ± SD, ****P* < 0.001 by Student's two-tailed unpaired *t* test. n = 6/group.

Abbreviations: NRCM: primary cardiomyocytes; H/R: hypoxia/reoxygenation.

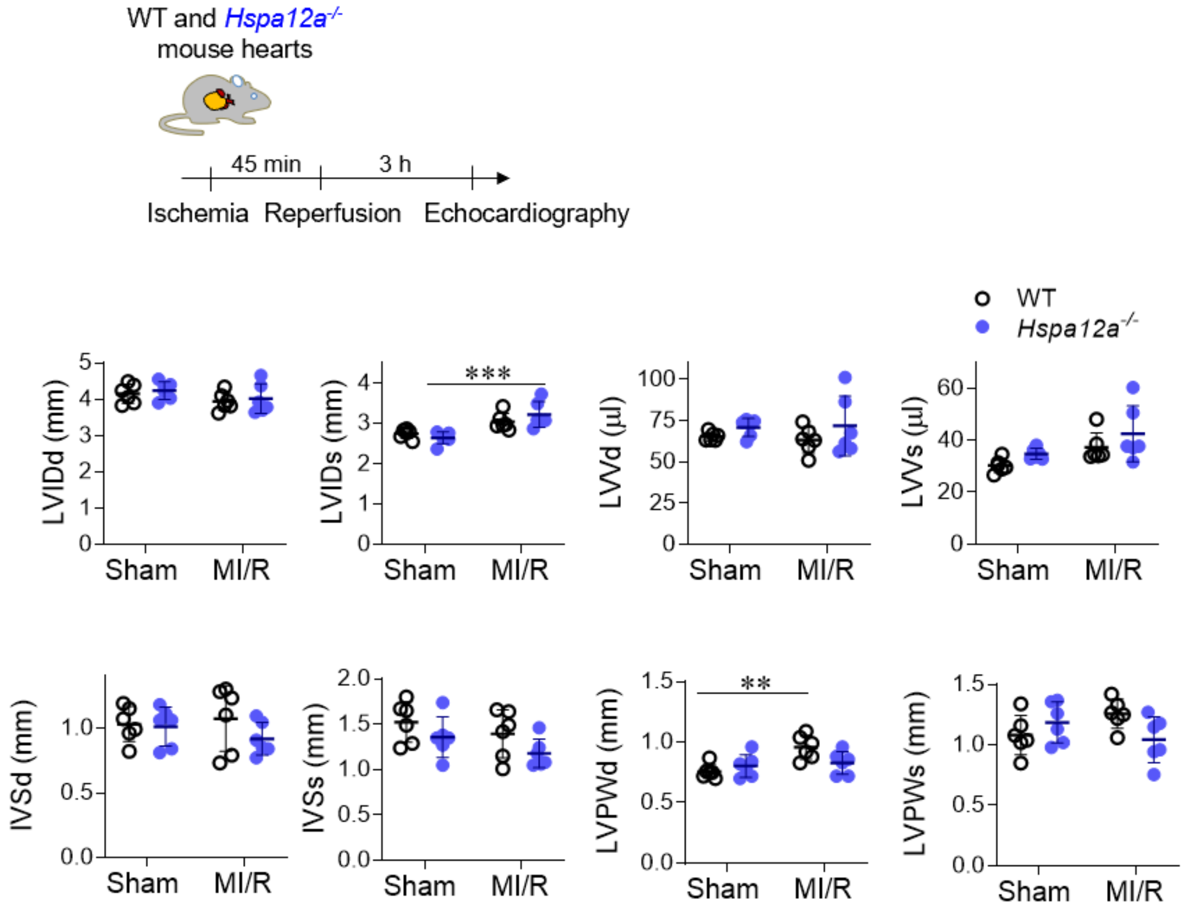

Figure 2. HSPA12A knockout exacerbated the cardiac dysfunction after MI/R for 3 h in mice.

Cardiac performance was examined using two-dimensional echocardiography after MI/R. Data are means ± SD, ***$P < 0.001$ and **$P < 0.01$ by two-way ANOVA followed by post hoc test (LVIDd, LVIDs, LVSs, LVPWd and LVPWs) or Krystal-Wallis test (LVVd, LVVs and LVSd). n = 6/group.

Abbreviations: LVIDd: left ventricular internal diameter at diastolic phase; LVIDs: left ventricular internal diameter at systolic phase; LVVd: left ventricular end-diastolic volume; LVVs: left ventricular end-systolic volume; IVSd: inter ventricular septum thickness at diastolic phase; IVSs: inter ventricular septum thickness at systolic phase; LVPWd: left ventricular posterior wall thickness at diastolic phase; LVPWs: left ventricular posterior wall thickness at systolic phase.

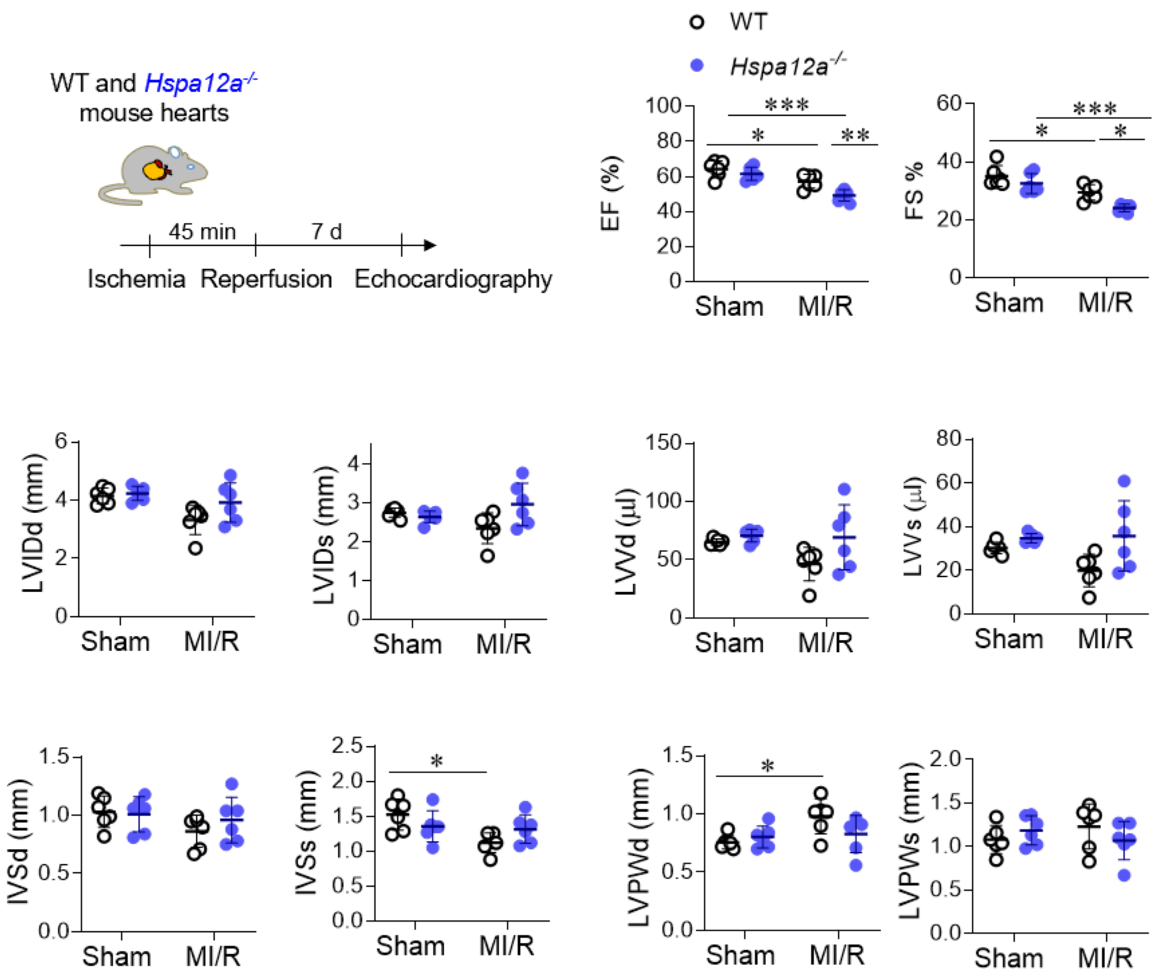

Figure 3. HSPA12A knockout exacerbated the cardiac dysfunction after MI/R for 7 days in mice.

Cardiac performance was examined using two-dimensional echocardiography after MI/R for 7 days. Data are means ± SD, ***$P < 0.001$, **$P < 0.01$ and *$P<0.05$ by two-way ANOVA followed by post hoc test (EF%, FS%, LVSd, LVSs, LVPWd and LVPWs) or Krystal-Wallis test (LVIDd, LVIDs, LVVd, and LVVs). n = 6/group.

Abbreviations: EF%, ejection fraction; FS%, fraction shortening; LVIDd: left ventricular internal diameter at diastolic phase; LVIDs: left ventricular internal diameter at systolic phase; LVVd: left ventricular end-diastolic volume; LVVs: left ventricular end-systolic volume; IVSd: inter ventricular septum thickness at diastolic phase; IVSs: inter ventricular septum thickness at systolic phase; LVPWd: left ventricular posterior wall thickness at diastolic phase; LVPWs: left ventricular posterior wall thickness at systolic phase.

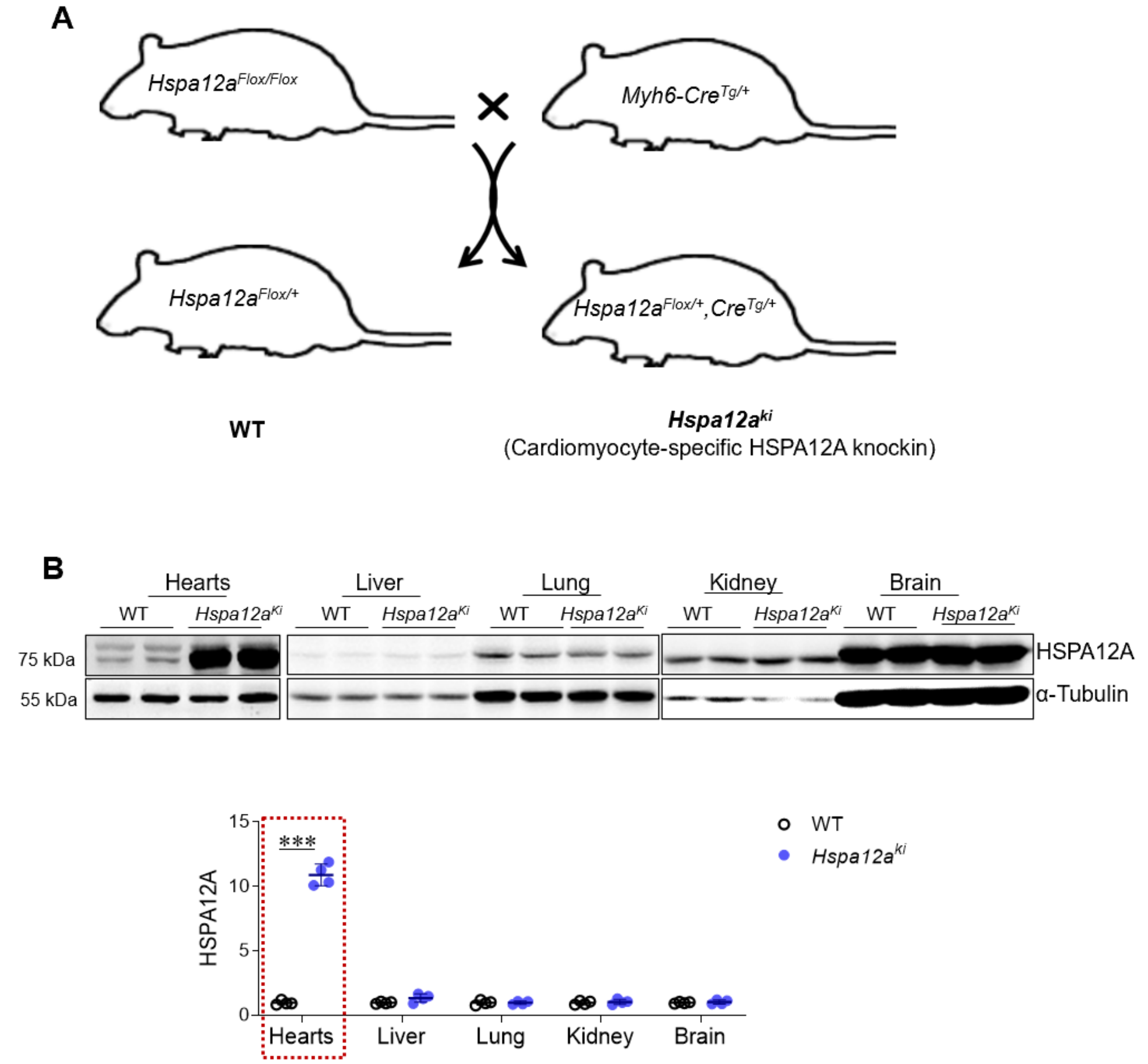

Figure 4. Creation of cardiomyocyte-specific HSPA12A overexpression mice (*Hspa12a^{ki}*).

A. Breading strategy for generating *Hspa12a^{ki}* mice by cross-breeding conditional *Hspa12a* knockin mice with *Myh6-Cre* transgenic mice.

B. Expression of HSPA12A was examined in heart, liver, lung, kidney and brain tissues. Data are means ± SD, ****P* < 0.001 by Student's two-tailed unpaired *t* test. n = 4/group.

Abbreviations: WT, wild type mice; *Hspa12a^{ki}*, cardiomyocyte-specific HSPA12A overexpression mice.

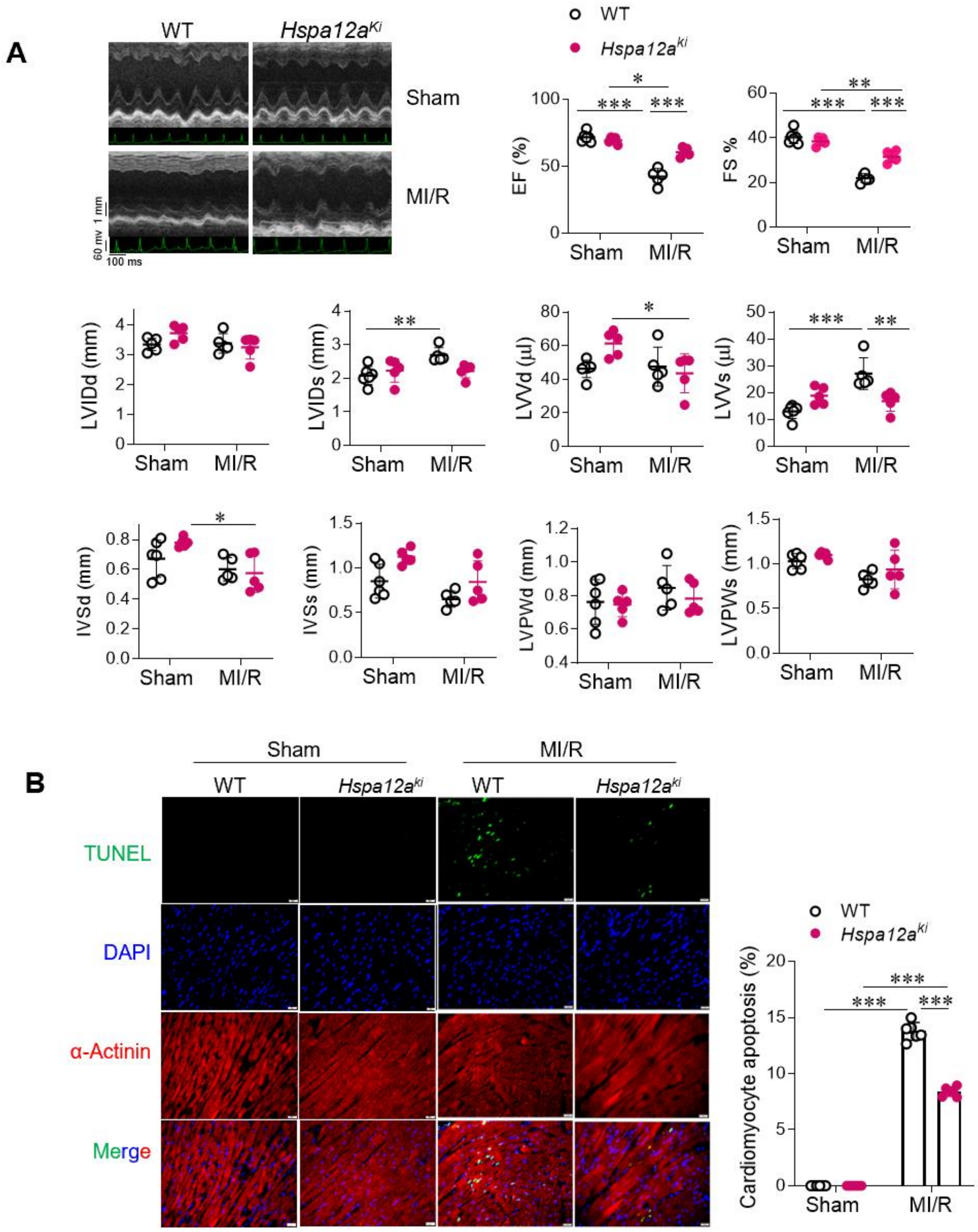

Figure 5. Cardiomyocyte-specific overexpression of HSPA12A alleviated MI/R injury.

A. Cardiac performance was examined using two-dimensional echocardiography after MI/R. Data are means ± SD, ****P* < 0.001, ***P* < 0.01 and **P*<0.05 by two-way ANOVA followed by post hoc test (EF%, FS%, LVIDd, LVIDs, LVVd, LVVs and LVPWd) or Krystal-Wallis test (LVSd, LVSs, LVPWs). n = 5-6/group.

B. Apoptosis in cardiomyocytes was examined after MI/R by TUNEL assay on the paraffin-embedded sections that prepared from cardiac tissues at papillary muscles. Alpha-Actinin was used to stain cardiomyocytes and DAPI was used to counterstain the nuclei. Scale bar = 50 µm. Data are means ± SD, ****P* < 0.001 by two-way ANOVA followed by post hoc test. n = 6/group.

Abbreviations: WT, wild type mice; *Hspa12a^{ki}*, cardiomyocyte-specific HSPA12A overexpression mice ; EF%, ejection fraction; FS%, fraction shortening; LVIDd: left ventricular internal diameter at diastolic phase; LVIDs: left ventricular internal diameter at systolic phase; LVVd: left ventricular end-diastolic volume; LVVs: left ventricular end-systolic volume; IVSd: inter ventricular septum thickness at diastolic phase; IVSs: inter ventricular septum thickness at systolic phase; LVPWd: left ventricular posterior wall thickness at diastolic phase; LVPWs: left ventricular posterior wall thickness at systolic phase.

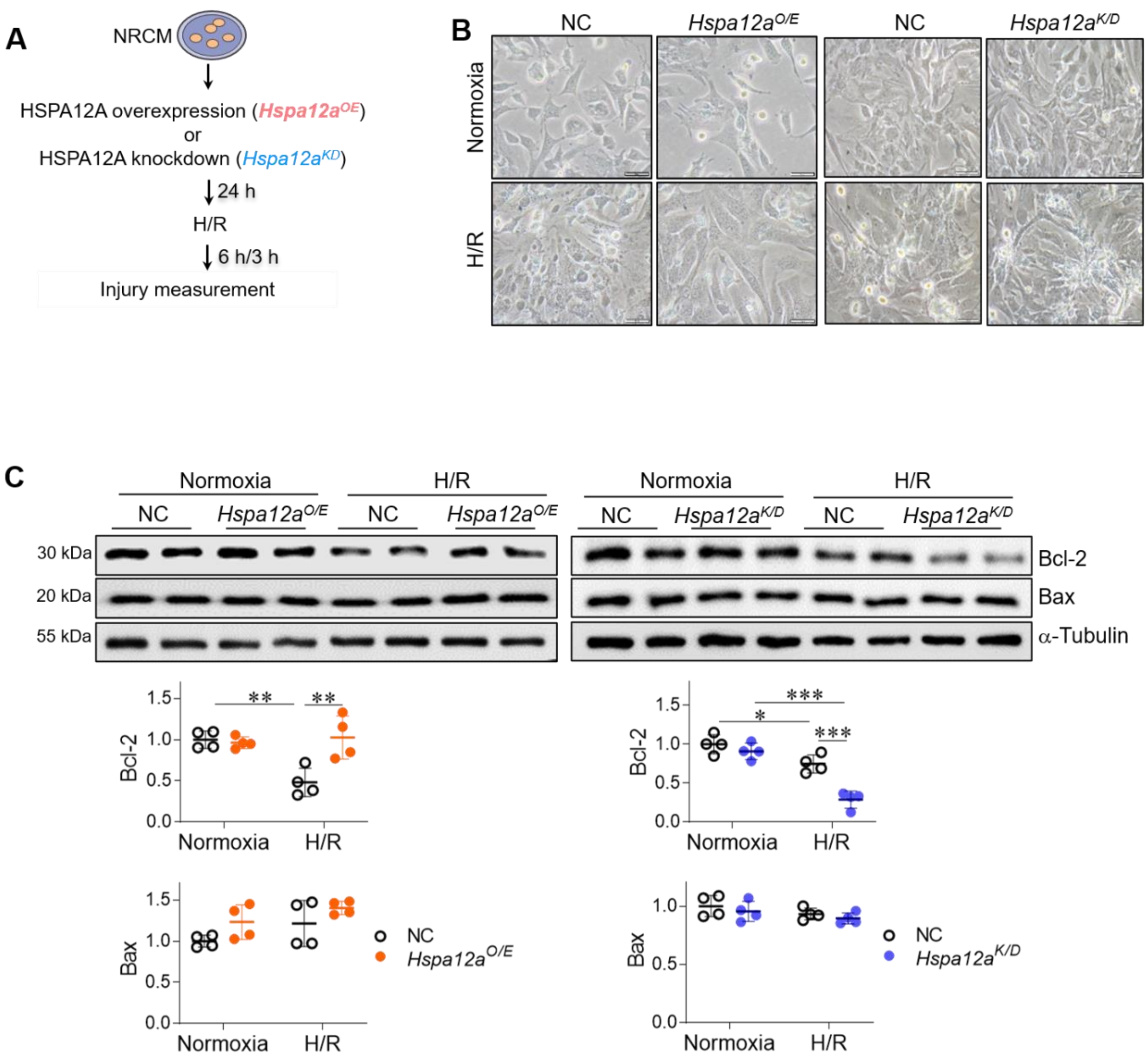

Figure 6. HSPA12A protected cardiomyocytes against H/R.

A. Experimental protocol of primary cardiomyocytes (NRCM).

B. Effects of HSPA12A on H/R-induced morphological abnormalities were observed using microscope at a magnification of 200 ×. Scale bar = 100 μm. n = 5/group.

C. Bcl-2 and Bax expression levels were examined using immunoblotting analysis. The blots against α-Tubulin served as loading controls. Data are means ± SD, ****P* < 0.001, ***P* < 0.01 and **P*<0.05 by two-way ANOVA followed by post hoc test. n = 4/group.

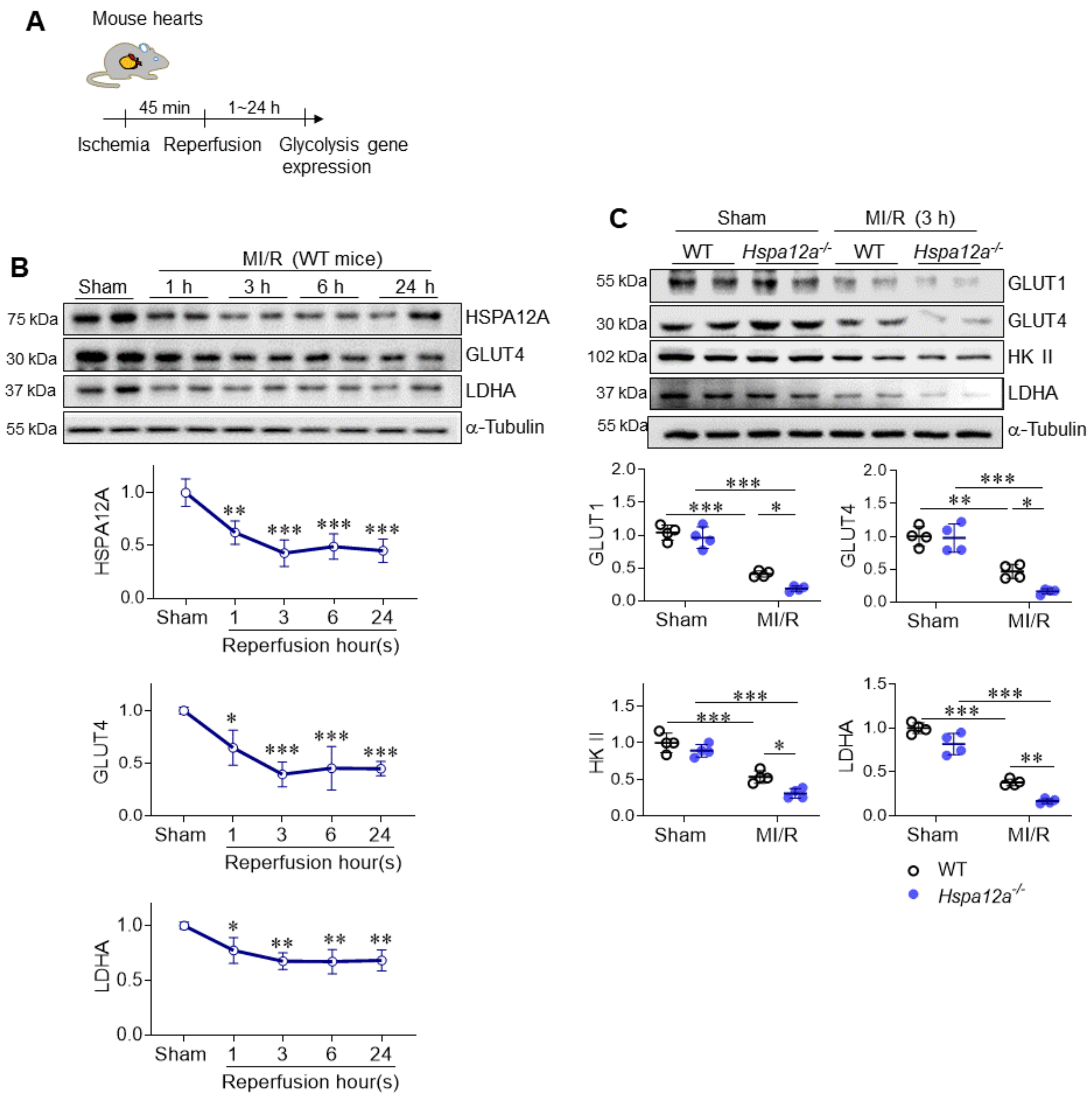

Figure 7. Knockout of HSPA12A in mice enhanced the MI/R-induced downregulation of glycolysis-related gene expression in hearts.

A. Experimental protocol.

B. Hearts of WT mice were subjected to ischemia for 45 min followed by reperfusion for different durations (1, 3, 6, and 24 h). Protein expression was analyzed by immunoblotting analysis. Data are means ± SD and analyzed using ordinary one-way ANOVA followed by post hoc test. ***$P < 0.001$, **$P < 0.01$ and *$P < 0.05$ vs. sham controls, n = 4/group.

C. The indicated gene expression was examined by immunoblotting analysis after reperfusion for 3 h following ischemia for 45 min. Data are means ± SD, ***$P < 0.001$, **$P < 0.01$, and *$P < 0.05$ by two-way ANOVA followed by post hoc test. n = 4/group.

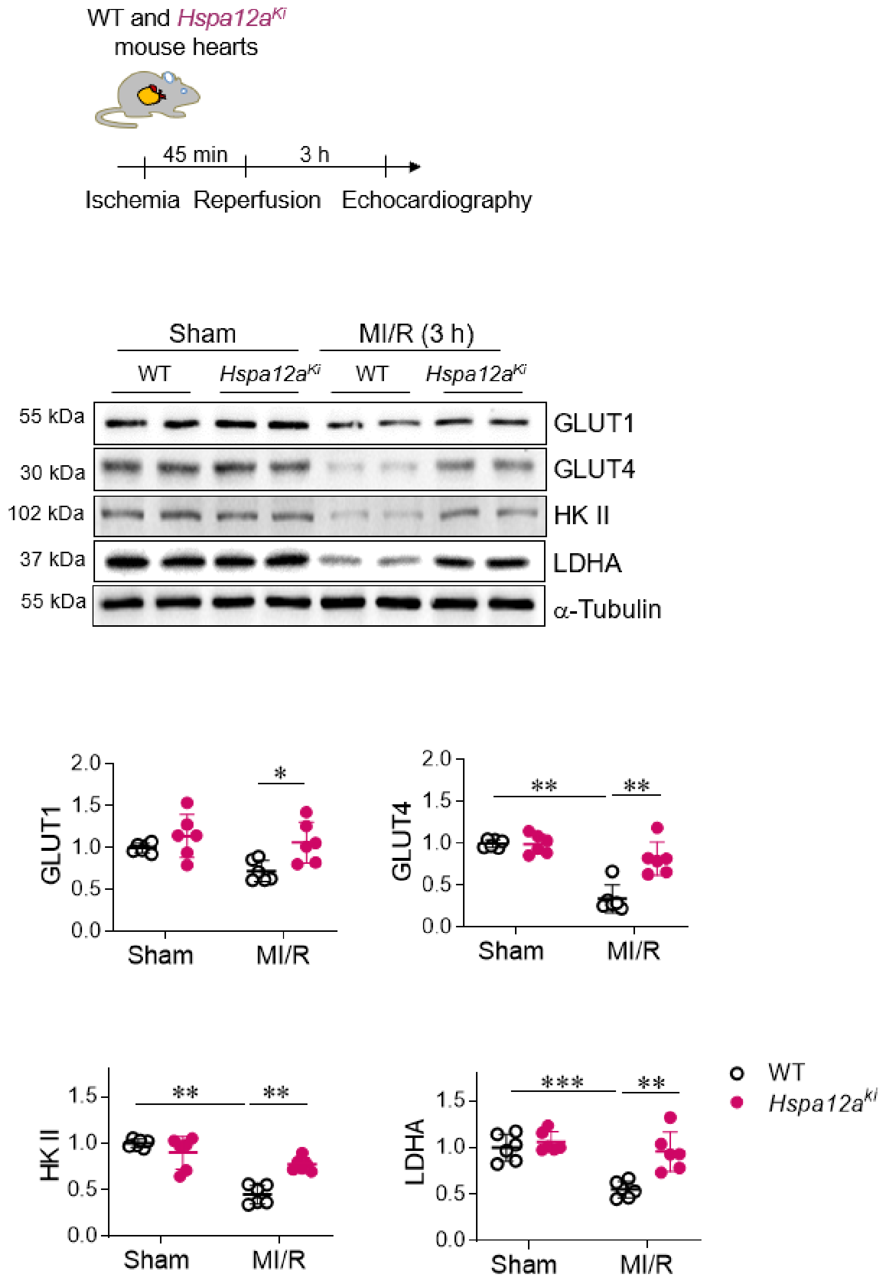

Figure 8. Cardiomyocyte-specific overexpression of HSPA12A increased glycolytic gene expression in mouse hearts after MI/R.

A. Experimental protocol.

B. The indicated gene expression was examined by immunoblotting analysis after MI/R (45 min/3 h). Data are means ± SD, ***$P < 0.001$, **$P < 0.01$, and *$P<0.05$ by two-way ANOVA followed by post hoc test. n = 6/group.

Abbreviations: WT, wild type mice; *Hspa12a^{ki}*, cardiomyocyte-specific HSPA12A overexpression mice; MI/R, myocardial ischemia/reperfusion.

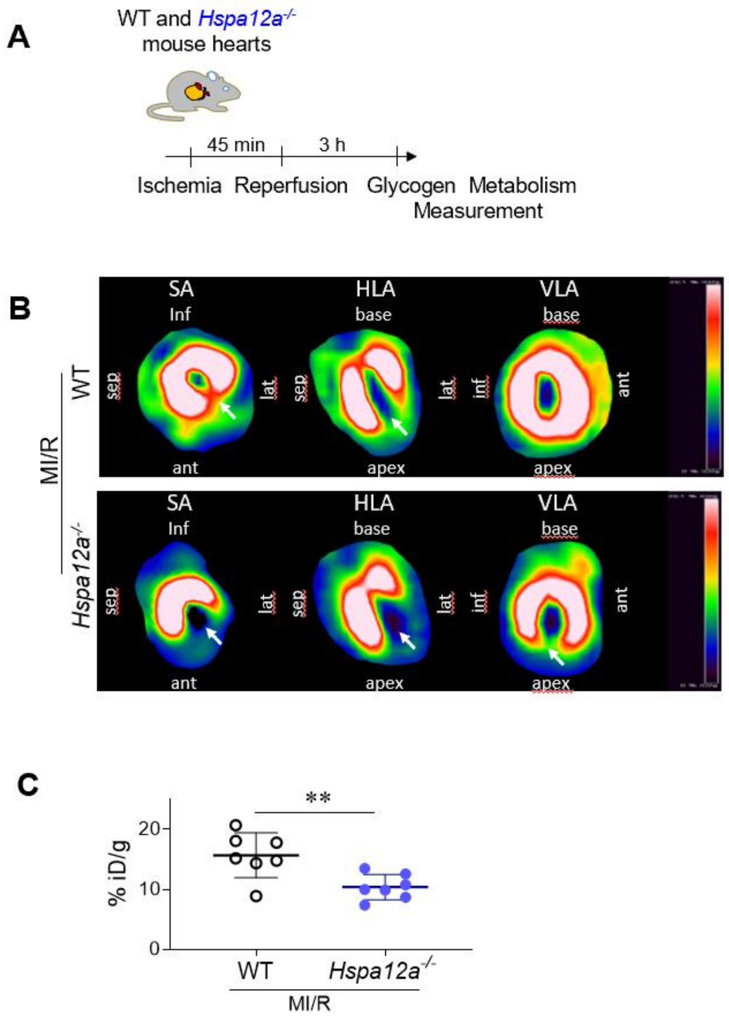

Figure 9. HSPA12A knockout in mice diminished ^{18}F-FDG uptake following MI/R.

A. Experimental protocol.

B. Glucose uptake is indicated by glucose analogue ^{18}F-FDG uptake using PET-CT. Representative PET-CT images of ^{18}F-FDG uptake at short, horizontal long and vertical long axis were shown in hearts following MI/R. Regions of ^{18}F-FDG uptake absence is indicated by the arrow.

C. Quantitative analysis of ^{18}F-FDG uptake activity by %ID/g after MI/R in heart. Data are means ± SD, $^{**}P < 0.01$ by Student's two-tailed unpaired *t* test. n = 7/group.

Abbreviations: WT, wild type mice; *Hspa12a*$^{-/-}$, HSPA12A knockout mice; MI/R, myocardial ischemia/reperfusion; SA, short axis; HLA, horizontal long axis; VLA, vertical long axis; sep, septum; lat, lateral wall; base, the base of the heart; apes, the apes of the heart; inf, inferior wall; ant, anterior wall; PET/CT, positron emission tomography/computed tomography; %ID/g, percent injected dose per gram of tissue.

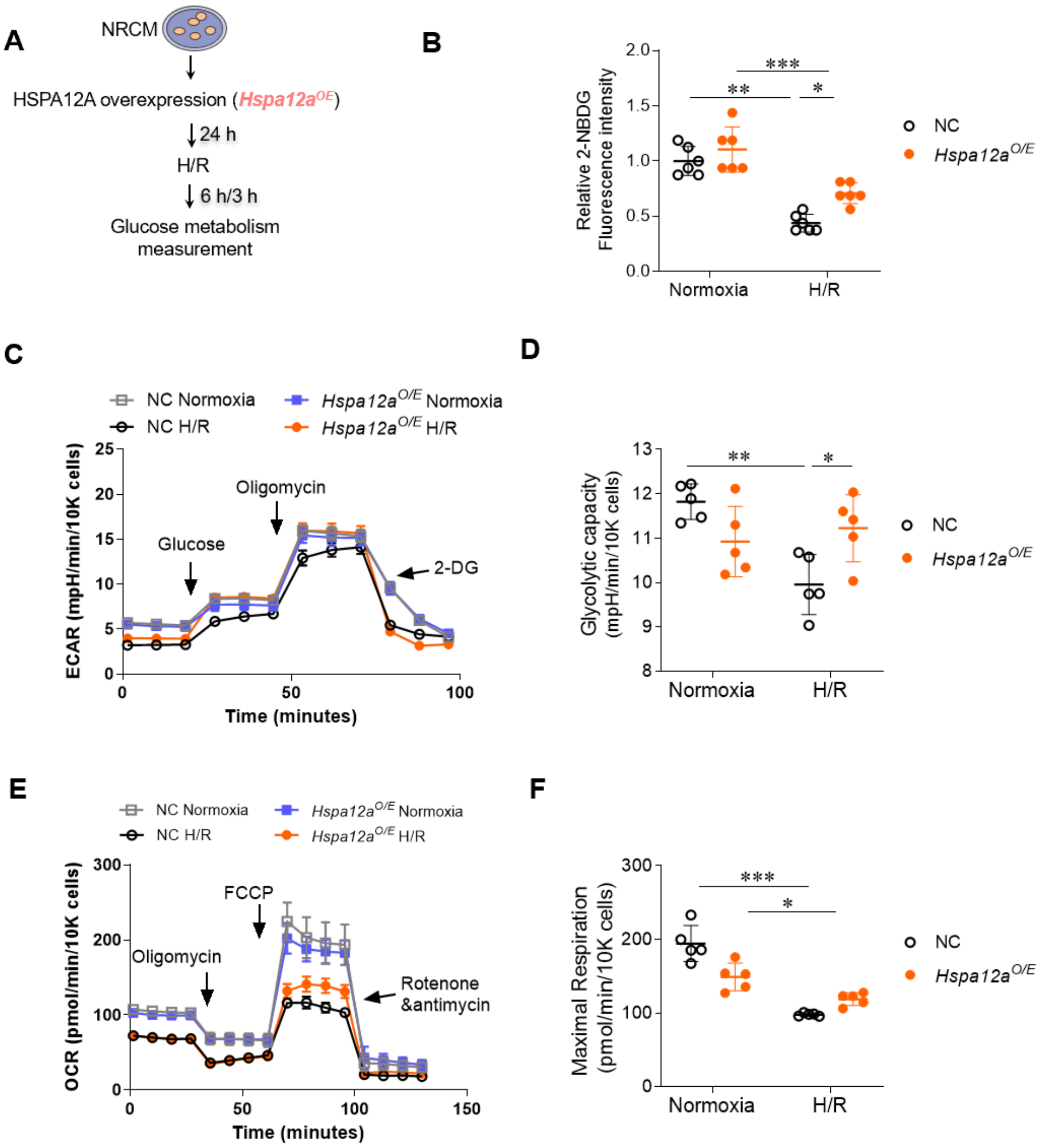

Figure 10. HSPA12A increased glucose uptake, glycolysis flux, but not affected glucose oxidation in H/R-treated cardiomyocytes.

A. Experimental protocol.

B. Glucose uptake is indicated by glucose analogue 2-NBDG (200 μM) uptake in NRCM. Data was expressed as averaged 2-NBDG fluorescence intensity in NRCM.

C. Glycolytic flux was assessed through Extracellular acidification rate (ECAR) in NRCM.

D. Quantitative analysis of glycolytic capacity which was represented maximum ECAR rate after oligomycin treatment.

E. Glucose oxidation was assessed through Extracellular acidification rate (OCR) in NRCM.

F. Quantitative analysis of the maximal respiration which was represented maximal mitochondrial OCR rate after FCCP treatment.

Data are means ± SD, ***$P < 0.001$, **$P < 0.01$, and *$P<0.05$ by two-way ANOVA followed by post hoc test. n = 6/group (**B**) and n = 5/group (**C-F**).

Abbreviations: NRCM, primary cardiomyocytes; 2-NBDG,2-[N-(7-Nitrobenz-2-oxa-1,3-diazol-4-yl)amino]-2-deoxy-d-glucose; H/R, hypoxia/reoxygenation; FCCP, fluoro-carbonyl cyanide phenylhydrazone.

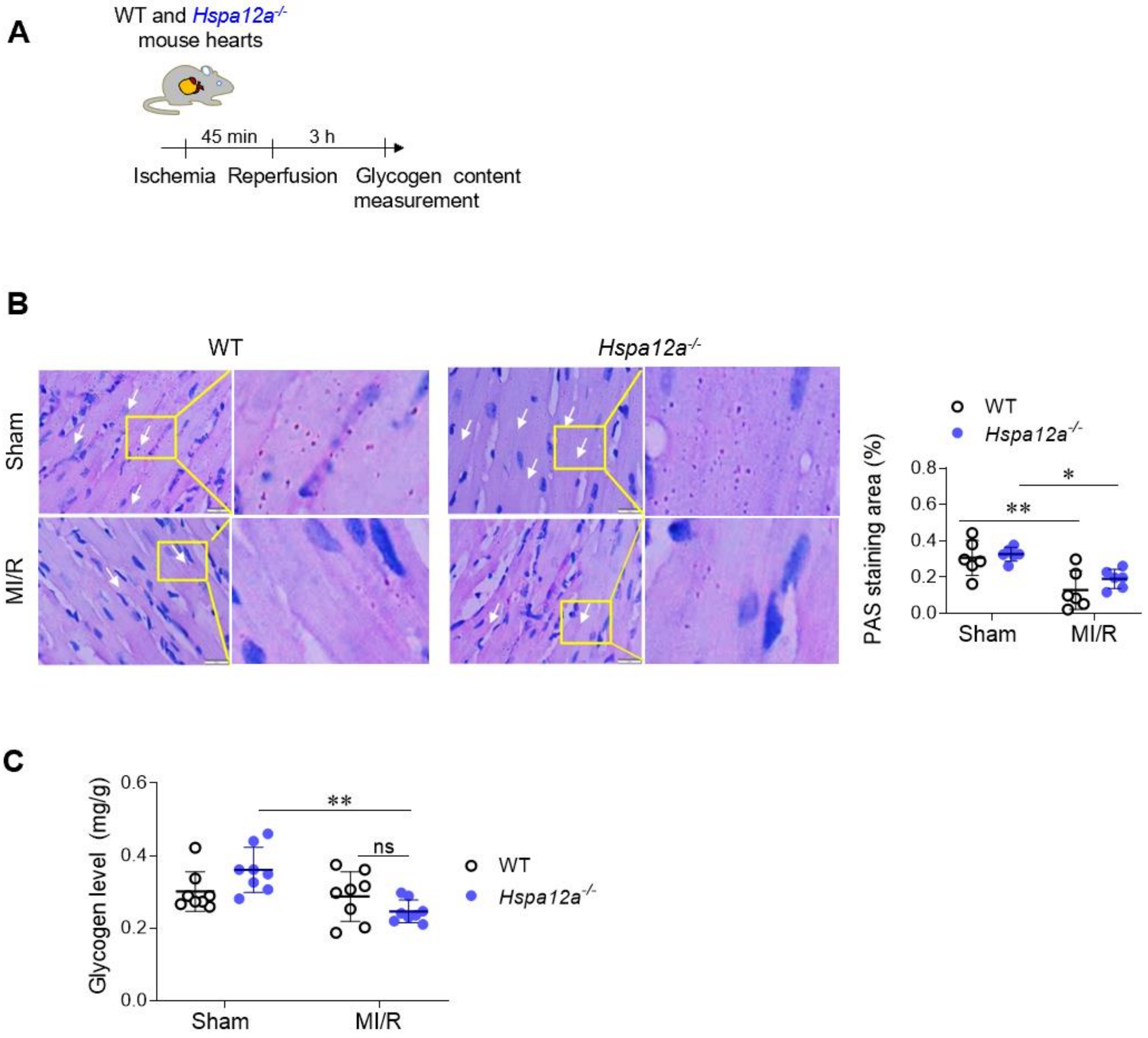

Figure 11. Effect of HSPA12A on glycogen content in heart after MI/R.

A. Experimental protocol.

B. Cardiac glycogen storage was examined by PAS staining on the paraffin-embedded sections. Glycogen staining is indicated by the arrows. Scale bar = 10 μm.

C. Glycogen contents were measured in myocardium after MI/R using the assay kit.

Data are means ± SD, $^{**}P < 0.01$ and $^{*}P < 0.05$ by two-way ANOVA followed by post hoc test. n = 6/group (**B**) and n = 8/group (**C**).

Abbreviations: PAS staining, Periodic Acid-Schiff staining.

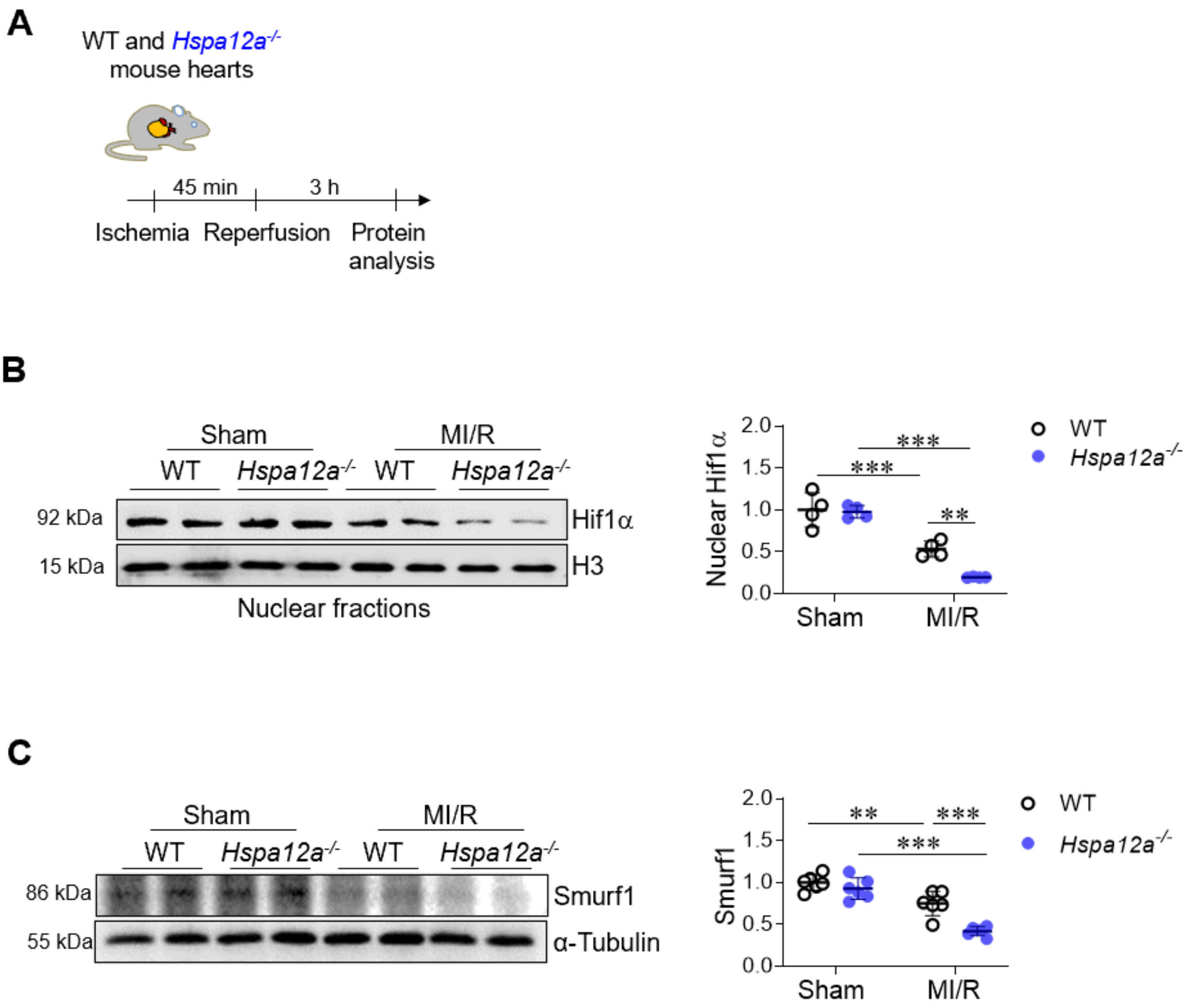

Figure 12. HSPA12A knockout decreased Smurf1-mediated H1F1α protein level in mouse hearts.

A. Experimental protocol.

B. Myocardial infarcts were collected after MI/R and nuclear protein fractions were prepared. The indicated gene expression was examined by immunoblotting analysis. The blots against Histone3 (H3) served as loading controls.

C. Smurf1 expression of whole protein lysates from heart tissue was examined by immunoblotting analysis, with α-Tubulin blots serving as loading controls.

Data are means ± SD, ****P* < 0.001 and ***P*<0.01 by two-way ANOVA followed by post hoc test. n = 4/group (**B**) and n = 6/group (**C**).

A

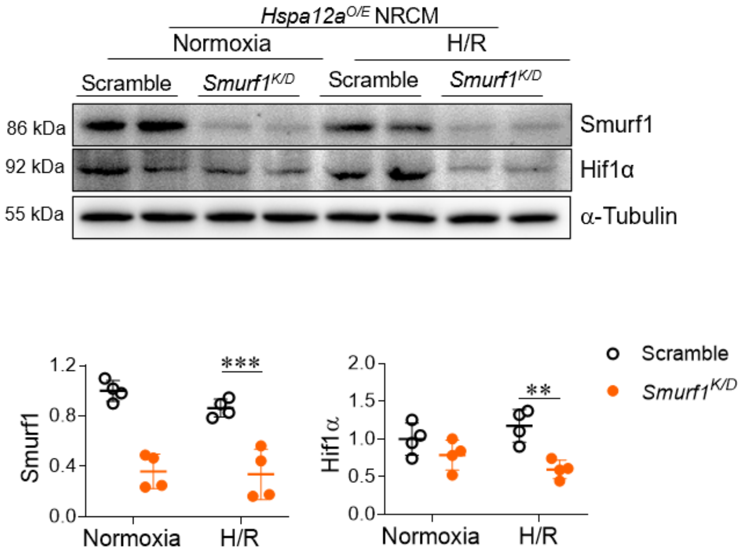

B

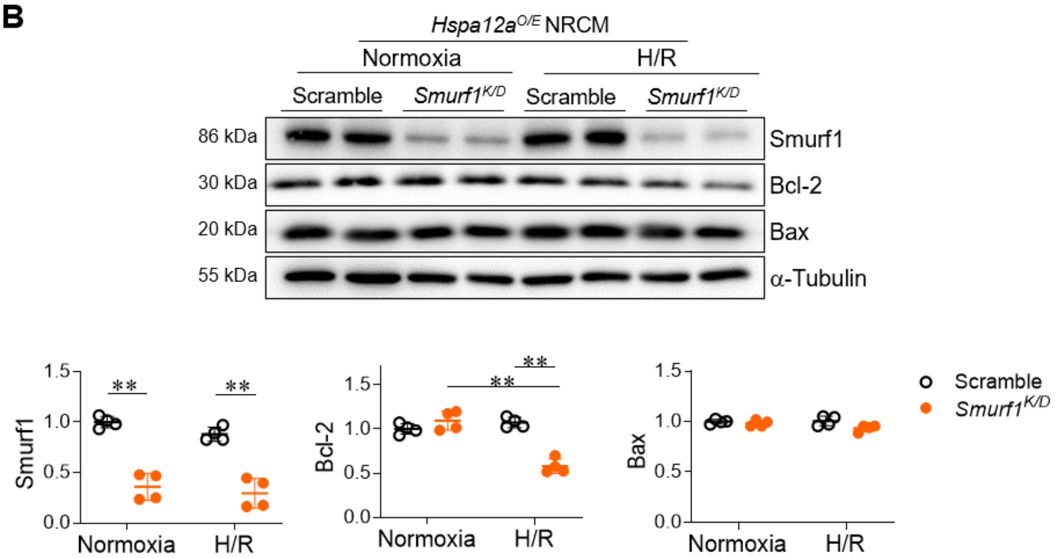

Figure 13. Smurf1 knockout reduced Hif1α and Bcl-2 protein expression in H/R-treated cardiomyocytes.

A. Effects of Smurf1 knockdown on Hif1α expression in *Hspa12a*$^{O/E}$ NRCM was examined using immunoblotting. The blots against α-Tubulin served as loading controls.

B. Protein expression of the indicated genes was examined by immunoblotting in NRCM. The blots against α-Tubulin served as loading controls.

Data are means ± SD, ****P* < 0.001 and ***P* < 0.01 by two-way ANOVA followed by post hoc test. n = 4/group.

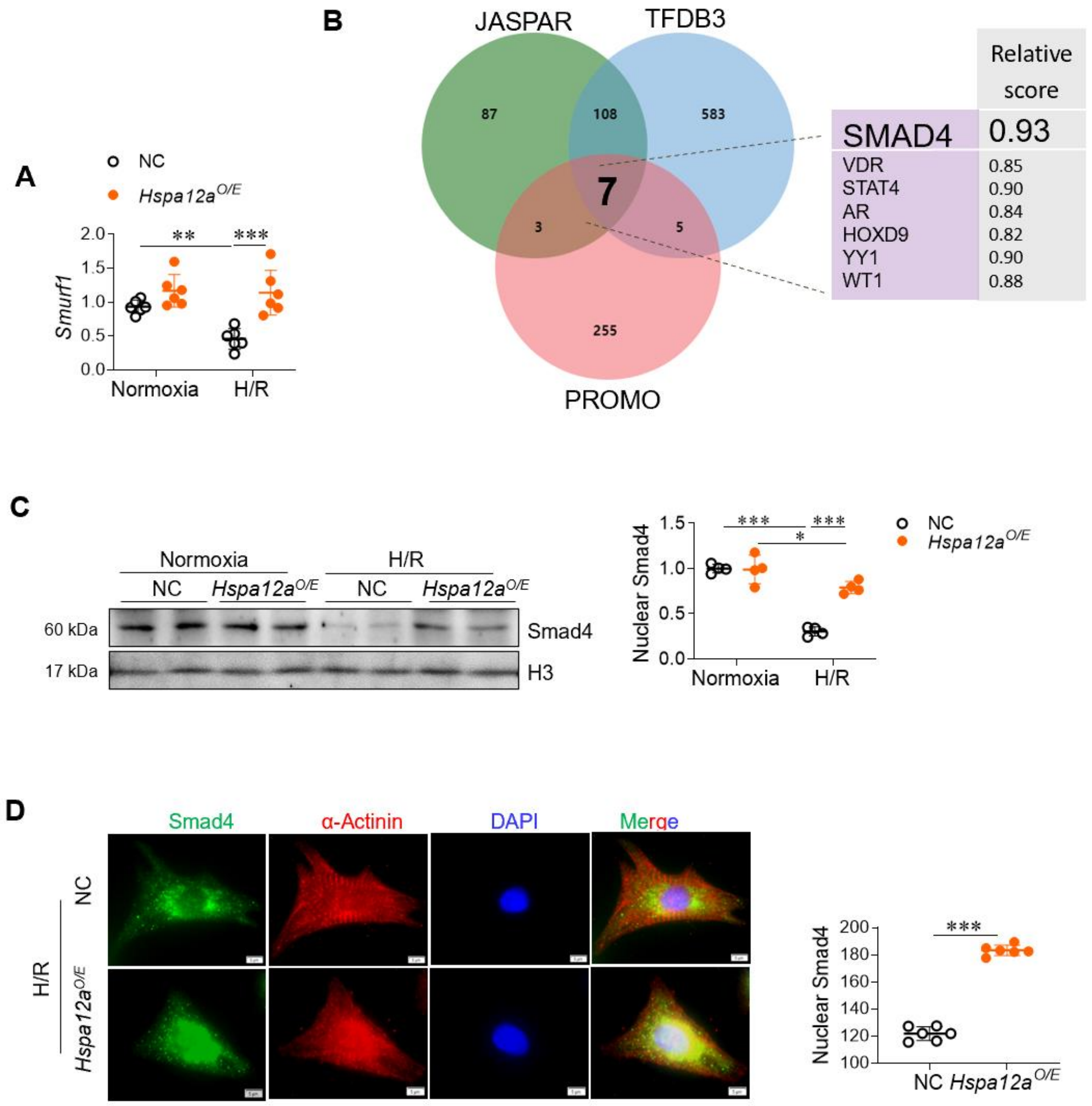

Figure 14. HSPA12A increased Smad4 expression and nuclear levels in H/R-treated cardiomyocytes

A. *Smurf1* mRNA levels were examined in NRCM. n = 6/group

B. Potential transcriptional factors for activating Smurf1 expression were predicted using PROMO, Animal TFDB and JASPAR database. Note that Smad4 has the highest score for activating Smurf1 transcription.

C. Smad4 protein levels were examined in nuclear fractions of NRCM. The blots against Histone3 (H3) served as loading controls. n = 4/group.

D. Immunofluorescence staining for Smad4 was examined in NRCM. Alpha-Actinin was used to stain cardiomyocytes and DAPI was used to counterstain the nuclei. Data was expressed as averaged Smad4 fluorescence intensity in each nuclei. Scale bar = 10 μm. n = 6/group.

Data are means ± SD, ****P* < 0.001, ***P* < 0.01 and **P*<0.05 by two-way ANOVA followed by post hoc test (**A** and **C**) or Student's two-tailed unpaired *t* test (**D**).

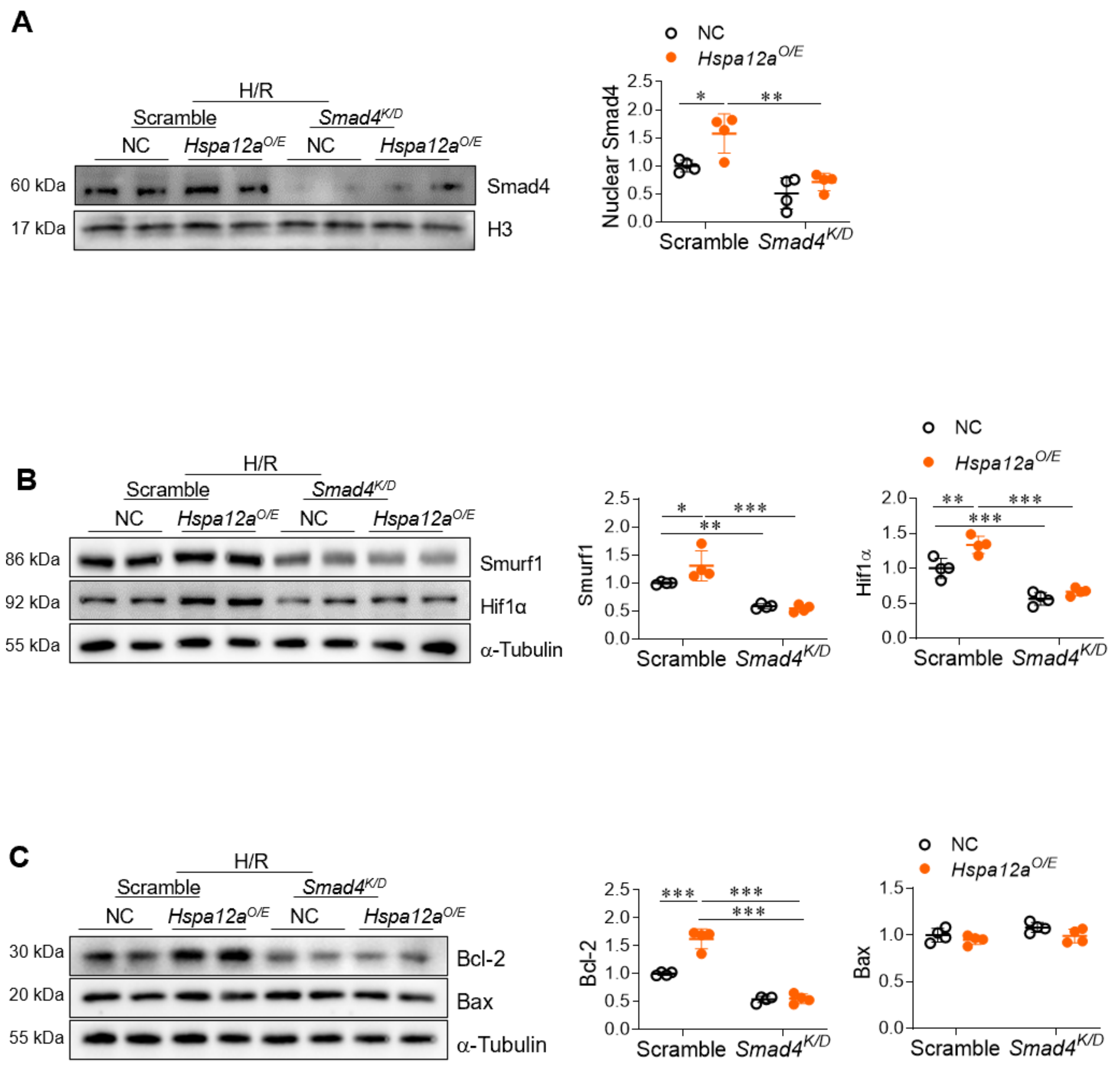

Figure 15. HSPA12A increased Smurf1-induced Hif1α and Bcl-2 expression in a Smad4-dependent manner.

A. Smad4 expression in nuclear fractions of NRCM was examined by immunoblotting analysis. The blots against Histone3 (H3) served as loading controls.

B. Effects of Smad4 knockdown on Smurf1 and Hif1α expression in NRCM after H/R was examined by immunoblotting analysis. The blots against α-Tubulin served as loading controls.

C. Bcl-2 and Bax expression levels were examined using immunoblotting analysis. The blots against α-Tubulin served as loading controls.

Data are means ± SD, ****P* < 0.001, ***P* < 0.01and **P*<0.05 by two-way ANOVA followed by post hoc test. n = 4/group.

Table 1. Predicted ubiquitin ligases/deubiquitinases of HSPA12A binding

Gene	Full name
Smurf1	SMAD Specific E3 Ubiquitin Protein Ligase 1
SYVN1	Synoviolin 1
CBL	Cbl Proto-Oncogene
DTX1	Deltex E3 Ubiquitin Ligase 1
TRIM33	Tripartite Motif Containing 33
STUB1	STIP1 Homology And U-Box Containing Protein 1
CBLC	Cbl Proto-Oncogene C
PHIP	Pleckstrin Homology Domain Interacting Protein
SOCS7	Suppressor Of Cytokine Signaling 7
ERCC8	ERCC Excision Repair 8, CSA Ubiquitin Ligase Complex Subunit
PML	PML Nuclear Body Scaffold
ZEB2	Zinc Finger E-Box Binding Homeobox 2
MARCH9	Membrane Associated Ring-CH-Type Finger 9
TTC3	Tetratricopeptide Repeat Domain 3
BMI1	BMI1 Proto-Oncogene, Polycomb Ring Finger
MIB1	MIB E3 Ubiquitin Protein Ligase 1
SIAH1	Siah E3 Ubiquitin Protein Ligase 1
AMFR	Autocrine Motility Factor Receptor
SOCS1	Suppressor Of Cytokine Signaling 1

Table 2. Antibodies used in the experiments

Antibody	Source	Company	Catalog No.
anti-HSPA12A	Rabbit	Abcam	ab200838
anti-GLUT4	Rabbit	Abcam	ab33780
anti-HK II	Rabbit	Cell Signaling	#2867
anti-LDHA	Rabbit	Abcam	ab101562
anti-GLUT1	Rabbit	Proteintech	21829-1-AP
anti-α-Actinin	Mouse	Sigma	A7811
anti-Bax	Rabbit	Cell Signaling	2772S
anti-Hif1α	Rabbit	Abcam	ab179483
anti-Bcl-2	Rabbit	Cell Signaling	3498S
anti-Smurf1	Mouse	Santa Cruz Biotechnology	sc-100616
anti-VHL	Rabbit	Santa Cruz Biotechnology	sc-135651
anti-Flag	Mouse	Sigma-Aldrich	F1804
anti-IgG	Mouse	Biotechnology	sc-2025
anti-α-Tubulin	Mouse	proteintech	66031-1-1g
anti-Histone-H3	Rabbit	Proteintech	17168-1-AP
anti-Smad4	Rabbit	Bioworld	BS2050
Cy3-AffiniPure Goat Anti-Rabbit IgG		Jackson lmmunoResearch	111-165-003
Cy3-AffiniPure Goat Anti-Mouse IgG		Jackson lmmunoResearch	115-165-003

Table 3. SiRNA sequence used in the experiments

Species	Gene name		Sequence (5'-3')
Rat	*Hspa12a*	Sense	CCU GGG UUG ACC UAA UGA UTT
		Antisense	AUC AUU AGG UCA ACC CAG GTT
Rat	*Sumrf1*	Sense	GAACGAAGGAACGGUAUAUTT
		Antisense	AUAUACCGUUCCUUCGUUCTT
Rat	*Smad4*	Sense	CAGCUACUUACCACCAUAATT
		Antisense	UUAUGGUGGUAAGUAGCUGTT

Table 4. Primers used in the experiments

Species	Gene name		Sequence (5'-3')
Rat	*Hif1α*	Sense	CCGCAGTGTGGCTACAAGAA
		Antisense	GATGAGGAATGGGTTCACAAATC
Rat	*Smurf1*	Sense	AGTTCGTGGCCAAATAGTGG
		Antisense	GTTCCTTCGTTCTCCAGCAG
Rat	*36b4*	Sense	TCCTGAGCGATGTGCAGCTGATAA
		Antisense	GCCATTGTCAAACACCTGCTGGAT
