# Supplementary material for: HSPA12A maintains aerobic glycolytic homeostasis and Histone3 lactylation in cardiomyocytes to attenuate myocardial ischemia/reperfusion injury
Source: JCI Insight. 2024 Feb 29;9(7):e169125. doi: 10.1172/jci.insight.169125 (PMC11128201; doi:10.1172/jci.insight.169125)

Full unedited gel for Figure 1

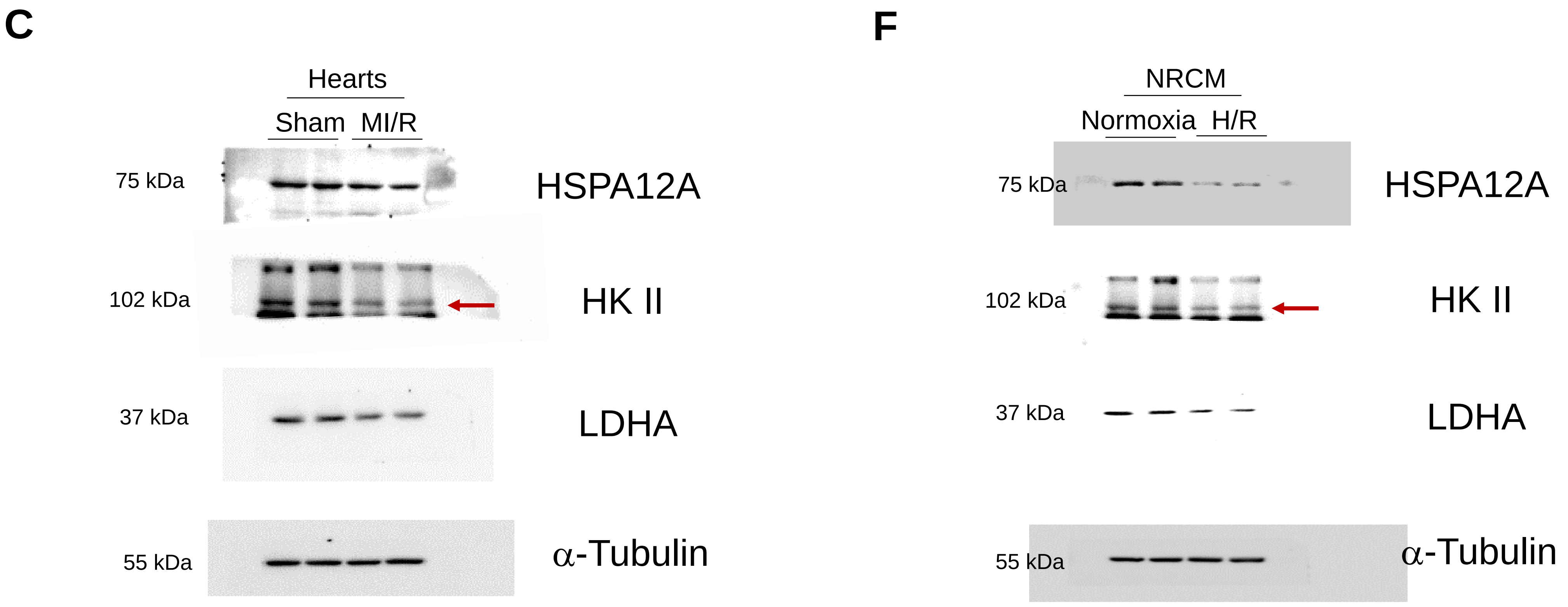

Full unedited gel for Figure 2

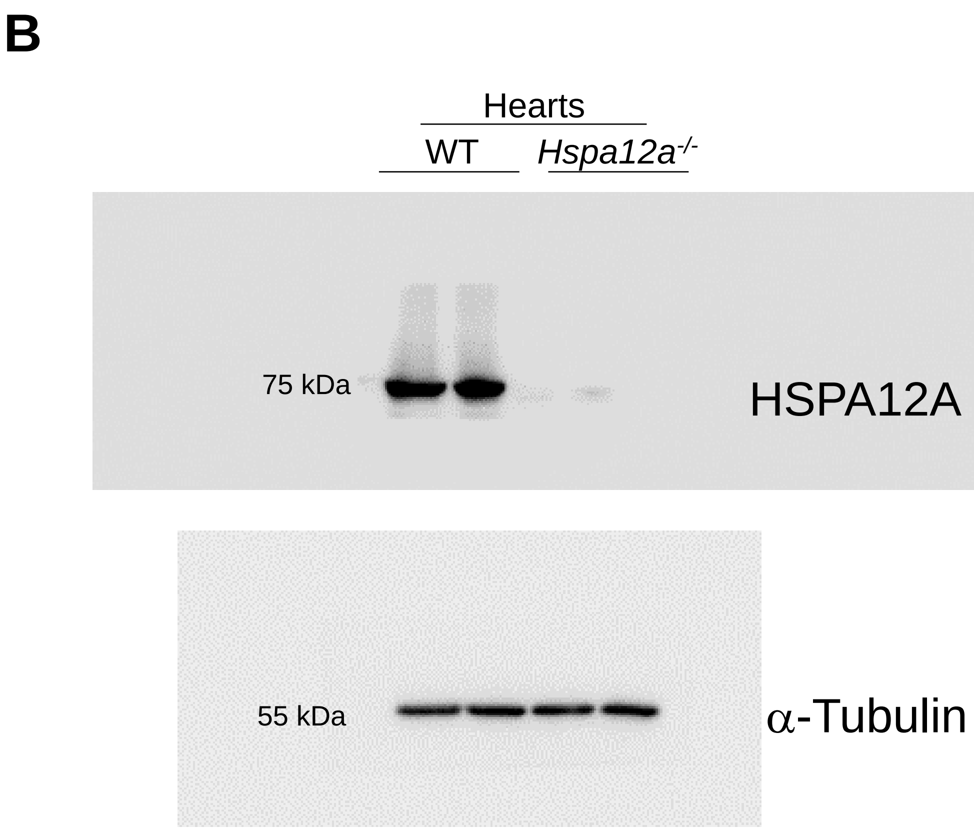

Full unedited gel for Figure 3

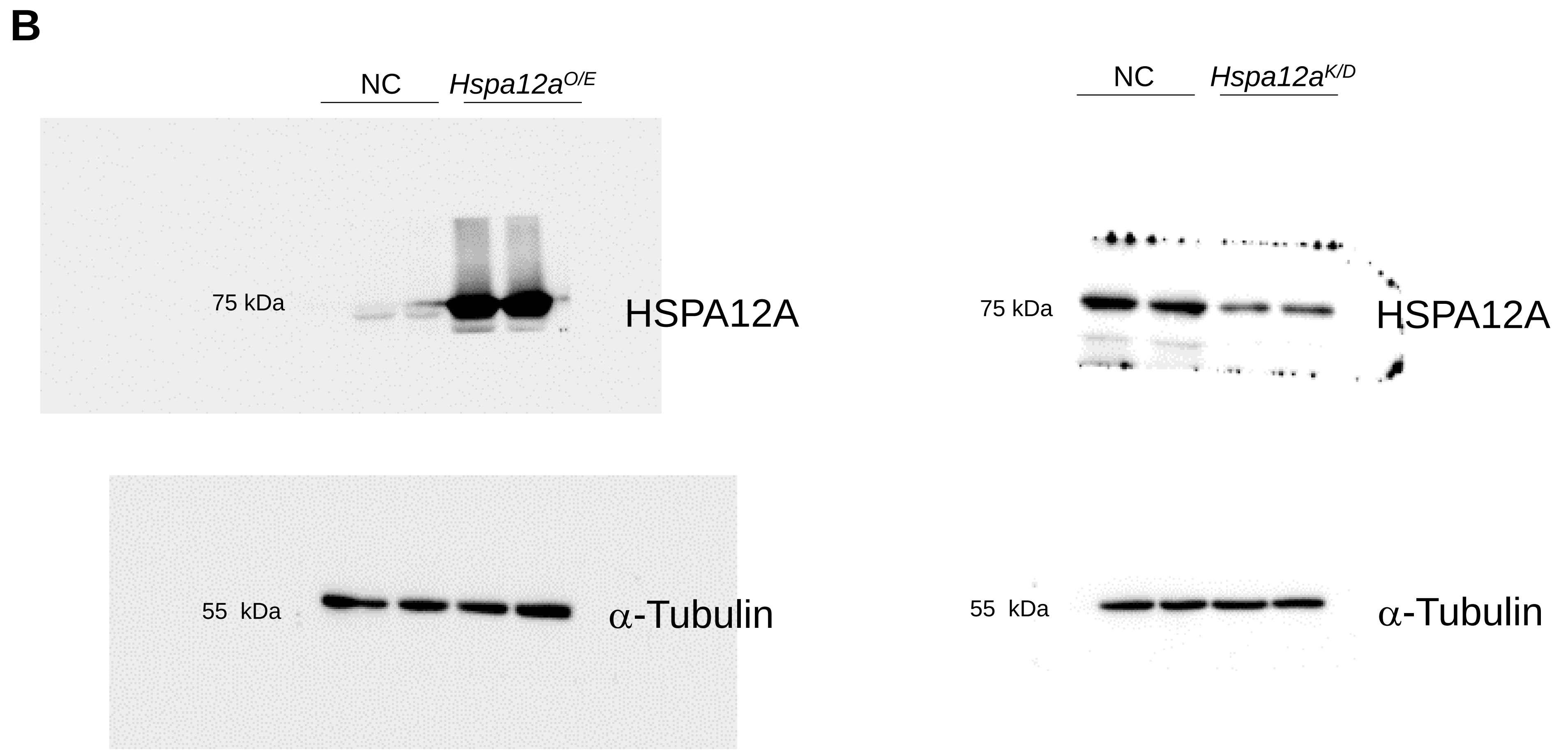

Full unedited gel for Figure 4

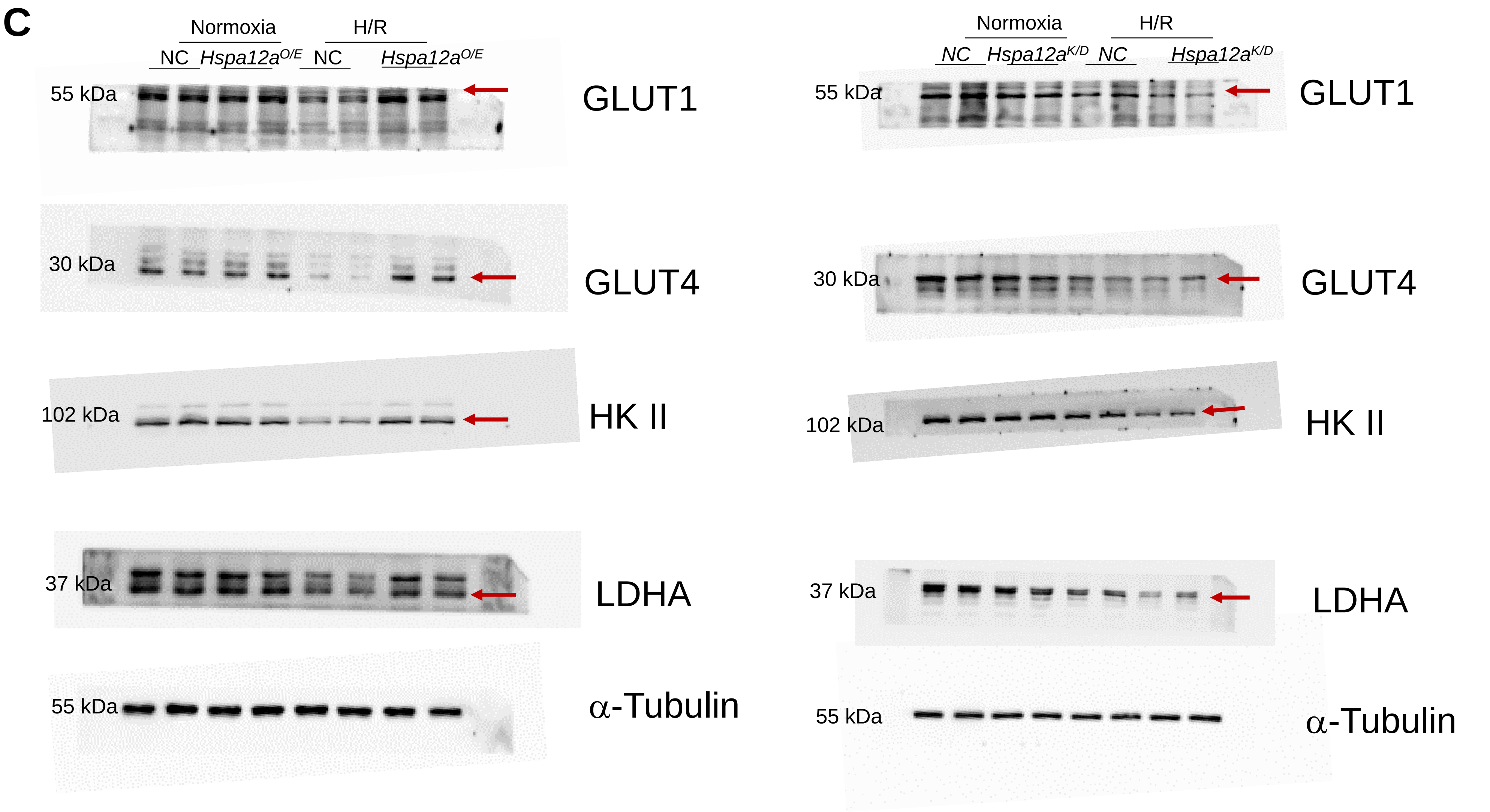

Full unedited gel for Figure 5

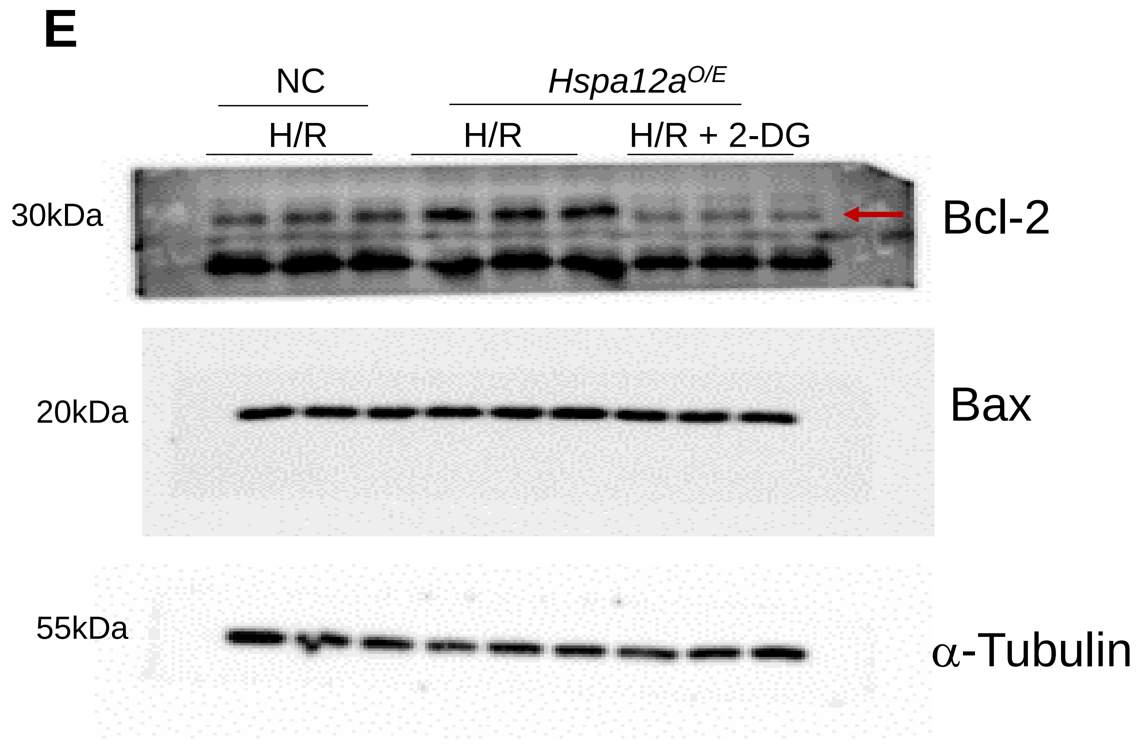

Full unedited gel for Figure 6

B

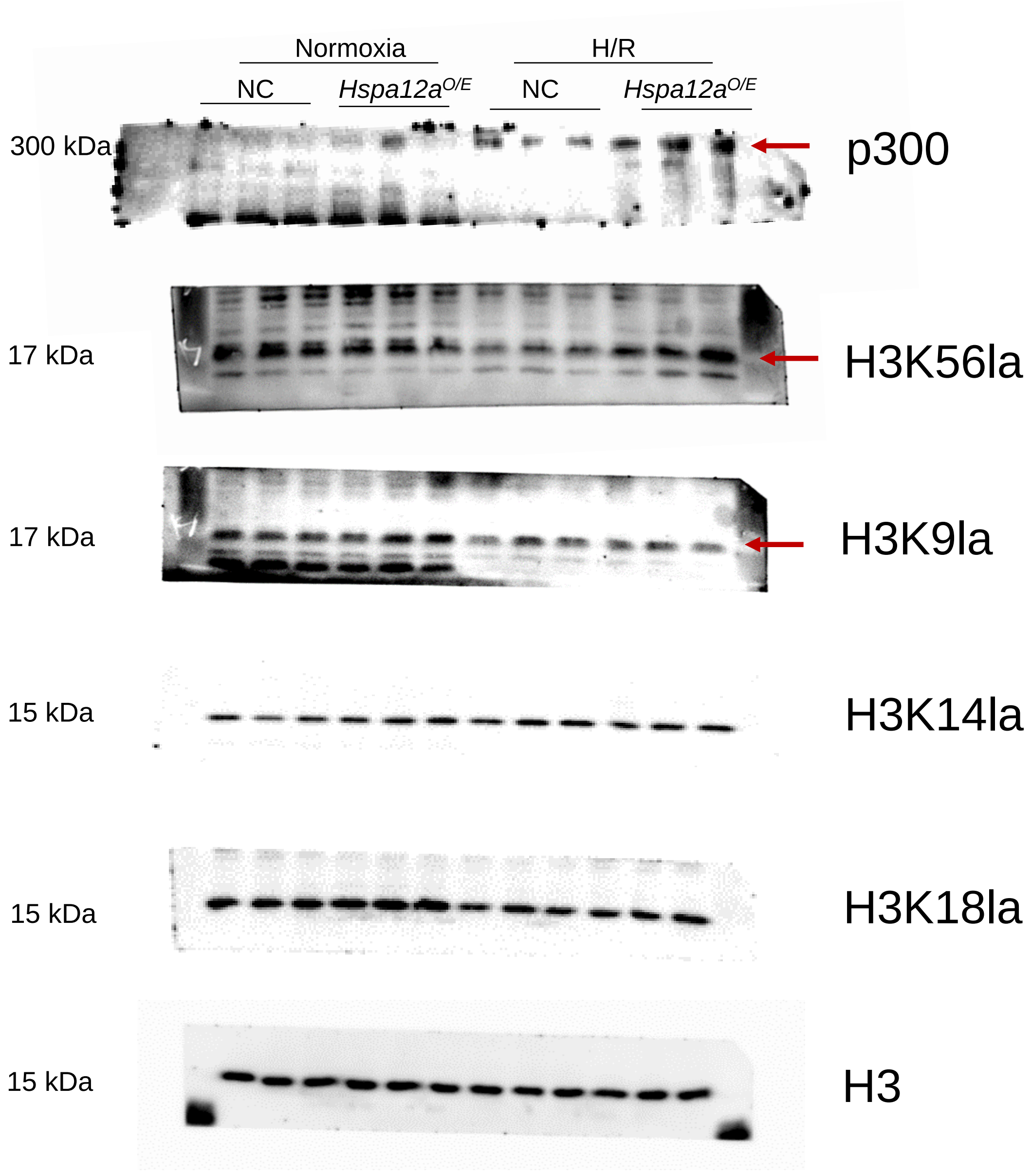

Full unedited gel for Figure 7

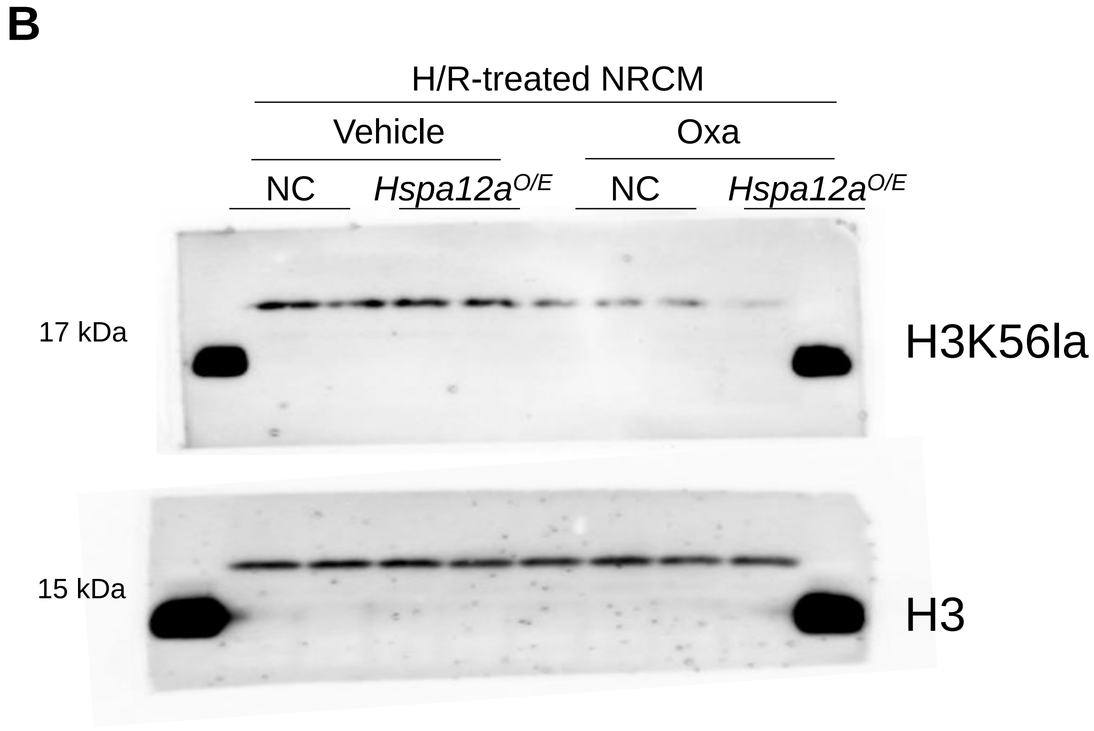

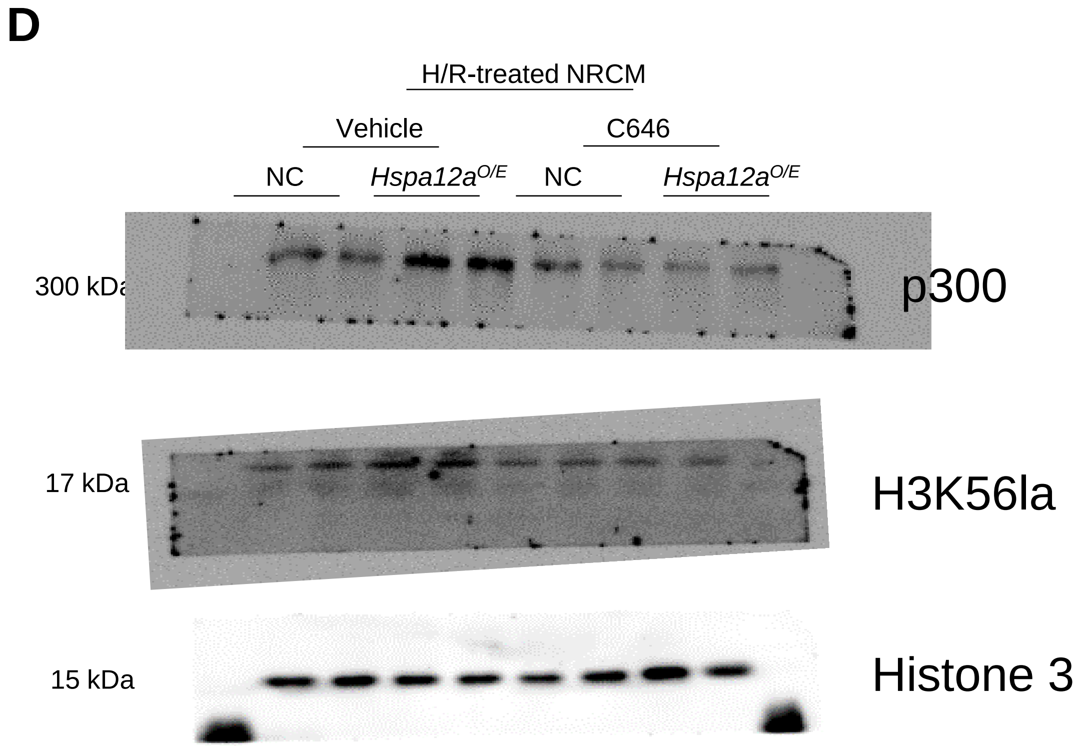

Full unedited gel for Figure 8

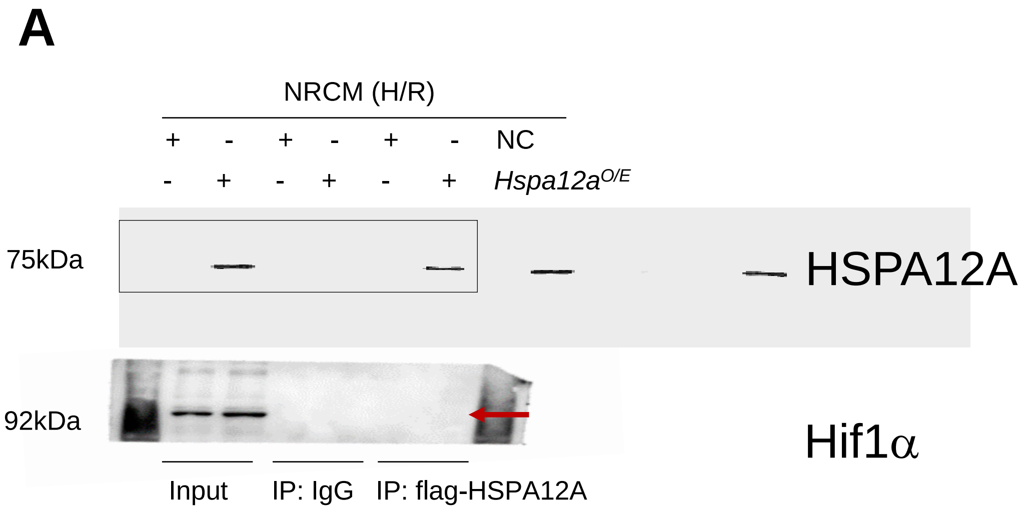

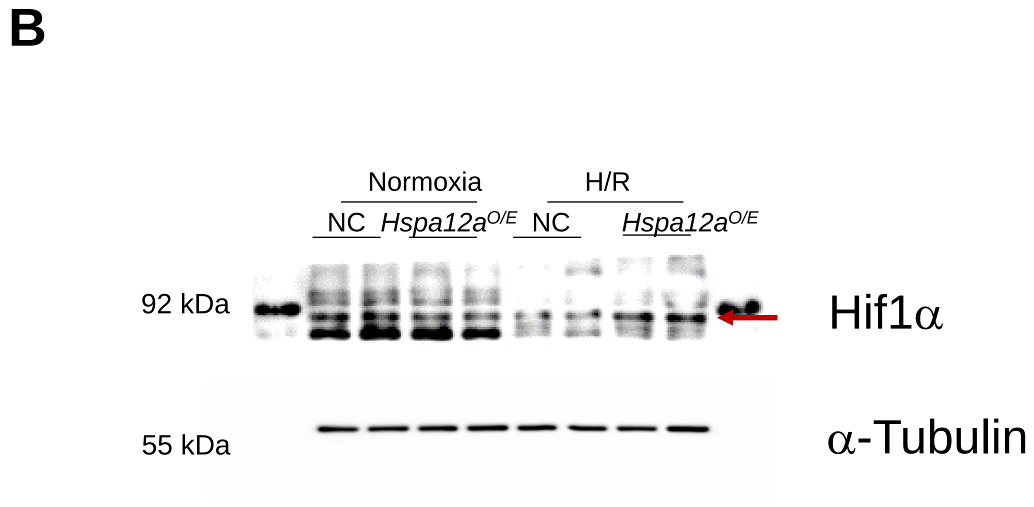

Full unedited gel for Figure 8

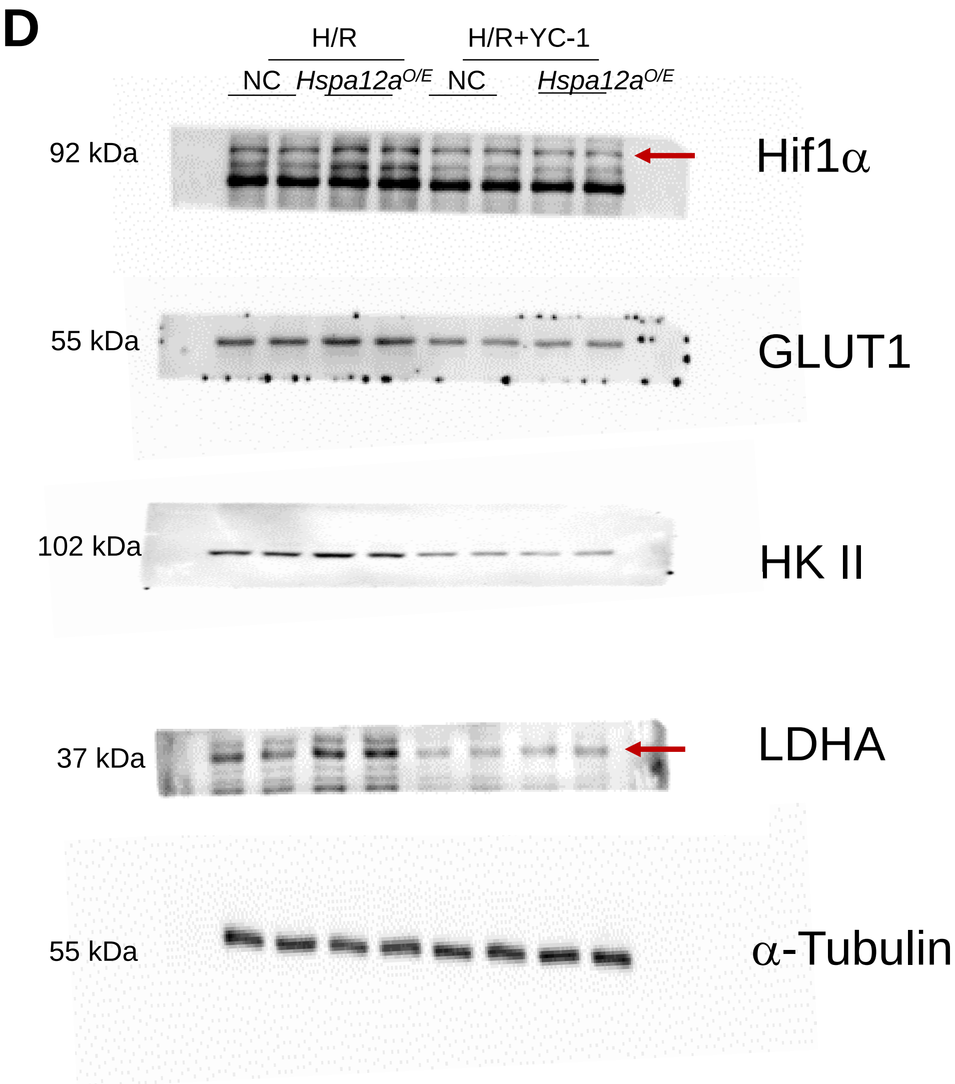

Full unedited gel for Figure 9

B

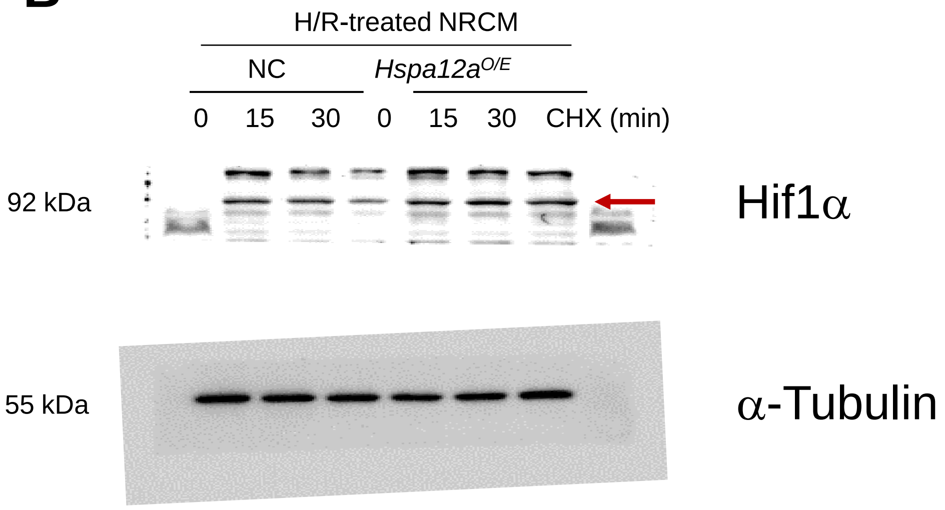

C

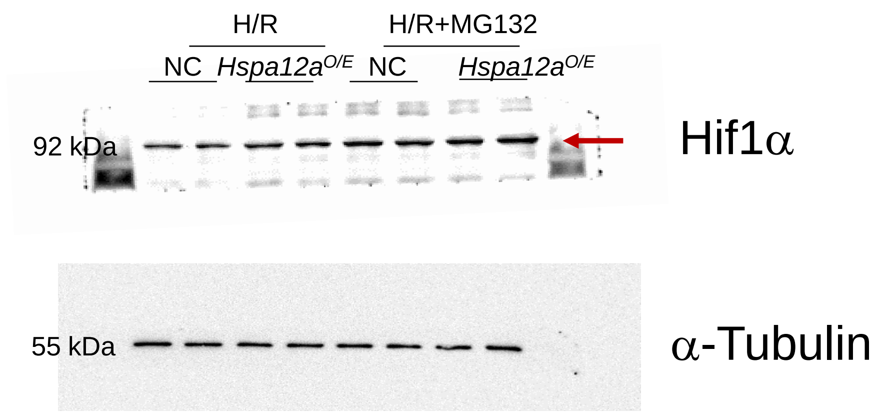

Full unedited gel for Figure 10

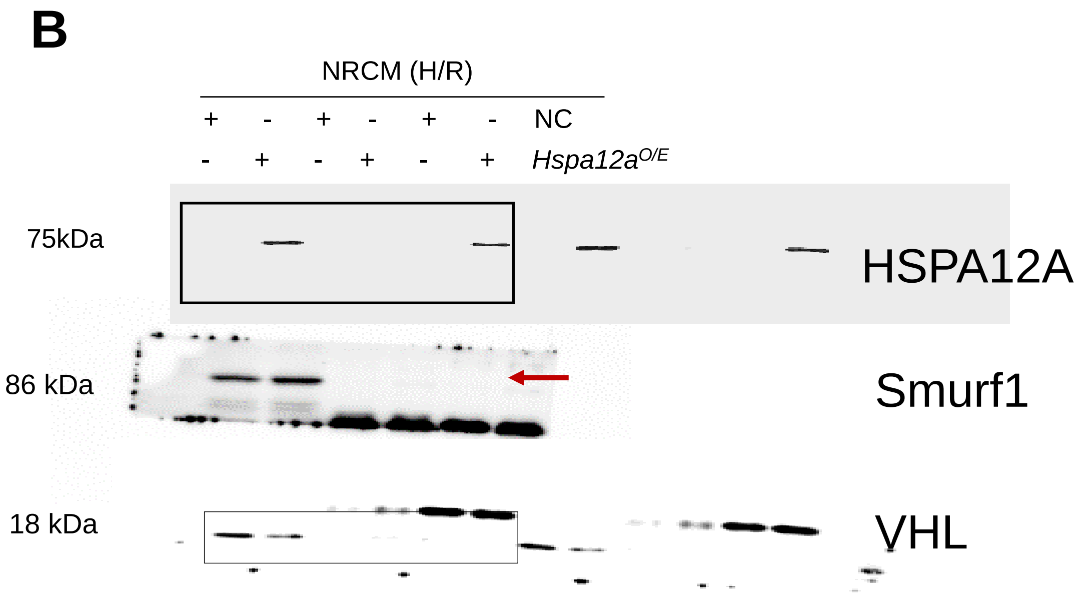

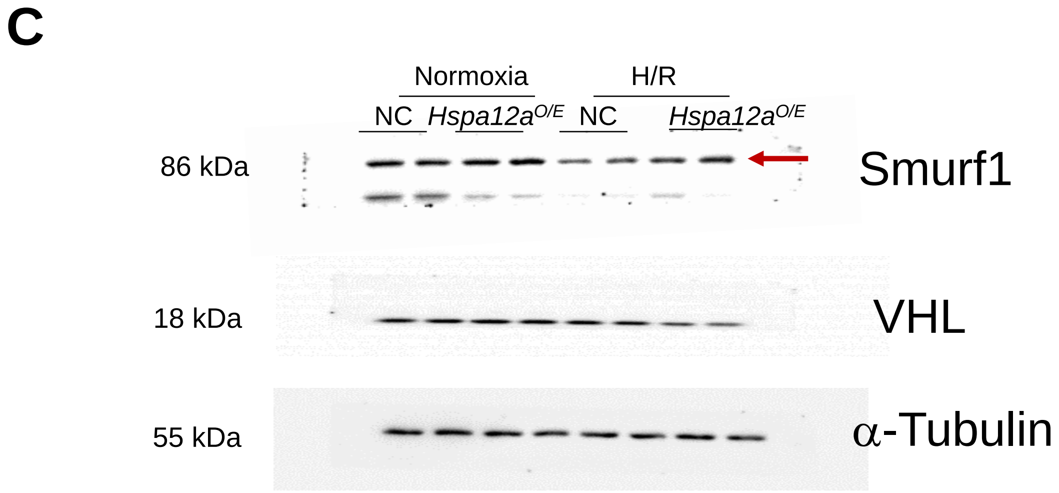

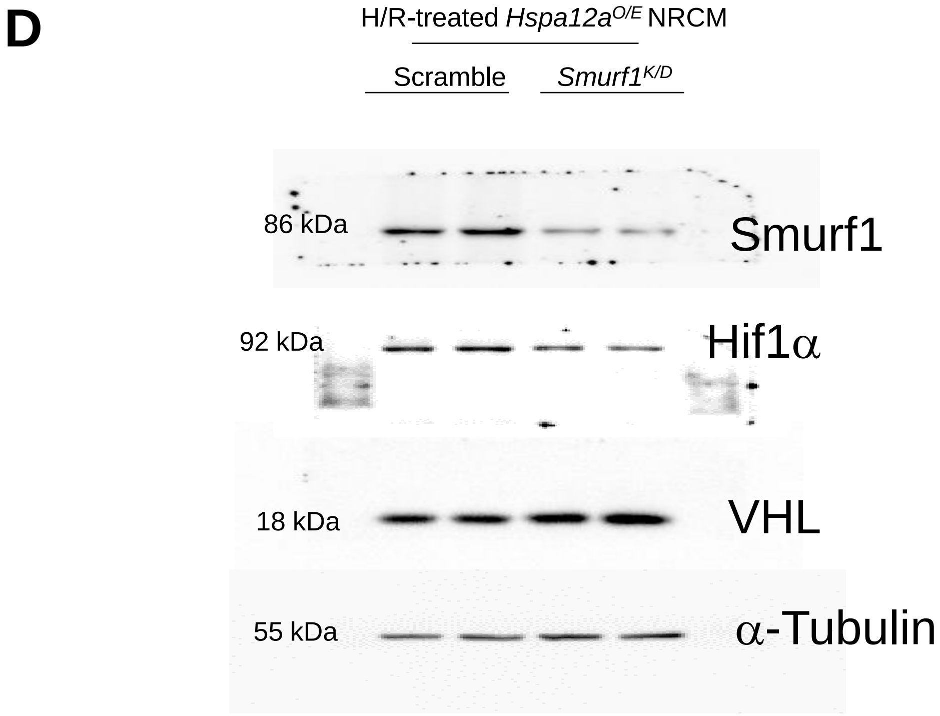

Full unedited gel for Supplement Figure S1

A

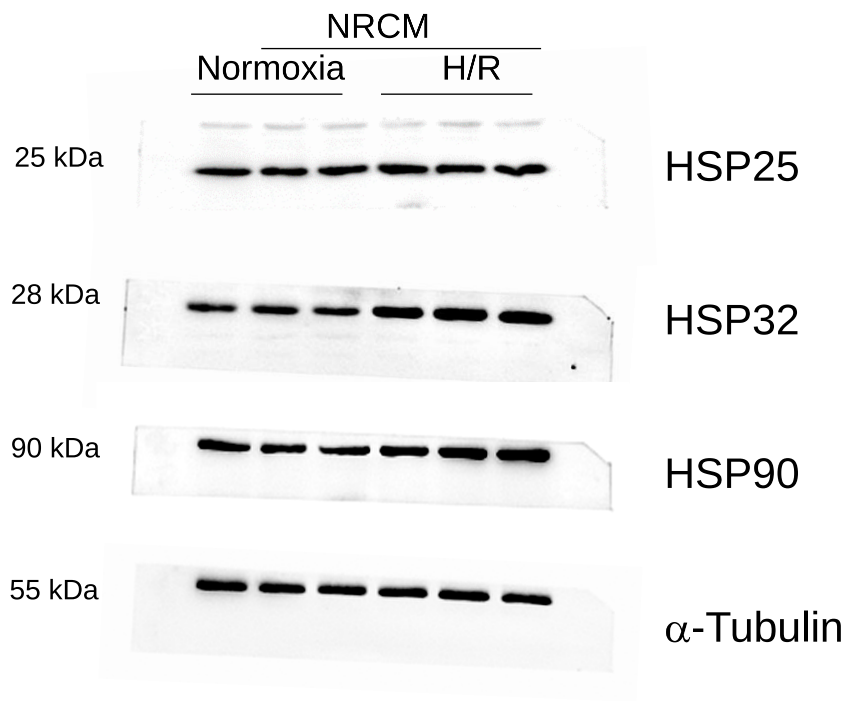

B

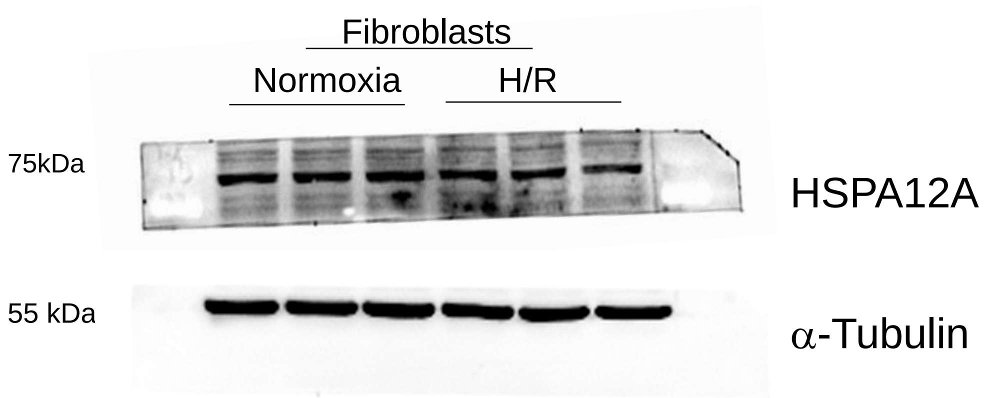

Full unedited gel for Supplement Figure S4

B

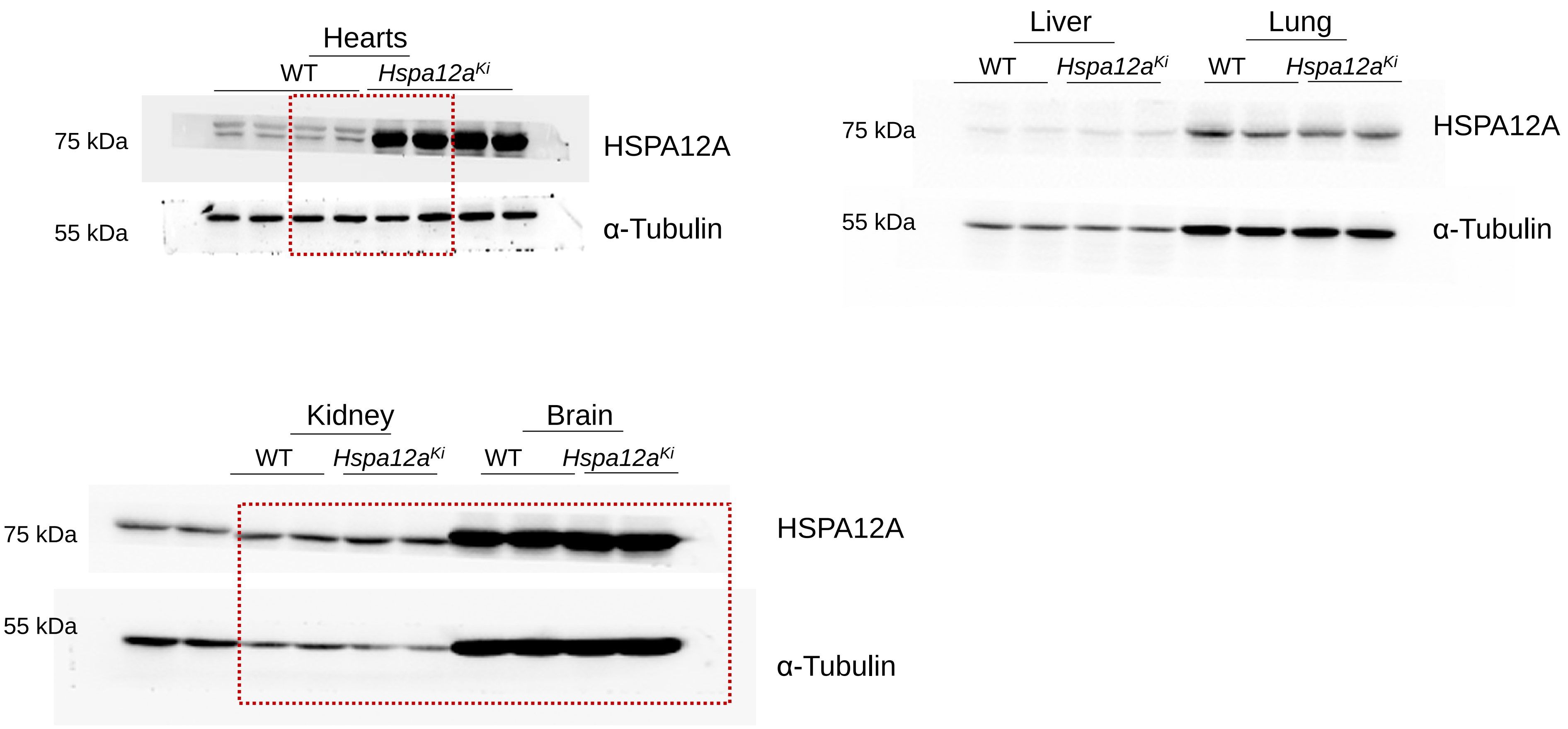

Full unedited gel for Supplement Figure S6

C

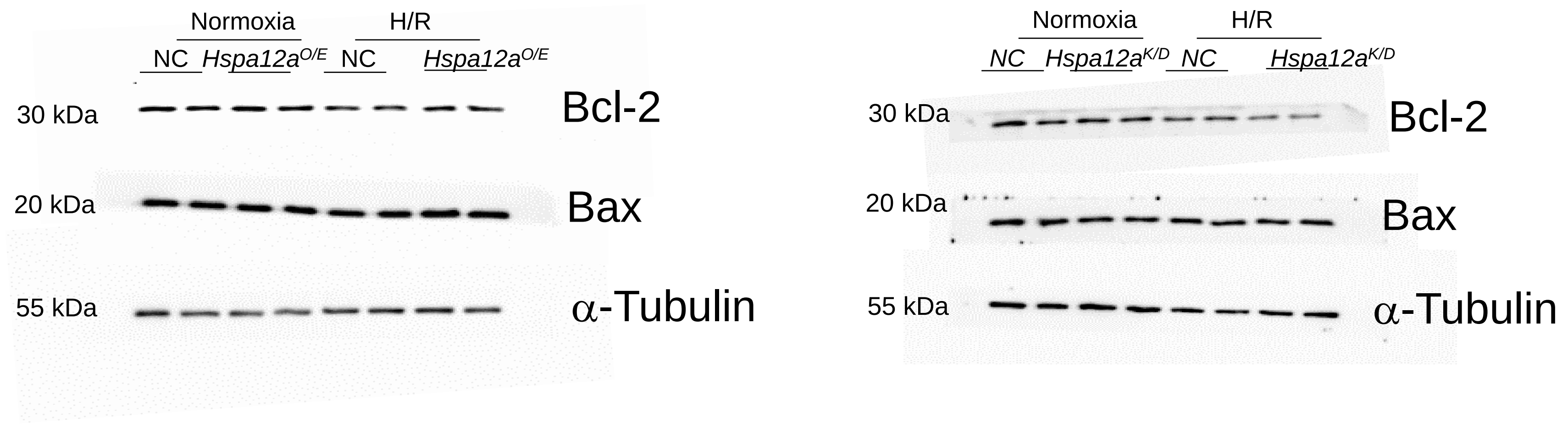

Full unedited gel for Supplement Figure S7

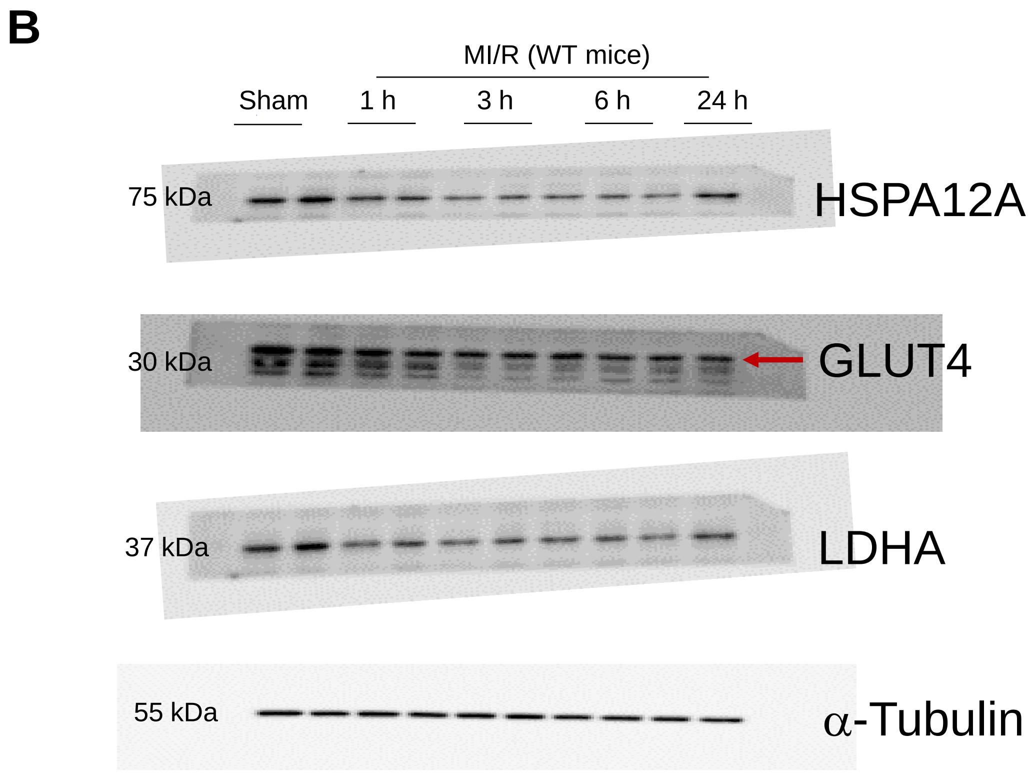

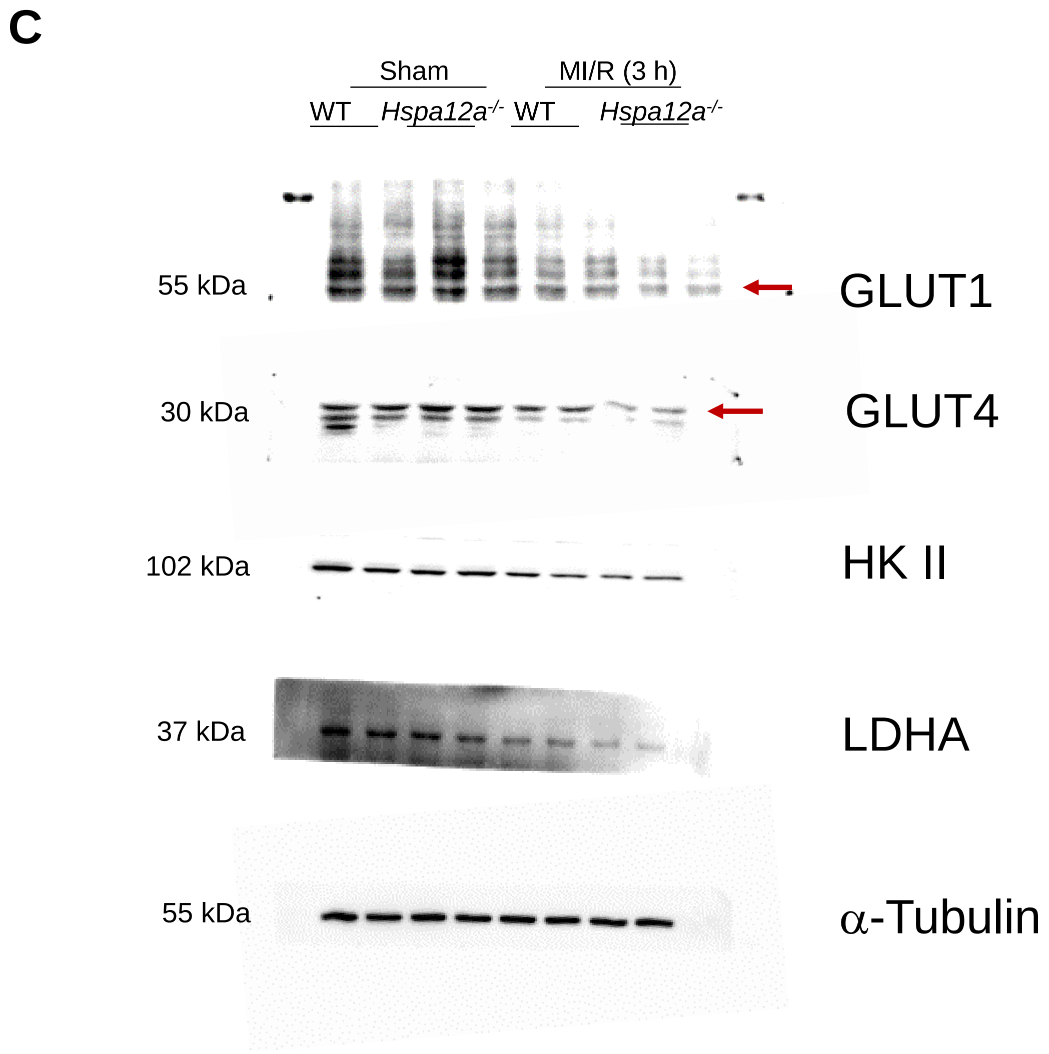

Full unedited gel for Supplement Figure S8

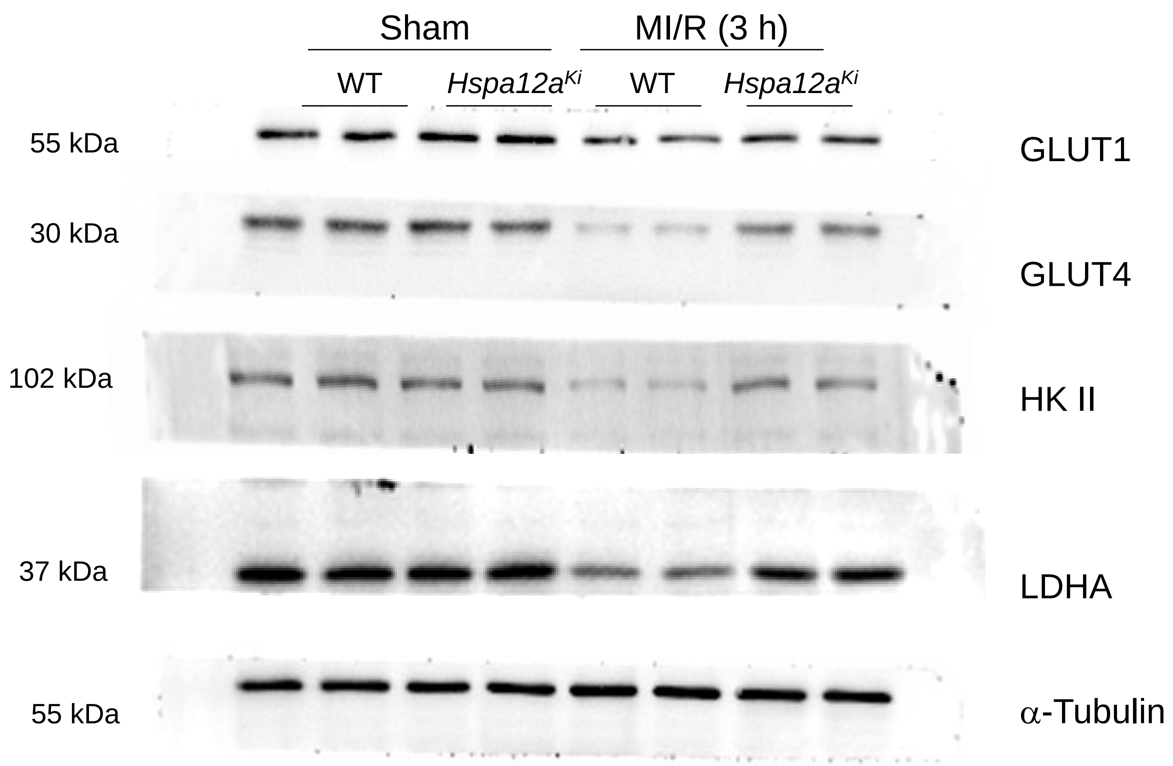

Full unedited gel for Supplement Figure S12

B

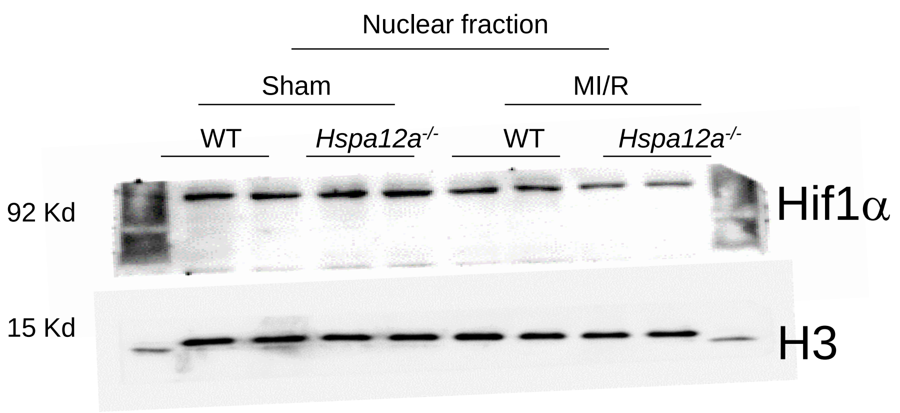

C

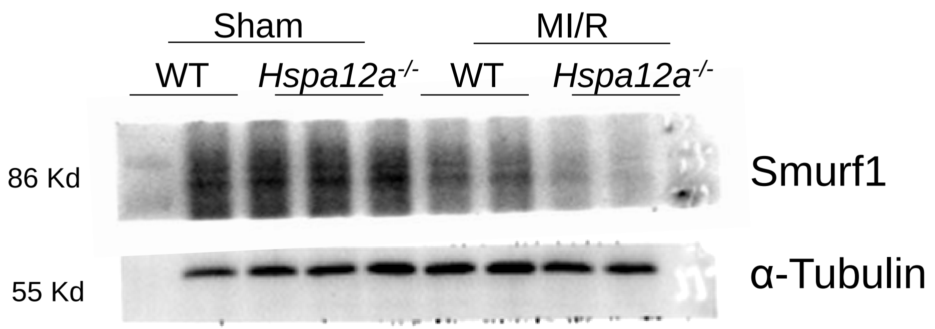

Full unedited gel for Supplement Figure S13

A

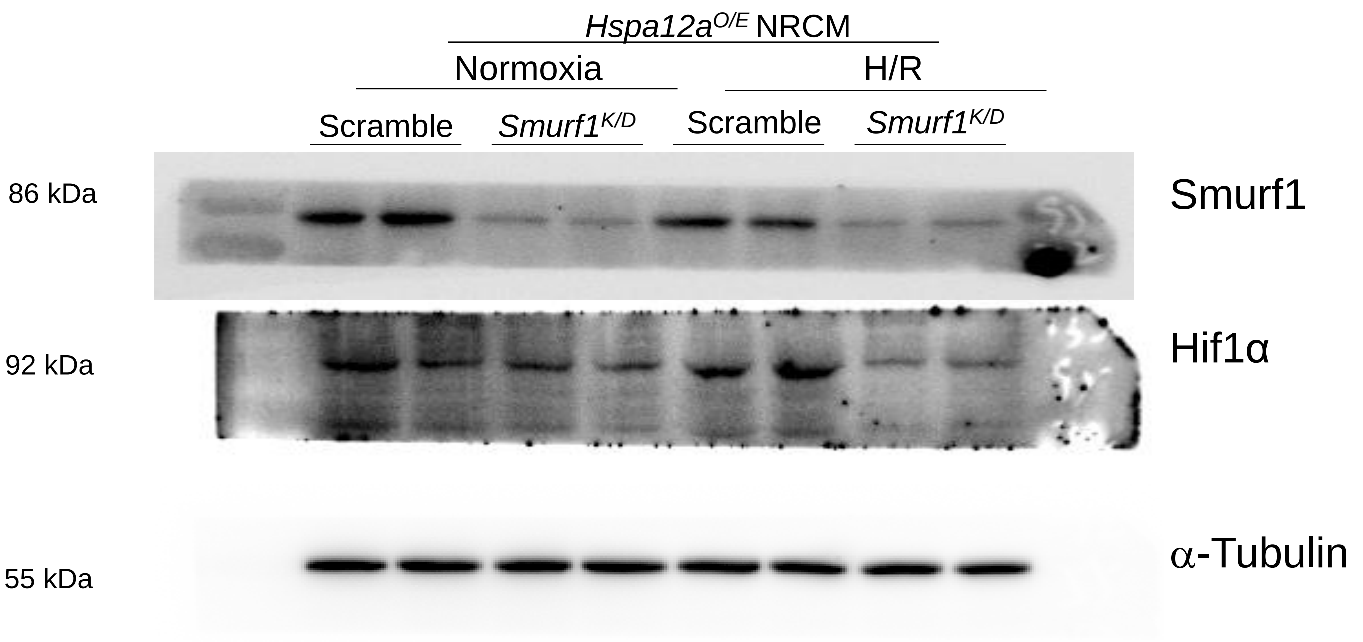

Full unedited gel for Supplement Figure S13

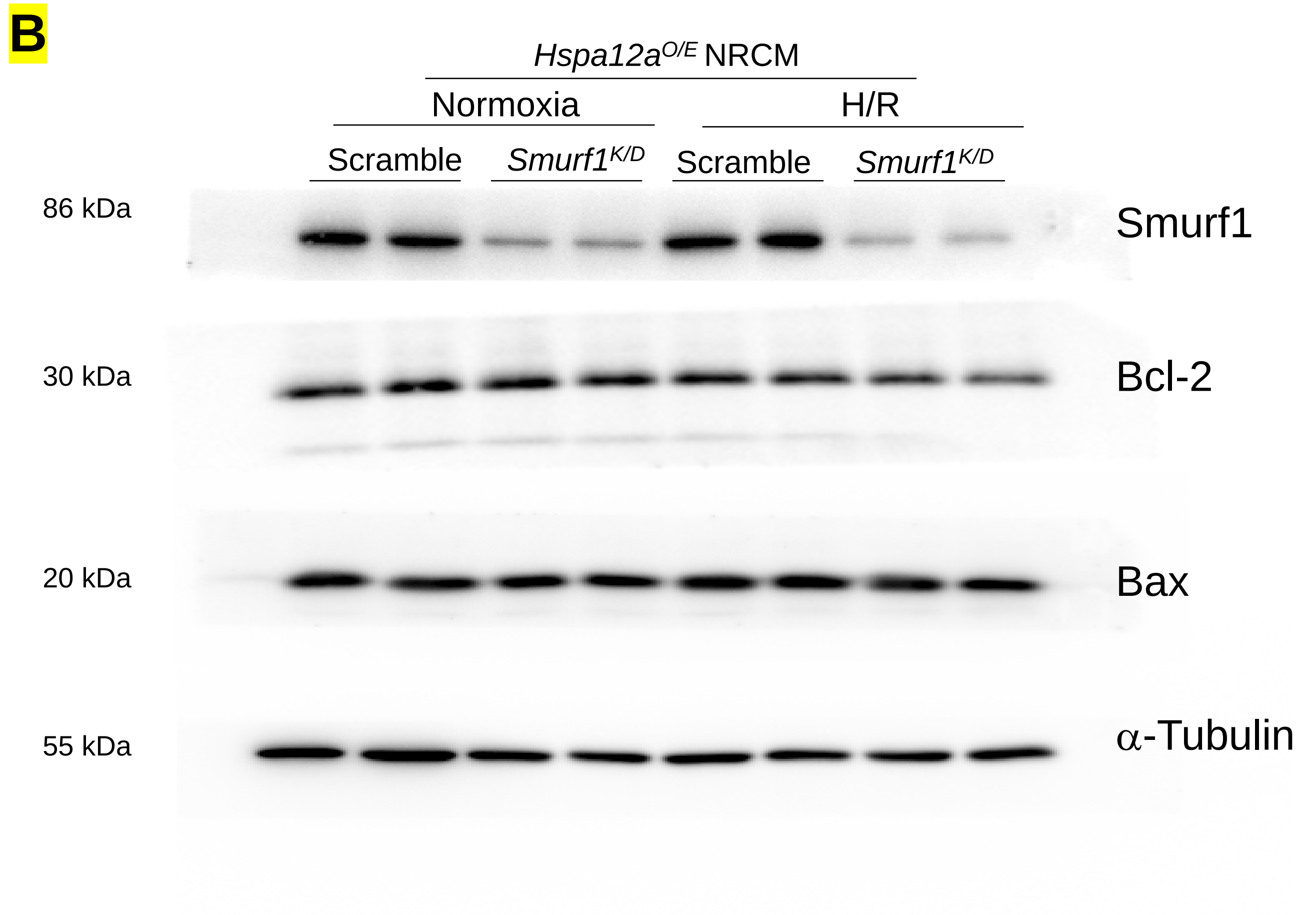

Full unedited gel for Supplement Figure S14

C

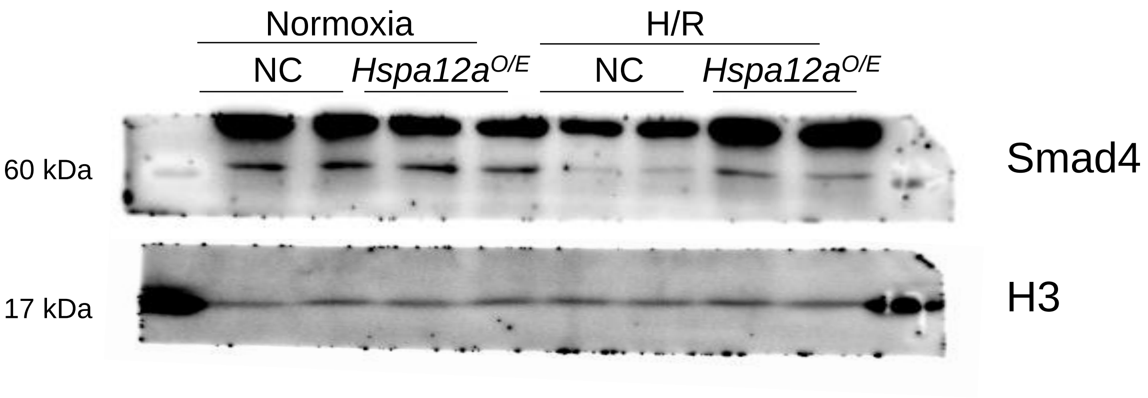

Full unedited gel for Supplement Figure S15

A

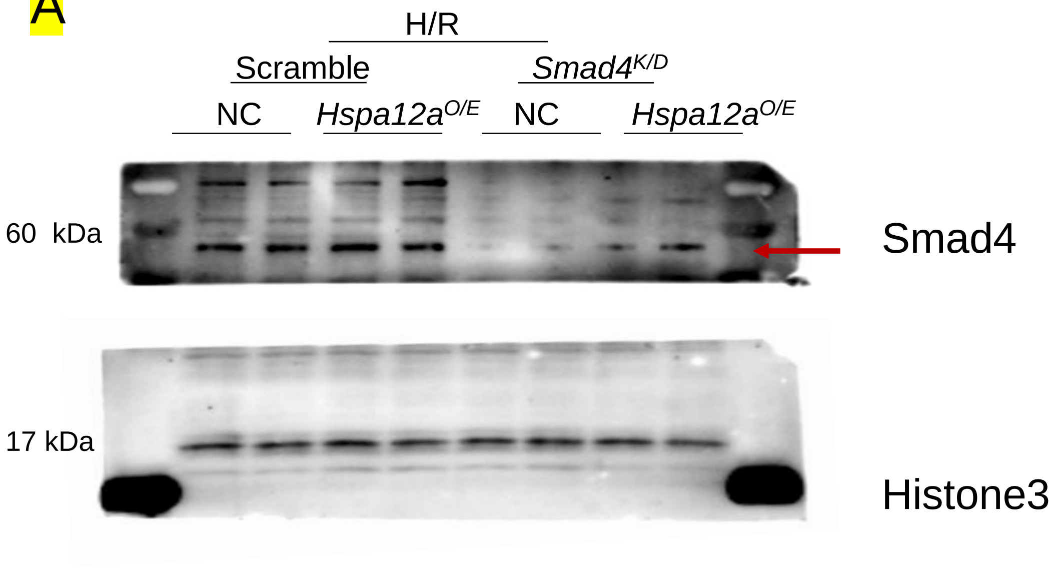

B

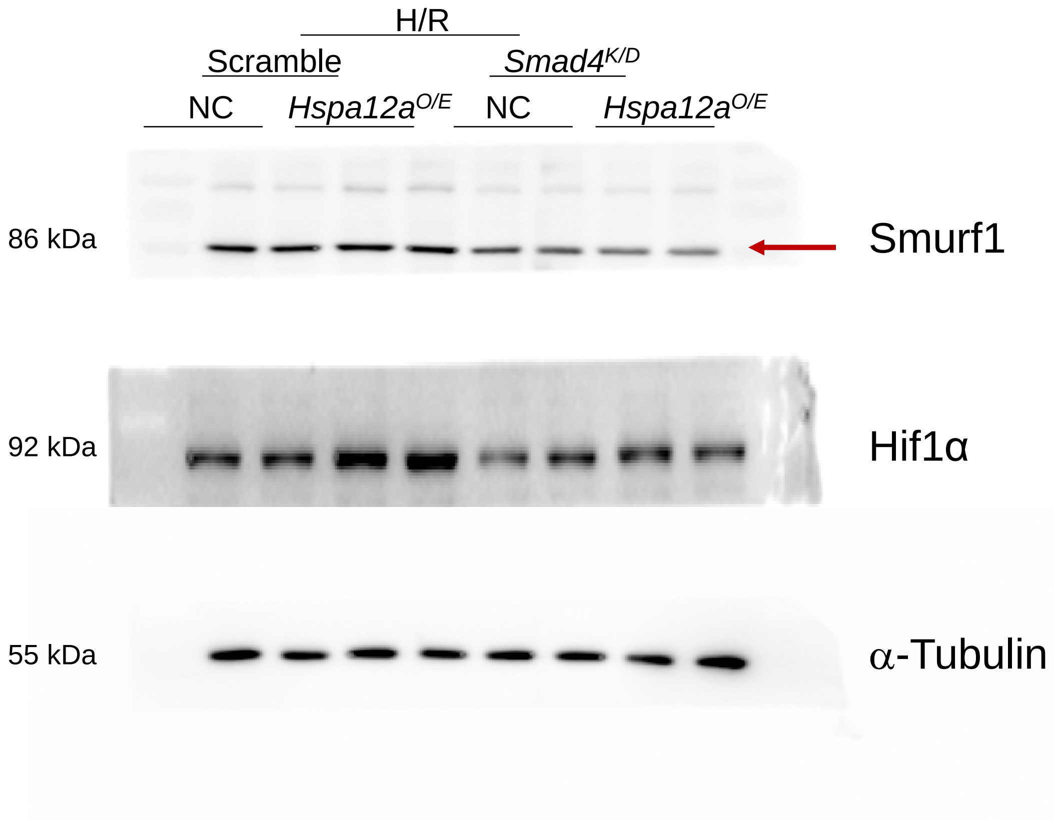

Full unedited gel for Supplement Figure S15

C

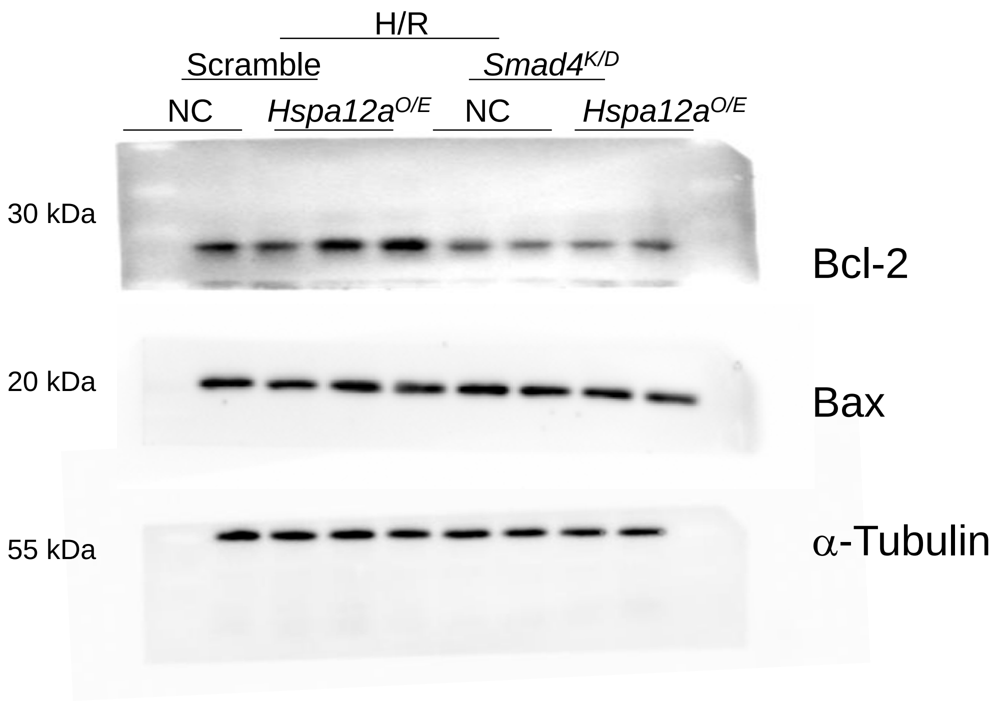

Supplement: Unedited blot and gel images [file jciinsight-9-169125-s252.pdf]
